# Multi-Energy Conversion and Electromagnetic Shielding Enabled by Carbonized Polyimide/Kevlar/Graphene Oxide@ZIF-67 Bidirectional Complex Aerogel-Encapsulated Phase-Change Materials

**DOI:** 10.1007/s40820-025-01761-w

**Published:** 2025-04-27

**Authors:** Tao Shi, Xing Gao, Huan Liu, Xiaodong Wang

**Affiliations:** State Key Laboratory of Organic–Inorganic Composites, Beijing University of Chemical Technology, Beijing, 100029 People’s Republic of China

**Keywords:** Carbonaceous aerogel, Phase-change composites, Bidirectional porous structure, Solar-thermal energy conversion, Thermoelectric generation

## Abstract

To address the limitations of conventional energy systems and optimize the energy conversion pathways and efficiency, a type of “five-in-one” multifunctional phase-change composite with magnetothermal, electrothermal, solar-thermal, and thermoelectric energy conversion and electromagnetic shielding functions is developed for multipurpose applications. Such a novel phase-change composite is fabricated by an innovative combination of paraffin wax (PW) as a phase-change material and a carbonized polyimide/Kevlar/graphene oxide@ZIF-67 complex aerogel as a supporting material. The carbonized complex aerogel exhibits a unique bidirectional porous structure with high porosity and robust skeleton to support the loading of PW. The reduced graphene oxide and CoNC resulting from high-temperature carbonization are anchored on the aerogel skeleton to generate high thermal conduction and magnetic effect, enhancing the phonon and electron transfer of the aerogel and improving its energy conversion efficiency. The phase-change composite not only exhibits excellent solar-thermal, thermoelectric, electrothermal, and magnetothermal energy conversion performance, but also achieves high electromagnetic interference shielding effectiveness of 66.2 dB in the *X*-band. The introduction of PW significantly improves the thermal energy-storage capacity during multi-energy conversion. The developed composite exhibits great application potential for efficient solar energy utilization, sustainable power generation, outdoor deicing, human thermal therapy, and electronic device protection.

**Supplementary Information:**

The online version contains supplementary material available at 10.1007/s40820-025-01761-w.

## Highlights

- A “five-in-one” multifunctional phase-change composite is developed for magnetothermal, electrothermal, solar-thermal, and thermoelectric energy conversion and electromagnetic shielding applications.
- The developed composite is based on an innovative combination of carbonized polyimide/Kevlar/graphene oxide@ZIF-67 complex aerogel as a supporting material and paraffin wax as a phase-change material.
- The developed composite exhibits excellent solar-thermal, thermoelectric, electrothermal, and magnetothermal energy conversion performance along with high electromagnetic interference shielding effectiveness.

**Supplementary Information:**

The online version contains supplementary material available at 10.1007/s40820-025-01761-w.

## Graphical abstract

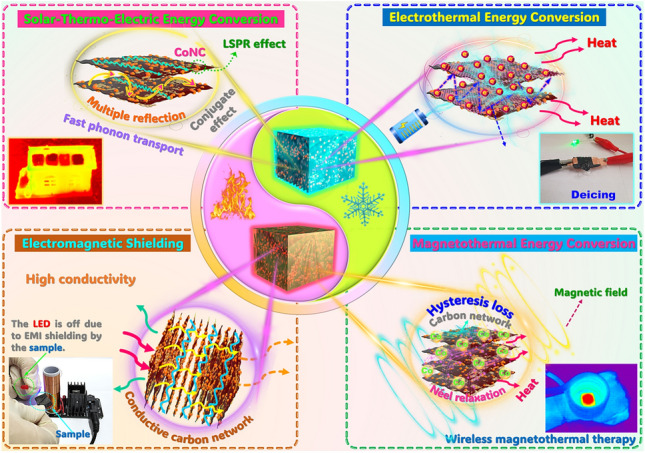

## Introduction

The efficient utilization of clean energy, such as solar, wind, hydro, geothermal, and nuclear energy, becomes a paramount concern as the world grapples with the challenges of finite fossil fuel resources and climate changes [1]. A clean energy revolution is happening all over the world, and its critical use lies in the energy storage and conversion. The rational energy-storage strategy enables the transformation of intermittent clean energy sources as usable forms to make them more reliable and accessible [2, 3]. The form of energy conversion not only expands the potential applications of clean energy, but also determines the utilization efficiency and effectiveness of clean energy [4, 5]. For example, the solar-thermal energy conversion allows sunlight to be converted into thermal energy and then used for heating purposes [6]. The electrothermal conversion can transform electrical energy into thermal energy, which is essential for industrial and domestic applications. A higher energy conversion efficiency can absorb more amounts of clean energy from renewable sources and minimize the energy loss during the energy-storage process to reduce the need of additional energy inputs [7]. Therefore, the development of advanced functional energy materials and relevant technologies for clean energy storage with high conversion efficiency and multiple conversion paths is crucial for consistent and reliable energy supply [8].

As a type of promising latent heat storage material, phase-change materials (PCMs) have received increasing attention in recent years due to their great application potential in clean energy conversion and storage. They have the unique ability to store a large amount of thermal energy as latent heat and release it through reversible phase transitions [9]. With high chemical stability, large latent heat capacity, and good durability, PCMs have been developed to satisfy multipurpose applications such as heat treatment packaging of electronic devices [10], energy-efficient construction [11], radiative cooling [12, 13], and synchronous visual/infrared thermal stealth [14]. However, PCMs have two inherent limitations, including low thermal conductance and high fluidity in the molten state [15]. The low thermal conductance of PCMs impedes their heat transfer, suppressing their energy conversion, storage, and release. On the other hand, the high fluidity of the molten PCMs leads to poor shape/form stability and easy leakage in use, thus reducing their energy-storage capacity during the long-term processes of latent heat storage and release [16]. Therefore, PCMs need to be modified before being utilized in the field of clean energy conversion and storage [17]. Aerogels are normally employed to combine with PCMs to prevent against the fluidity, exudation, and leakage of PCMs as well as enhance their thermal conductance [18]. Aerogels have a highly porous structure to load PCMs and confine them within their frameworks through a capillary force and a weak bonding interaction [19]. Moreover, through rational functionalization, the resultant aerogels can achieve different energy conversion pathways, such as solar-thermal, electrothermal, and magnetothermal conversion. There have been different types of aerogels, such as organic, inorganic, and biomass aerogels, developed for loading with PCMs [20]. Among these aerogels, inorganic aerogels have great superiorities over other types of aerogels in thermal conductance and solar light absorption due to their highly thermal conductive skeletons and excellent sunlight absorption capability, which can accelerate thermal conduction and heat transfer within PCMs and improve solar-thermal conversion [21]. However, inorganic aerogels have a brittle nature, which easily causes damages in their continuous porous structure. Organic aerogels exhibit the characteristics of highly porous structure and good mechanical properties, enabling them to load more amounts of PCMs and also maintain their porous structural integrity after being loaded with PCMs [22]. Nevertheless, organic aerogels have some shortcomings like low thermal conductance and poor sunlight absorption. Although the introduction of nanofillers is considered as an effective method to enhance the performance of organic aerogel/PCM composites, a large addition amount of nanofillers is always required for this method to obtain a desired enhancement effect, following by a reduction in the energy-storage capacity of the phase-chance composites [23].

Carbonization strategy is another approach to boosting the thermal transfer performance of organic aerogels. The carbonaceous aerogels produced from biomass polymers [24], phenolic polymers [25], and melamine foams [26] are highly attractive as supporting matrixes for shape stabilization of PCMs. In addition to the adjustable shapes resulting from their skeletons formed by carbonization, carbonaceous aerogels usually possess an excellent sunlight absorption capability and high thermal conductivity and therefore are suitable for the application of solar-thermal energy conversion [27]. For example, Song et al. [28] fabricated an aerogel/PCM composite through a combination of carbonized biomass loofah sponge with polyethylene glycol (PEG). The resultant composite obtained a high latent heat storage density of 137.6 J g^−1^ and outstanding solar-thermal conversion performance. Shu et al. [29] prepared a high-quality anisotropic graphene aerogel for encapsulating paraffin wax (PW) as a PCM and the resultant composite obtained a high-light absorption rate of 95% and a high photothermal conversion efficiency of 90%. Sun et al. [30] utilized biomass xanthan gum and rigid polyimide (PI) to construct a lightweight and high-strength PI-based carbonaceous aerogel for shape stabilization of PEG. The resultant carbonaceous aerogel gained a photothermal conversion efficiency of over 94.23%. It should be mentioned that organic aerogels usually suffer from significantly shrinking during carbonization, which lowers the thermal energy-storage capacity of the resultant carbonaceous aerogels [30]. Incorporating two-dimensional or one-dimensional nanofillers into organic aerogels prior to carbonization has been considered as a promising way to reduce their shrinkage [31]. These incorporated nanofillers not only restrict the volume shrinkage of carbonaceous aerogels to gain high porosity, but also act as a part of aerogel frameworks to improve their thermal conductance. More importantly, the nanofillers can provide extra functions such as electrical conduction and electromagnetic effectiveness for carbonaceous aerogels, thus expanding the application ranges of the carbonaceous aerogel/PCM composites for solar energy harvesting [32], personal thermotherapy [33], electronic protection [34], and electromagnetic interference (EMI) shielding [35]. However, the introduction of nanofillers inevitably deteriorates the mechanical performance of carbonaceous aerogels. In addition, the current studies mostly focused on the utilization of nanofillers for the carbonaceous aerogel-based phase-change composites with the single function but ignored the development of advanced energy-storage materials with multi-energy conversion [36]. This evidently restricts the application ranges of the carbonaceous aerogel-based composites in advanced energy conversion and storage systems.

**Fig. 1:**
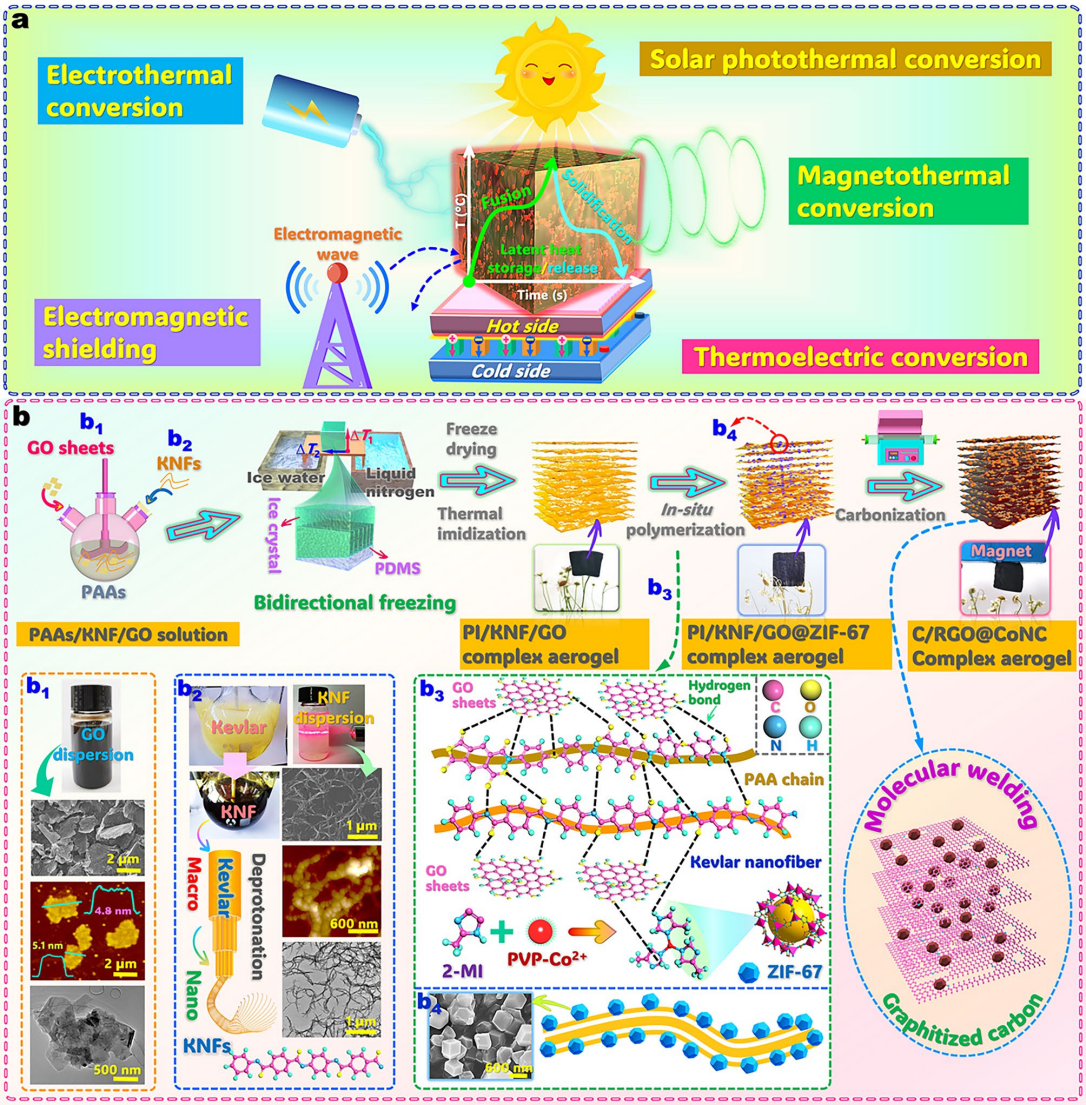
**a** Schematic function of developed C/RGO@CoNC aerogel/PCM composite for multi-energy conversion and electromagnetic shielding. **b** Fabrication strategy and mechanism of PI/KNF/GO@ZIF-67 and C/RGO@CoNC aerogels: **b**_**1**_ Digital photograph of GO nanosheet dispersion and SEM, AFM, and TEM micrographs of GO nanosheets; **b**_**2**_ Digital photographs of KNF dispersion during the preparation process and SEM, AFM, and TEM micrographs of KNF; **b**_**3**_ Intermolecular forces among PAA, KNF, and GO nanosheets and schematic *in situ* depositing strategy of ZIF-67 nanoparticles on carbonized PI/KNF/GO complex aerogel; **b**_**4**_ SEM micrograph of ZIF-67 nanoparticles deposited on the complex aerogel

In the present study, a type of “five-in-one” multifunctional aerogel-based phase-change composite was designed by an innovative integration of PW as an organic PCM and a carbonized PI/Kevlar nanofiber (KNF)/graphene oxide (GO)@ZIF-67 bidirectional complex aerogel (hereafter named as “C/RGO@CoNC aerogel”) as a supporting material for solar-thermal, thermoelectric, electrothermal, and magnetothermal multi-energy conversion and storage together with electromagnetic shielding (Fig. 1a). To obtain such a carbonaceous aerogel, a PI/KNF/GO complex aerogel was first fabricated through unidirectional freeze-drying and thermal imidization. PI has high thermal stability and prominent mechanical performance due to its rigid aromatic structure and therefore is considered as an ideal material for the production of carbonaceous aerogels with excellent mechanical properties. KNF can generate a bridging effect on the carbonized PI aerogel to endow with lightweight and robust characteristics. GO can reinforce the skeleton of the PI aerogel and suppress the shrinkage of the carbonized PI aerogel in the carbonization process owing to its structural characteristics similar to the carbonized PI matrix under high-temperature pyrolysis. Moreover, the high-temperature carbonization process is also advantageous to remove most of the oxygen-containing functional groups on GO to form highly reduced GO (RGO), thus enhancing its graphitization level and thermal conductance [37]. In this sense, the carbonized PI/KNF/GO complex aerogel is anticipative to achieve low shrinkage and high porosity. This enables the carbonized PI-based aerogel to obtain a high loading of PCM as well as a high thermal conductivity for fast thermal response. To further enhance thermal conduction and endow with magnetic effectiveness, ZIF-67 nanoparticles were *in* *situ* deposited onto the skeleton of the PI/KNF/GO complex aerogel prior to carbonization. ZIF-67 can act as self-sacrificing template simultaneously to offer Co, N, and C sources to form a CoNC co-hybrid after high-temperature carbonization. The skeleton-deposited ZIF-67 can provide nitrogen and carbon species after carbonization for the carbonaceous aerogel to enhance its electric conduction. Meanwhile, the metallic Co component resulting from thermal reduction of ZIF-67 can generate electromagnetic effectiveness and localized surface plasmon resonance (LSPR) for the carbonaceous aerogel. In this case, the resultant C/RGO@CoNC aerogel is expected to gain multiple functions of solar-thermal, electrothermal, and magnetothermal energy conversion/storage capabilities as well as an electromagnetic shielding function.

In addition, the multiple functional fillers can provide synergistic interactions to enhance the multifunctional performance of the aerogel-based phase-change composite. First, the conjugated effect of the carbonized skeleton welded with RGO nanosheets and the LSPR effect of CoNC nanoparticles synergistically improve light absorption and localized heating, enabling efficient solar-thermal energy conversion. Second, the interaction between RGO nanosheets and CoNC nanoparticles enhances the electrical and thermal conductivity of the carbonized aerogel, facilitating efficient thermoelectric and electrothermal energy conversion. Third, the refection loss of electromagnetic waves caused by the highly conductive carbonized skeleton and the absorption loss through magnetic hysteresis of Co nanoparticles synergistically boost the overall electromagnetic shielding performance. Compared to traditional single-functional carbonaceous aerogels, the C/RGO@CoNC aerogel developed in this study can create more effective pathways for fabrication of the organic–inorganic composite aerogels with high porosity and external stimuli responses to solar light, electricity, and magnetism. Furthermore, an innovative combination of the C/RGO@CoNC aerogel with PW as a PCM through vacuum impregnation not only generates a high latent heat capacity of over 209 J g^−1^ for the resultant composites to store huge amounts of thermal energy, but also enables them to achieve a multiple energy conversion capability and an electromagnetic shielding function. As shown in Table S1, most of the multifunctional aerogel-based PCM composites reported in the literature only exhibited two or three functions, which did not cover the whole functions of solar-thermal, thermoelectric, electrothermal, and magnetothermal conversion and EMI shielding. In contrast to those reported composites, the developed C/RGO@CoNC/PW composite integrates the above-mentioned five functions within one material by impregnating PW into the carbonized PI-matrix complex aerogel. This may offer an inspiring strategy for the design and development of advanced functional materials with multi-energy conversion and storage capabilities for multipurpose applications.

## Experimental Section

### Materials

4,4’-Diaminodiphenyl ether (ODA, 98.0 wt%), pyromellitic dianhydride (PMDA, 99.0 wt%), *N*, *N*’-dimethylacetamide (DMAc, 99.8 wt%), cobalt nitrate hexahydrate (99.0 wt%), triethylamine (TEA, 99.0 wt%), polyvinylpyrrolidone (PVP, K30) with a number-average molecular weight of 58,000 g mol^−1^, *2*-dimethylimidazole (2-MI) (98.0 wt%), and graphite powder (325 mesh) were purchased from Aladdin Reagent Co., Ltd., China. Sodium nitrate (NaNO_3_, 99 wt%), potassium persulfate (K_2_S_2_O_8_, 99.9 wt%), potassium permanganate (KMnO_4_, 99.5 wt%), sulfuric acid (H_2_SO_4_, 98 wt%), hydrochloric acid (HCl, 37 wt%), and hydrogen peroxide (H_2_O_2_, 30 wt%) were purchased from Beijing Chemical Factory, China. Dimethyl sulfoxide (DMSO, 99.7 wt%) was purchased from Tianjin Heowns Biochemical Technology Co., Ltd, China. Poly(*p*-phenylene terephthamide) (PPTA) fiber (Kevlar®1414) was purchased from DuPont Company. Natural graphite flakes (300 meshes) were commercially provided by Nanjing XFNANO Materials Technology Co., Ltd., China. A PW with a melt point of around 42 °C was purchased from Dongguan Donglin Polymer Material Co., Ltd., China. All of reagents and chemicals were used as received without further purification.

### Fabrication of GO Nanosheets and KNF Dispersion

GO nanosheets were obtained through chemical exfoliation from natural graphite flakes using the modified Hummers method [38]. In a typical procedure, graphite powder (10.0 g) was dispersed in a concentrated H_2_SO_4_ solution (120.0 mL) containing K_2_S_2_O_8_ (15.0 g) and P_2_O_5_ (15.0 g) under agitation at 80 °C for 4 h. The resulting suspension was filtered, washed with deionized water, and dried at 50 °C for 12 h to obtain the pre-oxidized graphite. Afterward, a three-necked round-bottom flask was charged with pre-oxidized graphite powders (5.0 g), NaNO_3_ (5.0 g), and concentrated H_2_SO_4_ (250.0 mL) in an ice bath, and the resultant mixture was mechanically stirred at 0 °C for 1 h, and then, KMnO_4_ (30.0 g) was added slowly into the flask under agitation for 3 h. Subsequently, the ice bath was removed, and an appropriate amount of deionized water and H_2_O_2_ was added slowly into the flask with stirring to allow a reaction at 35 °C for 2 h. The resulting suspension was filtered and centrifuged at 8000 r min^−1^ for 30 min, and the obtained powders were washed with HCl and deionized water several times and then subjected to sonication for 2 h to obtain a dispersion containing GO nanosheets. In this work, four types of GO dispersion with contents of 4, 8, 12, and 16 mg mL^−1^ were prepared for future use.

KNF dispersion was prepared by using a deprotonation method. In a typical procedure, a round-bottom flask was charged with PPTA fiber (2.0 g), KOH (2.0 g), deionized water (8.0 mL), and DMSO (200.0 mL). The resultant mixture was agitated at room temperature for 48 h to obtain a KNF/DMSO dispersion (Fig. 1b_2_). The dispersion was filtered, washed with deionized water several times, and re-dispersed in deionized water to obtain a KNF dispersion with a concentration of 6.0 mg mL^−1^ for future use.

### Fabrication of PI/KNF/GO@ZIF-67 Complex Aerogels

A series of PI/KNF/GO complex aerogels with GO contents of 0, 5, 10, 15, and 20 wt% were fabricated by using water-soluble polyamic acid ammonium salt (PAAs) as a polyimide precursor, KNF as a disperse phase, and GO nanosheets as a functional filler. PAAs were prepared by using the method reported in previous study [39]. In a typical procedure, solid PAAs (0.8 g), KNF dispersion (15.0 mL), deionized water (13.0 mL), TEA (2.0 mL), and GO dispersion (16 mg mL^−1^, 10.0 mL) were added into a round-bottom flask with stirring at room temperature for 5 h to obtain a homogeneous suspension (Fig. 1b). Afterward, the resulting suspension was poured into a cubic polytetrafluoroethylene mold with dimensions of 30 × 30 × 40 mm^3^, and then, the mold was placed on a copper platform immersed with liquid N_2_. The bottom of the mold was installed with a piece of polydimethylsiloxane sheet with a wedge angle of 15°. After the homogeneous suspension was completely solidified, the mold was placed in a freeze-dryer at − 80 °C under a vacuum degree of 1–3 Pa for 96 h to ensure complete sublimation of ice cylinders from the solidified sample. Finally, a thermal imidization process was carried out at 165 °C for 50 min and 320 °C for 140 min in nitrogen to obtain the PI/KNF/GO-20 complex aerogel. To decorate ZIF-67 nanoparticles on the skeleton of the PI/KNF/GO-20 complex aerogel, PVP (0.30 g) and Co(NO_3_)_2_·6H_2_O (0.58 g) were dissolved in methanol (30.0 mL) to obtain a homogenous solution, and then, the PI/KNF/GO-20 complex aerogel was immersed in the solution under agitation for 30 min and stood for 5 h to adsorb Co ions. *2*-MI (1.23 g) was dissolved in methanol (30.0 mL) and then added dropwise into the solution with stirring for 30 min in an ice bath, followed by aging at room temperature for 24 h. The resulting aerogel was washed with methanol and deionized water several times and then dried in a vacuum oven at 80 °C for 10 h to obtain the PI/KNF/GO-20@ZIF-67 complex aerogel.

### Preparation of C/RGO@CoNC Aerogels and Their Composites with PW

The as-prepared PI/KNF/GO@ZIF-67 complex aerogels were placed in a tube furnace and then carbonized at 1000 °C for 2 h with a heating rate of 5 °C min^–1^ under a nitrogen atmosphere to obtain the C/RGO@CoNC aerogels. In addition, the PI/KNF complex aerogel without GO and ZIF-67 was also carbonized as a control under identical conditions and named as the carbonized PI/KNF aerogel for comparison. With the successful fabrication of the C/RGO@CoNC@ZIF-67 aerogels, PW was encapsulated into the aerogels by using a vacuum impregnation method. In a typical procedure, PW was heated at 80 °C for 3 h to melt completely and then the C/RGO@CoNC aerogels were placed in the molten PW under vacuum for 8 h. After the completion of impregnation, the C/RGO@CoNC/PW composites were subjected to natural cooling. The resultant composites were wiped with a filter paper to remove the residual PW from their surfaces.

### Analysis and Measurement Methods

Details for the methods of structural characterizations, performance measurements, and numerical simulation are available in Sections S1 and S2.

## Results and Discussion

### Fabrication Strategy and Microstructure of Bidirectional Complex Aerogels

The PI/KNF/GO@ZIF-67 complex aerogel was first fabricated through bidirectional freezing, freeze drying, and thermal imidization using PI as a skeleton, GO and ZIF-67 as functional fillers, and KNF as a bridging agent. Then, the resulting complex aerogel was carbonized under an inert atmosphere to obtain the C/RGO@CoNC aerogel (Fig. 1b). In the fabrication procedure of the PI/KNF/GO@ZIF-67 complex aerogel, GO nanosheets and KNF were first prepared through the modified Hummers and deprotonation methods, respectively. The exfoliated GO nanosheets show a lateral size of 2–6 μm and a thickness of 5 nm according to the scanning electron microscopy (SEM), atomic force microscopy (AFM), and transmission electron microscopy (TEM) micrographs shown in Fig. 1b_1_. Figure 1b_2_ shows a change in the color of the KNF dispersion from light yellow to deep red during the chemical deprotonation reaction. This is ascribed to the deprotonation of the aramid groups of KNF, which weakens the hydrogen bonding between its polymeric chains through electrostatic repulsion. Benefiting from the π–π stacking, electrostatic repulsion, and van der Waals force, a stable KNF dispersion was formed [40]. According to the characterization results from SEM, AFM, and TEM, the as-prepared KNF exhibits a length of 3–5 μm along with an average diameter of 45 nm. Such a small size generated a significant Tyndall effect in the KNF dispersion (Fig. 1b_2_).

**Fig. 2:**
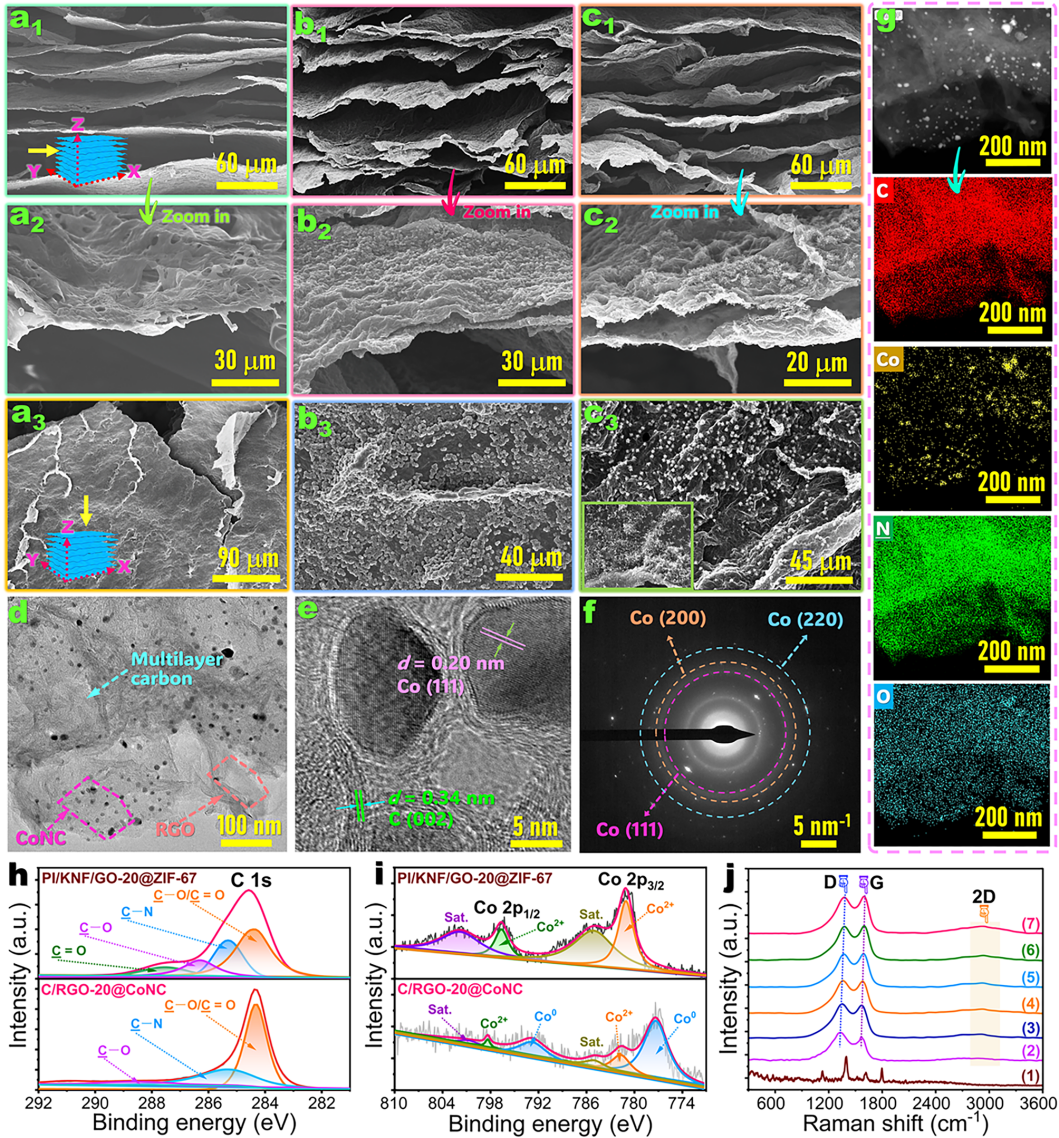
SEM micrographs of the profiles of (**a**_**1**_, **a**_**2**_) PI/KNF/GO-20, (**b**_**1**_, **b**_**2**_) PI/KNF/GO-20@ZIF-67, and (**c**_**1**_, **c**_**2**_) C/RGO-20@CoNC aerogels in the *Y–Z* plane along the freezing direction and the cross sections of **a**_**3**_ PI/KNF/GO-20, **b**_**3**_ PI/KNF/GO-20@ZIF-67 and **c**_**3**_ C/RGO-20@CoNC aerogels in the *X–Y* plane along the freezing direction. **d**, **e** High-resolution TEM micrographs, **f** SAED micrograph, and **g** elemental mapping images of C/RGO-20@CoNC aerogel. High-resolution core-level XPS spectra of PI/KNF/GO-20@ZIF-67 and C/RGO-20@CoNC aerogels in specific binding energy of **h** C 1*s* and **i** Co 2*p*. **j** Raman spectra of (1) PI/KNF/GO-20@ZIF-67, (2) carbonized PI/KNF, (3) C@CoNC, (4) C/RGO-5@CoNC, (5) C/RGO-10@CoNC, (6) C/RGO-15@CoNC, and (7) C/RGO-20@CoNC aerogels

A series of PAAs/GO nanosheet/KNF aqueous dispersions with different GO contents were prepared as aerogel precursors through high-speed homogenization. The *Fourier* transform infrared (FTIR) spectrum shows that there is an abundance of oxygen-containing groups on GO nanosheets, including O–H, C=O, and C–O bonds (Fig. S1a). This enables GO nanosheets to form intermolecular hydrogen bonding with PAAs and KNF. The hydrogen bonding interaction among PAAs, KNF, and GO nanosheets as well as the π–π stacking can help the mixture achieve a stable colloidal dispersion (Fig. 1b_3_). A bidirectional freezing–freeze drying–thermal imidization process was conducted for the resulting colloidal dispersions to fabricate the PI/KNF/GO complex aerogels with a unique bidirectional and lamellar porous structure (Figs. 2a and S2a). The complex aerogels obtained from the precursors containing 0, 5, 10, 15, and 20 wt% GO were hereafter named as the PI/KNF, PI/KNF/GO-5, PI/KNF/GO-10, PI/KNF/GO-15, and PI/KNF/GO-20 aerogels, respectively. The codenames of the as-prepared complex aerogel samples and their composites with PW are listed in Table S2. As shown in Fig. S3, the PI/KNF/GO-20 aerogel as a representative sample can easily recover its original shape from compression at a 1.0-kg force. This suggests a robust skeleton of the complex aerogel to load ZIF-67 nanoparticles. In this case, Co^2+^ as a ZIF-67 precursor was self-assembled on the skeleton of the PI/KNF/GO complex aerogel through coordination (Fig. 1b_4_). Then, ZIF-67 nanoparticles were nucleated onto the skeleton surface with the addition of *2*-MI, causing the formation of the PI/KNF/GO@ZIF-67 complex aerogel. PVP as a surfactant can coordinate with Co^2+^ to prevent against the aggregation and overgrowth of ZIF-67 nanoparticles. This ensures ZIF-67 nanoparticles uniformly anchored onto the skeleton of PI/KNF/GO@ZIF-67 complex aerogel (Fig. 2b). This results in a rougher skeleton surface in contrast to the PI/KNF/GO complex aerogel (Fig. S2b). The TEM micrographs further confirm the successful deposition of GO nanosheets and ZIF-67 nanoparticles on the skeleton surface of the PI/KNF/GO@ZIF-67 aerogel (Fig. S4).

The PI/KNF/GO complex aerogel was carbonized under high temperatures to form the C/RGO@CoNC aerogel. Similar to the PI/KNF/GO@ZIF-67 complex aerogel, the C/RGO@CoNC aerogel exhibits a parallel lamellar porous structure (Figs. 2c and S2c). It should be noted that there is a little shrinkage on the skeleton of the C/RGO@CoNC aerogel. High-temperature carbonization was also performed for pure GO nanosheets and ZIF-67 nanoparticles to confirm their chemical transformation into RGO and CoNC. As seen in the FTIR spectra (Fig. S1a), the as-prepared RGO presents a weakening trend in its characteristic absorption peaks of oxygen-containing groups along with an enhancement in the intensity of characteristic bands for the C–O (1060 cm^–1^) and C=C (1557 cm^–1^) bonds in contrast to pure GO. The *X*-ray diffraction (XRD) patterns show that after carbonization of GO, there is a shift of 2θ from 9.6° to 24.1° for the diffraction peak corresponding to the (002) plane (Fig. S1b). The high-resolution TEM micrographs also demonstrate the presence of RGO nanosheets and CoNC nanoparticles on the skeleton of the C/RGO@CoNC aerogel (Fig. 2d, e). These results suggest that high-temperature carbonization is conducive to the restoration of a graphene structure during the reduction of GO. The graphene structure of RGO can form a high-effective heat transfer pathway for the C/RGO@CoNC aerogel to gain high thermal conductance. Prior to high-temperature carbonization, ZIF-67 nanoparticles exhibit a purple color and a dodecahedral structure, and there is no magnetic effectiveness observed from them (Fig. S5a, b). It is interesting to note a change in the color from purple to black after carbonization of ZIF-67 nanoparticles (Fig. S5a), and their surfaces are also shrunken due to the presence of CoNC co-hybrid nanoparticles (Fig. S5c). The formed CoNC nanoparticles show a strong magnetic response to a lodestone, indicating that the magnetic graphite carbon can be generated by calcination of the ZIF-67 nanoparticles containing magnetic metal [41]. The XRD patterns also demonstrate a transformation of crystalline structure occurred in ZIF-67 nanoparticles before and after carbonization (Fig. S6). Such a crystalline transformation can further be confirmed by the high-resolution TEM micrograph and selective area electron diffraction (SAED) micrograph of the C/RGO@CoNC aerogel (Fig. 2e, f). It should be noted that the C/RGO@CoNC aerogel can also be attracted on the lodestone (Fig. 1b). These results indicate that the carbonization of ZIF-67 provides the C/RGO@CoNC aerogel with good magnetic effectiveness.

### Chemical Structures of Complex Aerogels

The C/RGO@CoNC aerogel was investigated by FTIR, energy-dispersive *X*-ray (EDX) spectroscopy, and *X*–ray photoelectron spectroscopy (XPS) to determine its chemical compositions and structure. The FTIR spectrum of the PI/KNF/GO-20@ZIF-67 aerogel as a representative sample shows a series of characteristic absorption peaks corresponding to the N–H (1141 cm^–1^), C=O (1660 and 717 cm^–1^), N–H (1140 cm^–1^), C=C (1496 cm^–1^), and C–N (1373 cm^–1^) bonds in KNF and PI (Fig. S7). There is no distinct difference in these characteristic peaks between the complex aerogels before and after introduction of GO nanosheets and ZIF-67 nanoparticles. It is worth noting that there are only two characteristic bands for the carboatomic ring (C=C, 1515 cm^−1^) and C–O bond (1195 cm^−1^) in the spectrum of the C/RGO-20@CoNC aerogel [42]. EDX and XPS spectra can also confirm a change in the chemical structure of the complex aerogel derived from carbonization. The Co, N, C, and O elements are clearly identified by the EDX spectra and the corresponding elemental mapping images of the PI/KNF/GO-20@ZIF-67 (Figs. S8 and S9) and C/RGO-20@CoNC aerogels (Figs. S10 and 2g). Compared to the PI/KNF/GO-20@ZIF-67 aerogel, the C/RGO-20@CoNC aerogel presents higher C and Co contents due to the carbonization that leads to a decrease in the O and N contents. The survey XPS spectra of the PI/KNF/GO-20@ZIF-67 and C/RGO-20@CoNC aerogels also demonstrate the presence of the C, O, N, and Co elements (Fig. S11). More importantly, in contrast to the PI/KNF/GO-20@ZIF-67 aerogel, the C/RGO-20@CoNC aerogel exhibits a higher *sp*^2^ C–C ratio in its high-resolution core-level XPS spectrum of C 1*s* (Fig. 2h), indicating that more graphite components are formed by carbonization. The high-resolution core-level XPS spectrum of Co 2*p* suggests the presence of the Co element in the complex aerogels after carbonization (Fig. 2i), which is ascribed to a reduction reaction of Co^2+^/Co^3+^ with C. The presence of metallic Co enables the C/RGO@CoNC aerogel to obtain magnetic effectiveness.

Raman and XRD spectroscopy were conducted to evaluate the graphitization level of the C/RGO@CoNC aerogel. In contrast to the PI/KNF/GO@ZIF-67 complex aerogel, the carbonized aerogels show three characteristic peaks centered at 1340, 1585, and 2935 cm^−1^, corresponding to the D, G, and 2D bands of graphene, respectively, in their Raman spectra (Fig. 2j). This result indicates the occurrence of defective crystalline carbon and crystalline graphite (in-plane *sp*^2^ vibration of carbon atoms) in the carbonized aerogels. With an increase in the GO content, the intensity ratio of D to G bands (I_D_/I_G_) decreases slightly (Fig. S12). In particular, the I_D_/I_G_ values of the C/RGO-20 and C/RGO-20@CoNC aerogels were determined to be 1.13 and 0.94, respectively. A lower *I*_D_/*I*_G_ value indicates a greater amount of ordered graphite [43], suggesting that the introduction of GO is advantageous to the formation of high-quality graphite. The XRD patterns of the carbonized aerogels show a characteristic diffraction peak at 25.5°, assigned to the (002) plane of graphite (Fig. S13). This characteristic diffraction was found to shift to 26.2° with an increase in the GO content from 0 to 20 wt%, demonstrating that a high GO content can promote the formation of highly ordered graphite and thus enhance the thermal and electrical conduction of the carbonized aerogels [44]. In addition, there are three diffraction peaks of the metallic Co appearing at 44.4°, 51.6°, and 75.8°, assigned to its (111), (200), and (220) reflections (JCPDS No. 15-0806), respectively. These results demonstrate that a highly carbonized aerogel along with the metallic Co has been successfully formed after carbonization of the PI/KNF/GO@ZIF-67 complex aerogel.

The porosity of aerogels dominates their capacity to load PCMs in general, and it can be evaluated by mercury intrusion porosimetry. Figure S14a–c shows the mercury intrusion curves of the PI/KNF, PI/KNF/GO-20@ZIF-67, and C/RGO-20@CoNC aerogels as three representative samples, and the corresponding porous parameters are summarized in Table S3. It is of importance to note that a significant increase takes place in the total mercury intrusion volume at a pore size of over 25 μm, demonstrating that these complex aerogels have a macroporous characteristic with a pore-size distribution between 30 and 120 μm. In contrast to the PI/KNF and PI/KNF/GO-20@ZIF-67 aerogels, the carbonized aerogel samples exhibit a slight decrease in their porosity (Table S3). This is ascribed to the shrinkage of aerogel skeleton during carbonization at high temperature. Nevertheless, the C/RGO-20@CoNC aerogel only shows a slight decrease in its porosity by 0.74% in contrast to the PI/KNF/GO-20@ZIF-67 one. The porosity of the carbonized aerogels was found to increase with an increase in the GO content. These results suggest that the introduction of GO can effectively prevent against the shrinkage of aerogel skeleton, thus forming high porosity for the carbonized aerogels to maximize their capacity for loading PCMs. The mechanical properties of carbonized aerogels were determined by uniaxial compression experiments. The resulting stress–strain curves are shown in Fig. S15. The carbonized aerogels exhibit a considerable increase in ultimate compression strength with an increase in the GO content. Specifically, the C/RGO-20@CoNC aerogel exhibits compressive strength of 61.0 kPa, which is much higher than that of the C@CoNC aerogel (22.9 kPa). A high content of GO nanosheets can form a continuous reinforcement network within the carbonized aerogel and establish strong interfacial bonding with the carbonized PI/KNF matrix. This effectively transfers stress and prevents crack propagation, hence enhancing the rigidity and compressive strength of the aerogels. With good mechanical performance, the C/RGO-20@CoNC aerogel can provide a strong framework to support the impregnated PW.

###  Structures and Thermophysical Properties of Phase-Change Composites

**Fig. 3:**
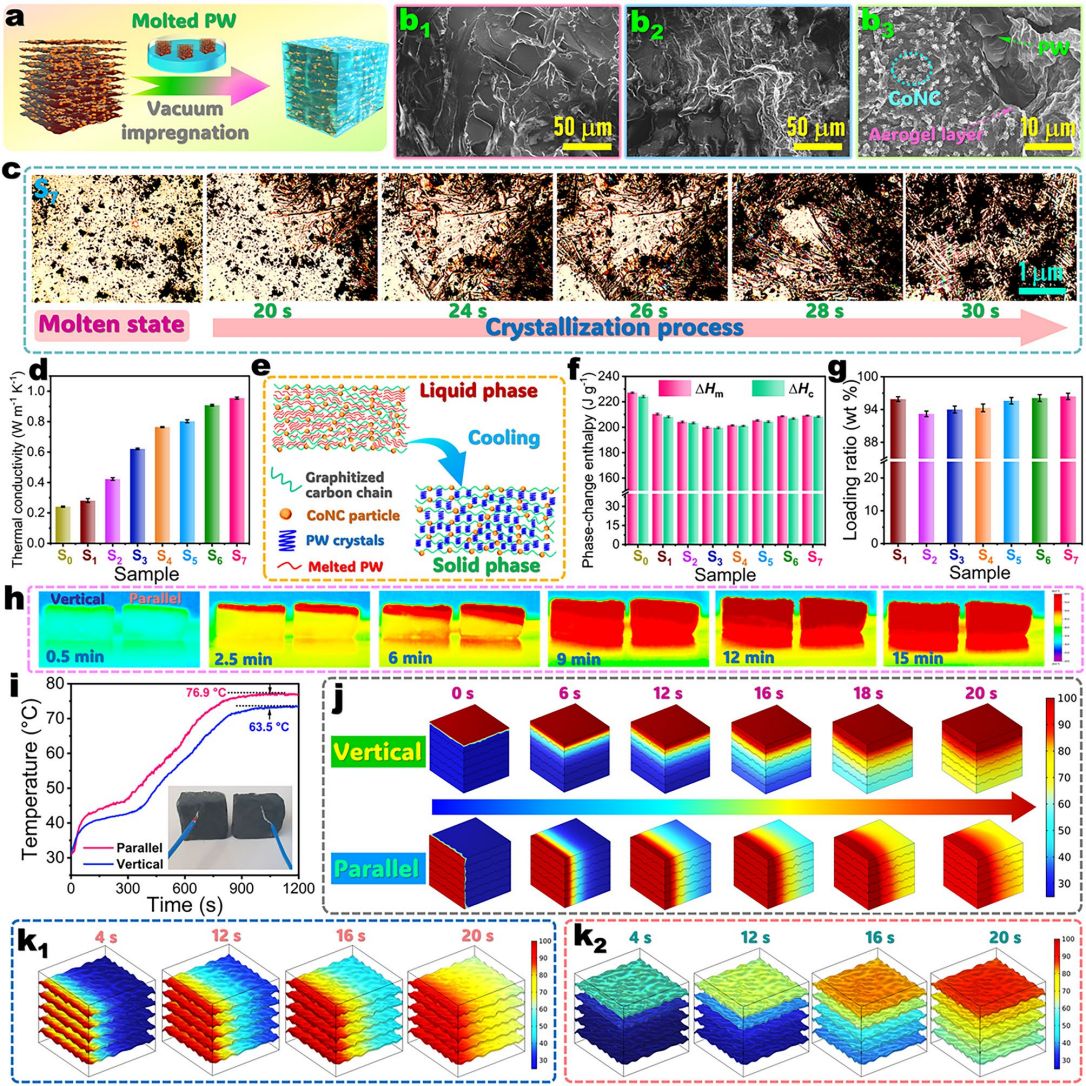
**a** Fabrication strategy of complex aerogel/PW phase-change composites. SEM micrographs of **b**_**1**_ PI/KNF/GO-20/PW, **b**_**2**_ PI/KNF/GO-20@ZIF-67/PW, and **b**_**3**_ C/RGO-20@CoNC/PW composites. **c** Polarized microscope images of C/RGO-20@CoNC/PW composite during the cooling process. **d** Thermal conductivities of (S_0_) pure PW, (S_1_) PI/KNF/GO-20@ZIF-67/PW, (S_2_) carbonized PI/KNF aerogel/PW, (S_3_) C@CoNC/PW, (S_4_) C/RGO-5@CoNC/PW, (S_5_) C/RGO-10@CoNC/PW, (S_6_) C/RGO-15@CoNC/PW, and (S_7_) C/RGO-20@CoNC/PW. **e** Schematic phase-transition mechanism of complex aerogel/PW phase-change composites. **f** Phase-change enthalpies and **g** loading ratios of (S_0_) pure PW and (S_1_) PI/KNF/GO-20@ZIF-67/PW, (S_2_) carbonized PI/KNF aerogel/PW, (S_3_) C@CoNC/PW, (S_4_) C/RGO-5@CoNC/PW, (S_5_) C/RGO-10@CoNC/PW, (S_6_) C/RGO-15@CoNC/PW, and (S_7_) C/RGO-20@CoNC/PW composites. **h** Infrared thermographic images and **i** interior temperature–time evolutions of C/RGO-20@CoNC/PW composite parallel and vertical to the lamellar pores under 1.0 kW m^−2^ solar irradiation. **j** Temperature–time evolutions of the C/RGO-20@CoNC/PW composite parallel and vertical to the lamellar pores obtained from finite element analysis. Temperature–time evolutions of the C/RGO-20@CoNC aerogel **k**_**1**_ parallel and **k**_**2**_ vertical to the lamellar pores obtained from finite element analysis

 A series of complex aerogel/PW composites were fabricated through impregnating the molten PW into the as-prepared complex aerogels under vacuum (Fig. 3a). Figure 3b_1_–b_3_ displays the SEM micrographs of the PI/KNF/GO-20/PW, PI/KNF/GO-20@ZIF-67/PW, and C/RGO-20@CoNC/PW composites as representative samples, demonstrating that PW as a PCM has been successfully impregnated into the complex aerogels through a capillary force and the van der Waals force. Compared to the PI/KNF/GO-20/PW and PI/KNF/GO-20@ZIF-67/PW composites, the C/RGO-20@CoNC/PW composite exhibits a much rougher surface due to the existence of CoNC nanoparticles. The EDX spectrum and corresponding elemental mapping images clearly identify the existence of Co, N, C, and O elements in the C/RGO-20@CoNC/PW composite with a homogeneous distribution (Fig. S16). It is noteworthy that the C/RGO-20@CoNC/PW composite has a remarkable higher C atomic percentage than the C/RGO-20@CoNC aerogel due to the presence of PW as a main component. Moreover, the FTIR spectra of the composite samples show a series of typical characteristic absorption bands of PW (C–H: 2918, 1473, and 1106 cm^−1^) in addition to those of the corresponding complex aerogels, and their XRD patterns reveal a set of characteristic diffraction peaks at 2*θ* = 6.5°, 12.8°, 21.6°, 23.8°, and 39.8°, assigned to the (002), (004), (101), (102), and (− 110) planes of PW (ICDD-PDF 53–1533), respectively (Fig. S17). These diffraction peaks are in good agreement with those of pure PW. These characterization results confirm the successful encapsulation of PW within the complex aerogels through physical interactions without any chemical reactions.

Differential scanning calorimetry (DSC) was conducted to investigate the phase-change thermophysical properties of the complex aerogel/PW composites. As seen in the obtained DSC thermograms (Fig. S18), both pure PW and its composites present a unimodal endothermic behavior and an exothermic behavior appearing in the heating and cooling processes, respectively. The polarized microscope images show that the molten PW loaded in the C/RGO-20@CoNC/PW composite tends to form uniform cross-needle crystals during the cooling process (Fig. 3c). This phenomenon is consistent with the crystalline behavior of pure PW (Fig. S19). These results suggest that there is no effect of the aerogels on the phase-change behaviors of the loaded PW. It should be noted that there is a slight fluctuation in the phase-change temperatures, *i.e.*, melting temperature (*T*_m_) and crystallization temperature (*T*_c_), of the complex aerogel/PW composites (Fig. S20 and Table S4). This may be caused by the aerogel skeletons. Both the PI/KNF/GO and carbonized PI/KNF complex aerogels have lower thermal conductance than the C/RGO@CoNC one. This increases the interfacial thermal resistance between the aerogel framework and PW, leading to higher *T*_m_’s of the complex aerogel/PW composites accordingly (Fig. 3d). The introduction of GO and ZIF-67 improves the thermal conductance of the carbonized aerogel/PW composites effectively. As a result, the C/RGO-20@CoNC/PW composite obtained an enhanced thermal conductivity of 0.96 W m^–1^ K^–1^. This result is 242.9% and 300.0% higher than those of the carbonized PI/KNF aerogel/PW composite and pure PW, respectively. The high thermal conduction of aerogel skeleton can facilitate a heat transfer from the carbonized aerogels to PW (Fig. 3e) and therefore results in a slight decrease in the *T*_m_ of the carbonized aerogel/PW composites. On the other hand, the aerogel skeleton can provide a large number of nucleation cites for PW to promote its heterogeneous nucleation in the cooling process, thus leading to a slight increase in the *T*_c_ of the aerogel/PW composites. It is worth noting that all of the aerogel/PW composites present lower phase-change enthalpies, *i.e.*, melting enthalpy (Δ*H*_m_) and crystallization enthalpy (Δ*H*_c_), than pure PW (Fig. 3f and Table S4). This is due to the presence of inert aerogels without a phase-transition function for latent heat absorption and release. However, the C/RGO-20@CoNC/PW composite exhibits the highest phase-change enthalpies (Δ*H*_m_ = 209.2 J g^–1^ and Δ*H*_c_ = 208.4 J g^–1^) among the carbonized aerogel/PW composites thanks to its highest porosity. Higher porosity offers a larger volume capacity for the aerogel to load more quantity of PW, thus resulting in a higher loading ratio and energy-storage efficiency of the C/RGO-20@CoNC/PW composites (Figs. 3g and S21). It is noteworthy that all of the aerogel/PW composites present a relative high enthalpy efficiency of over 94% (Fig. S22). This demonstrates that the PW loaded within the complex aerogels obtained a high degree of crystallinity to meet a requirement for thermal energy storage and release.

The thermal stability of pure PW and aerogel/PW composites was determined by thermogravimetric analysis (TGA) to confirm the safety applications of the developed composites. A typical one-step thermal degradation behavior in the temperature range of 280–430 °C is observed from the TGA thermograms of pure PW and aerogel/PW composites (Fig. S23). The maximum mass loss temperature of aerogel/PW composites are fluctuated around 390 °C, which are in good agreement with that of pure PW (386 °C). This result suggests that the aerogel samples have little influence on the thermal degradation of PW to meet the application requirement under high-temperature operation conditions. An isothermal heat-impact experiment was conducted to evaluate the form stability and leakage resistance of aerogel/PW composites as well as pure PW used as a control sample. Figure S24 shows the digital photographs of pure PW and aerogel/PW composites recorded during the isothermal heating process at 120 °C. Pure PW was found to lose its original shape during the isothermal heating process because the heating temperature is higher than the *T*_m_ of PW, resulting in the solid-to-liquid phase transition. However, all the aerogel/PW composite samples were found to keep their initial shapes during the heating process. Only a very limited leakage of PW is observed from bottom of the composites after the heating process, which is ascribed to the fusion of PW attached to their surfaces. This result demonstrates that the carbonized aerogel skeleton can provide effective protection against the leakage of the loaded PW under high-temperature impact, contributing good shape stability and anti-leakage property for the composites. To investigate the mechanical performance of aerogel/PW composites, a uniaxial compression experiment was conducted and the corresponding result is presented in Fig. S25a. The composites exhibited a slight increase in compressive strength with minimal fluctuations in comparison with pure PW. This result indicates that the skeleton of aerogel can provide effective support for PW to achieve structural stability. Figure S25b shows a high hardness of the composites ranging from 40 to 44 HD, suggesting that the composites are capable of withstanding significant pressure without easily deforming or getting damaged to ensure structural stability.

The phase-change reversibility of the C/RGO@CoNC/PW composites was characterized by conducting 200 cyclic DSC scans to assess its long-term thermal stability and phase-change reversibility over extended cycles. As shown in the DSC thermograms of the C/RGO-20@CoNC/PW composite as a representative sample in the cyclic heating/cooling process (Fig. S26), these DSC thermograms are in coincidence with each other in their endothermic and exothermic peaks (Fig. S26a). All of them show almost the same location and intensity from the first cycle to the last. The phase-change temperatures and enthalpies of the composite were found to fluctuate slightly during the cyclic process (Fig. S26b). Their Δ*H*_m_ and Δ*H*_c_ at the last cycle only changed by ± 0.29% compared to those at the first cycle. The C/RGO-20@CoNC/PW composite exhibits similar FTIR spectra before and after thermal cycles (Fig. S26c), suggesting a good structural stability of the composite after long-term thermal cycles. In addition, the composite shows overlapped TGA thermograms before and after thermal cycles (Fig. S26d). These findings indicate that the developed C/RGO@CoNC/PW composite has excellent long-term phase-change reversibility and thermal stability for practical applications.

### Heat Transfer of Phase-Change Composites

To clarify the effect of an anisotropic structural skeleton on the heat transfer of the C/RGO@CoNC/PW composite, the C/RGO-20@CoNC/PW composite as a representative sample was illuminated by simulated solar light vertical and parallel to the lamellar pores for 20 min at a light intensity of 1.0 kW m^−2^. An infrared thermal imager and a *k*-type thermocouple were employed to record the temperature–time evolutions of the lateral and interior parts of the C/RGO-20@CoNC/PW composite during solar irradiation. The infrared thermal images show that there is a faster change in the temperature of the part parallel to the lamellar pores compared to that vertical to the lamellar pores (Fig. 3h). After solar irradiation for 20 min, the part parallel to the lamellar pores presents an interior temperature of 76.9 °C, which is 3.4 °C higher than that vertical to the lamellar pores (Fig. 3i). This result is in good agreement with the simulated results by the finite element analysis. Furthermore, the simulated infrared thermal images show that the temperature–time evolutions of the parts vertical and parallel to the lamellar pores are both similar to the experimental results (Fig. 3j). After heating for 20 min through simulation, the interior temperature difference between its parts vertical and parallel to the lamellar pores was calculated to be 4.2 °C (Fig. S27), which is very close to the experimental results. These results are attributed to the bidirectional lamellar porous structure within the C/RGO@CoNC aerogel. Such a unique bidirectional porous structure enables the C/RGO-20@CoNC aerogel to obtain a low thermal conductivity of 0.051 W m^–1^ K^–1^ in the part vertical to the lamellar pores (Fig. S28). This result is lower than the part parallel to the lamellar pores (0.059 W m^–1^ K^–1^). In this case, the in-plane heat transfer of the C/RGO@CoNC aerogel is bound to be faster than its out-of-plane one. This conclusion is also confirmed by the heat transfer simulation (Fig. 3k_1_, k_2_). The C/RGO-20@CoNC aerogel shows a faster temperature change in its part parallel to the lamellar pores than that vertical to the lamellar pores. A directional heat transfer can effectively transfer heat from the heat-generating surface to the interior of the complex aerogel/PW phase-change composites. The anisotropic thermal conductivity influences the heat transfer of the aerogel-based phase-change composites during the energy absorption and conversion processes. The anisotropic structure facilitates the directional heat transfer along the parallel to the lamellar skeleton, enabling faster in-plane thermal conduction. The fast thermal conduction ensures rapid phase transition of PW to store thermal energy as latent heat. Therefore, the anisotropic thermal conductivity is crucial to help the C/RGO@CoNC/PW composite respond to the external stimuli promptly for solar-thermal, thermoelectric, electrothermal, and magnetothermal energy conversion and storage. There is a temperature platform observed from the C/RGO-20@CoNC/PW composite in the range of 40.5–44.0 °C during solar irradiation (Fig. 3i). This can be attributed to the thermal energy absorption resulting from a solid-to-liquid phase transition occurring in the loaded PW. It should be mentioned that the carbonized aerogel contains huge amounts of air in the porous structure due to their high porosity. Although the skeleton of the carbonized aerogel has a high intrinsic thermal conductivity of 10–30 W m^–1^ K^–1^, the extremely low thermal conductivity of air (0.026 W m^–1^ K^–1^) results in a low thermal conductivity of the carbonized aerogel (0.059 W m^–1^ K^–1^). When PW is completely impregnated into the carbonized aerogel to fulfill the porous structure, the thermal conductivity of the resulting aerogel-based phase-change composite is determined by PW and the skeleton of the carbonized aerogel. In this case, the aerogel-based phase-change composite exhibited significant increase in the thermal conductivity with the addition of PW.

### Solar-Thermal Energy Conversion and Storage

**Fig. 4:**
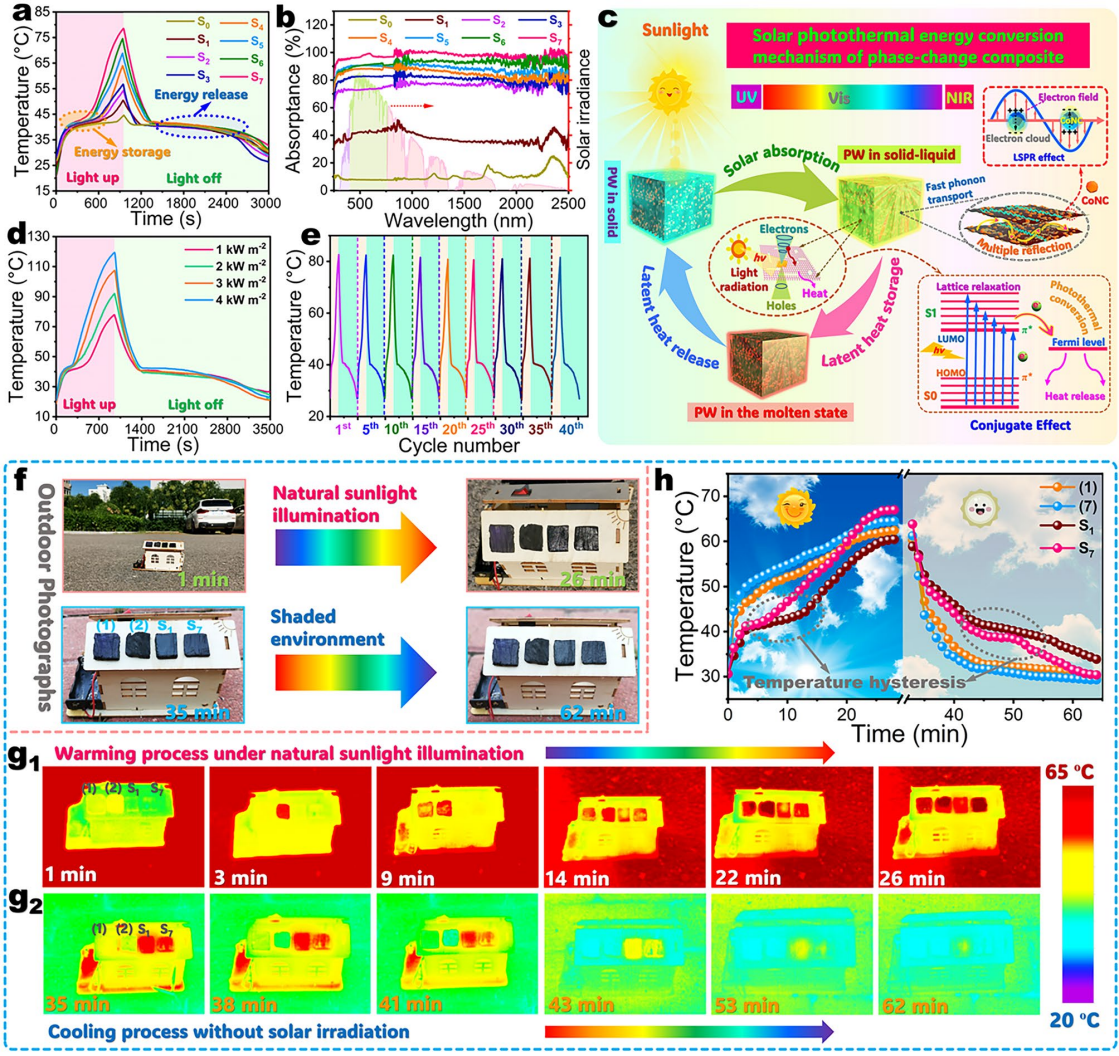
**a** Temperature–time evolutions and **b** Ultraviolet–visible-near-infrared absorption spectra of (S_0_) pure PW and (S_1_) PI/KNF/GO-20@ZIF-67/PW, (S_2_) carbonized PI/KNF aerogel/PW, (S_3_) C@CoNC/PW, (S_4_) C/RGO-5@CoNC/PW, (S_5_) C/RGO-10@CoNC/PW, (S_6_) C/RGO-15 @CoNC/PW, and (S_7_) C/RGO-20@CoNC/PW composites under simulated solar illumination and no irradiation. **c** Scheme of solar-thermal conversion and storage mechanisms of the carbonized aerogel/PW composites. Temperature–time evolutions of C/RGO-20@CoNC/PW composite **d** under simulated solar illumination with different light intensities and no irradiation and **e** during the cyclic process of solar illumination and no irradiation. **f** Digital photographs of a wood house model roof-covered with (1) PI/KNF/GO-20@ZIF-67 and (2) C/RGO-20@CoNC aerogels and (S_1_) PI/KNF/GO-20@ZIF-67/PW and (S_7_) C/RGO-20@CoNC/PW composites under natural sunlight illumination and no solar irradiation. Infrared thermographic images of the wood house model roof-covered with (1) PI/KNF/GO-20@ZIF-67 and (2) C/RGO-20@CoNC aerogels and (S_1_) PI/KNF/GO-20@ZIF-67/PW and (S_7_) C/RGO-20@CoNC/PW composites under **g**_**1**_ natural sunlight illumination and **g**_**2**_ no solar irradiation. **h** Temperature–time evolutions of (1) PI/KNF/GO-20@ZIF-67 and (2) C/RGO-20@CoNC aerogels and (S_1_) PI/KNF/GO-20@ZIF-67/PW and (S_7_) C/RGO-20@CoNC/PW composites under natural sunlight illumination and no solar irradiation

According to the aforementioned experimental and simulation results, the complex aerogel/PW phase-change composites present a faster heat transfer in the part parallel to the lamellar pores than that vertical to the lamellar pores. In this case, the solar-thermal, thermoelectric, electrothermal, and magnetothermal energy conversion of the phase-change composites were characterized along the parallel direction of the lamellar porous structure. A simulated solar illumination experiment was conducted on the phase-change composites with dimensions of 30 × 30 × 10 mm^3^ (length × width × height) to investigate their solar-thermal conversion performance using a xenon lamp with a light intensity of 1.0 kW m^−2^. As seen in temperature–time evolution curves (Figs. 4a and S29), both pure PW and its composites with the complex aerogels exhibit two temperature plateaus in the range of 38–45 °C during solar illumination and no irradiation, which are consistent with the *T*_m_ and *T*_c_ of PW (Table S4). This indicates that the temperature evolutions of pure PW and its composites are delayed by the thermal energy absorption and latent heat release resulting from the melting and crystallization of PW. After receiving continual solar irradiation for 960 s, pure PW shows a surface temperature of 44.6 °C. Meanwhile, the surface temperatures of the PI/KNF/GO-20@ZIF-67/PW, carbonized PI/KNF aerogel/PW, C@CoNC/PW, C/RGO-5@CoNC/PW, C/RGO-10@CoNC/PW, C/RGO-15@CoNC/PW, and C/RGO-20@CoNC/PW composites were found to rise up to 50.4, 54.3, 56.7, 63.6, 68.8, 74.5, and 78.6 °C, respectively. It is worth noting that the carbonized aerogel/PW composites exhibit much higher maximum surface temperature than pure PW and the PI/KNF/GO-20@ZIF-67/PW composite. This demonstrates that carbonization can enhance the solar-thermal conversion performance of the PI-based aerogels. The carbonized aerogel/PW composites all reveal a high-light absorptance of over 77.5% in the full spectrum range of 220–2500 nm (Fig. 4b). Moreover, a higher GO content promotes a higher-light absorptance of the carbonized aerogel/PW composites. As a result, the C/RGO-20@CoNC/PW obtained a high solar-thermal conversion efficiency of 95.1% as calculated by using Eq. S11. Such a result is evidently higher than the other aerogel/PW composites as well as pure PW (Table S5 and Fig. S30).

Figure 4c depicts the solar-thermal conversion and storage mechanisms of the C/RGO@CoNC/PW composite. As shown in Fig. 4c, the carbonaceous skeleton along with metallic Co can simultaneously generate the conjugation and LSPR effects on the C/RGO@CoNC complex aerogel under solar excitation, enabling the composite to obtain high-efficient sunlight absorption and therefore achieving high solar-thermal conversion efficiency. In particular, the neighboring *π*-electrons or interaction of *π*-bonds with *p*-orbital electrons are overlapped to allow electrons to leave their domains, resulting in the charge redistribution and generation of the conjugation effect [45]. The conjugation structures can provide high-efficient transport channels for photogenerated carriers to accelerate the mobility of holes and electrons. Consequently, the electrons in the carbonized aerogel can be easily excited, facilitating electronic transitions from the ground state to the higher energy orbital with a minimal energy input. With a return of the excited electrons to the ground state, a portion of energy is released as heat to cause a rise in macroscopic temperature by atomic lattice vibrations. A higher GO content can promote a more significant conjugation effect on the carbonized aerogel to make it obtain a better solar-thermal conversion capability [46]. In addition, when sunlight interacts with the surface of metallic Co, the free electrons on the Co surface undergo collective coherent oscillations with a photonic frequency matching their intrinsic frequency. The oscillations of free electrons can form electromagnetic waves to propagate along the Co surface to generate an electromagnetic field, consequently triggering the LSPR effect [47]. The LSPR effect enhances near-field interactions and generates hot electrons to promote solar-thermal conversion, causing a rise in the surface temperature of the C/RGO@CoNC/PW composite.

Figure 4d shows the temperature–time evolutions of the C/RGO-20@CoNC/PW composite as a representative sample under simulated solar illumination at different light intensities. After solar irradiation for 960 s, the composite shows an increase in its maximum surface temperature with increasing the light intensity, and its maximum temperature was determined to be 77.9, 92.0, 107.4, and 119.4 °C under irradiation intensities of 1.0, 2.0, 3.0, and 4.0 kW m^−2^, respectively. Moreover, the temperature plateau region becomes smaller as the light intensity increases. These results suggest that the C/RGO@CoNC/PW composite can gain acceleration in its solar-thermal energy conversion and storage under solar irradiation with high-light intensity. After solar irradiation at different light intensities, no distinct difference was observed in the temperature evolutions of the C/RGO-20@CoNC/PW composite during the following natural cooling process without solar irradiation. This is ascribed to the similar amounts of latent heat stored by the composite under solar irradiation at different intensities. During the cyclic process of solar illumination and no irradiation, the C/RGO-20@CoNC/PW composite exhibits a similar trend in its temperature evolutions with time (Fig. 4e). This indicates that the developed C/RGO@CoNC/PW composite gains a stable solar-thermal energy conversion/storage capability for long-term applications. An outdoor natural sunlight illumination experiment was conducted on the developed composite to investigate its solar-thermal energy conversion and storage performance in practical applications. As shown in Fig. 4f, a wooden house model was employed to be roof-covered with the PI/KNF/GO-20@ZIF-67 and C/RGO-20@CoNC aerogels and the PI/KNF/GO-20@ZIF-67/PW and C/RGO-20@CoNC/PW composites. Then, the house model was exposed to natural sunlight for 30 min and then moved to a shadow environment with an ambient temperature of 28 °C for natural cooling. As shown in the infrared thermal images (Fig. 4g), the two aerogel samples show a rapider change in their surface temperatures than the two aerogel/PW composites under natural sunlight illumination and no irradiation. There is no doubt that the complex aerogels have low thermal energy-storage capacities only dominated by sensible heat due to the absence of a PCM and therefore exhibit faster temperature evolutions under solar illumination and no irradiation. As observed from the temperature–time evolutions in Fig. 4h, the C/RGO-20@CoNC/PW composite presents the highest surface temperature of 67.5 °C among the four samples after natural sunlight illumination for 30 min. This can be attributed to its superior light absorption capability and thermal energy-storage capacity. Moreover, the C/RGO-20@CoNC/PW composite has a higher thermal conductivity than the PI/KNF/GO-20@ZIF-67/PW one and thus exhibits a faster solar-thermal energy-storage/release process under no solar irradiation. These results further confirm an excellent solar-thermal energy conversion/storage capability of the C/RGO@CoNC/PW composite for solar photothermal harvest and utilization.

### Solar Thermoelectric Generation

**Fig. 5:**
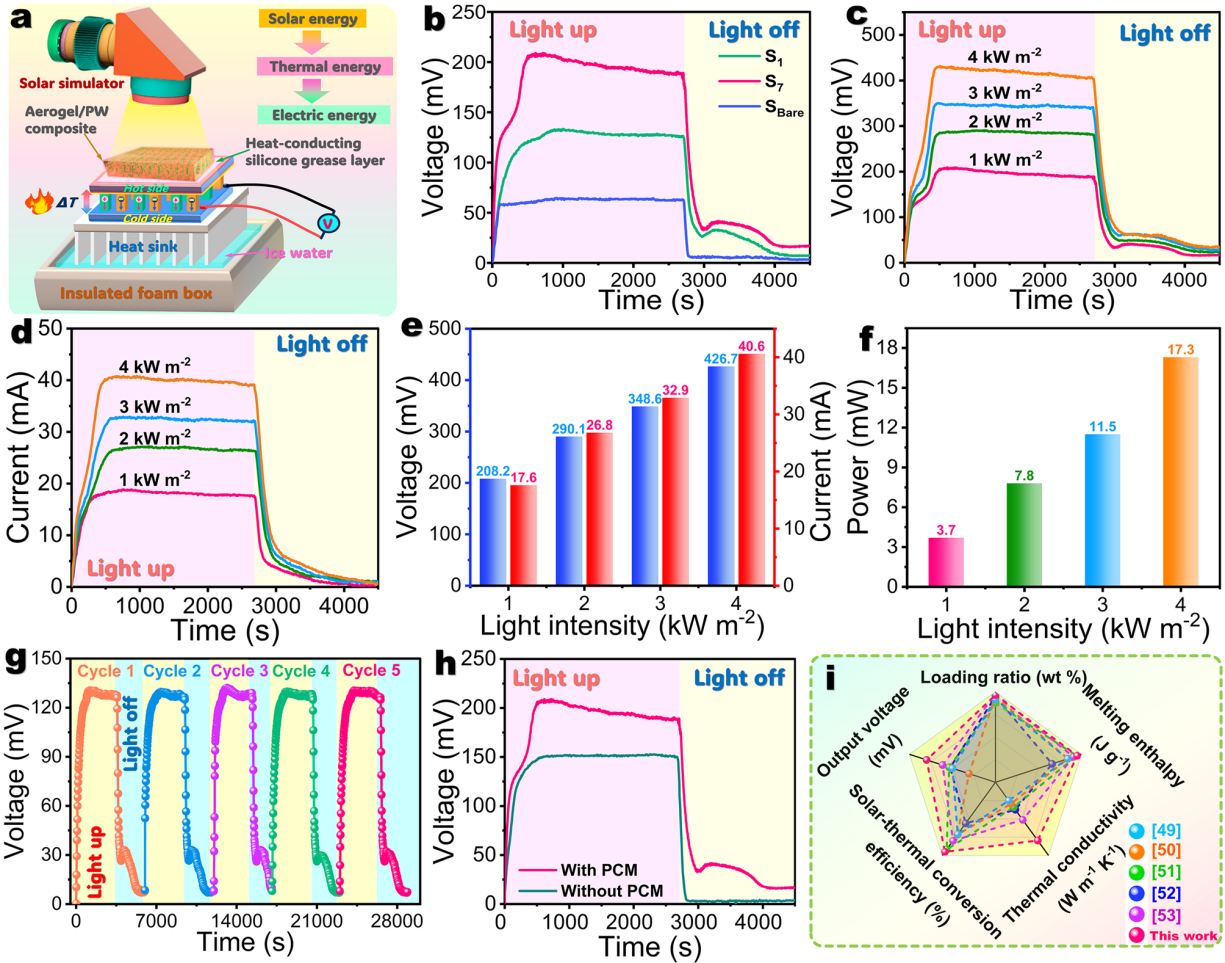
**a** Schematic solar-thermal-electric conversion system of STEG coupled with phase-change composites. **b** Output voltages of STEGs coupled with (S_1_) PI/KNF/GO-20@ZIF-67/PW and (S_7_) C/RGO-20@CoNC/PW composites and (S_Bare_) bare STEG under 1.0 kW m^−2^ solar irradiation. **c** Output voltages and **d** currents of STEG coupled with C/RGO-20@CoNC/PW composite at different light intensities. **e** Maximum output voltages and currents, and **f** output powers of STEG coupled with C/RGO-20@CoNC/PW composite at different light intensities. **g** Output voltages of STEG coupled with C/RGO-20@CoNC/PW composite during 5 cycles under 1.0 kW m^−2^ solar irradiation for 50 min and no irradiation for 34 min. **h** Output voltages of STEGs coupled with C/RGO-20@CoNC aerogel and C/RGO-20@CoNC/PW composites under 1.0 kW m^−2^ solar irradiation and natural cooling. **i** Comparison of comprehensive performance between the C/RGO-20@CoNC/PW composite developed in this study and counterparts reported in the literature

With such a distinguished solar-thermal energy conversion/storage capability, the C/RGO@CoNC/PW composite is expected to be used for solar thermoelectric generation. For this purpose, a solar thermoelectric generator (STEG) was assembled with a thermoelectric module coupled with the C/RGO-20@CoNC/PW composite as a representative sample to verify its solar thermoelectric generation performance (Fig. 5a). The thermoelectric module is laminated with the composite on its hot side, and its cold side is contacted with ice water. The composite can act as both a solar absorber and a thermal energy-storage material to absorb and store solar photothermal energy as sensible and latent heat to keep a high temperature on the hot side. Meanwhile, ice water can provide a low temperature for the cold side. In this case, a stable vertical temperature gradient can be formed between the cold and hot sides of the thermoelectric module to generate a stable potential difference on the basis of the Seebeck effect [48]. Figure 5b shows the output voltages of a bare STEG without a PCM and the STEGs coupled with the PI/KNF/GO-20@ZIF-67/PW and C/RGO-20@CoNC/PW composites under 1.0 kW m^–2^ simulated solar irradiation and no irradiation. The maximum output voltage of the bare STEG was determined to be 62.4 mV. In contrast, the STEGs coupled with the PI/KNF/GO-20@ZIF-67/PW and C/RGO-20@CoNC/PW composites obtain much higher maximum output voltages of 126.4 and 188.2 mV, respectively. Owing to the superior solar-thermal conversion capability of the C/RGO-20@CoNC/PW composite, the corresponding STEG can absorb a larger amount of solar photothermal energy and convert it into heat compared to the bare STEG and STEG coupled with the PI/KNF/GO-20@ZIF-67/PW composite. Therefore, the absorbed heat can generate a larger temperature difference than the other two STEGs to generate a higher output voltage. The output voltage of the bare STEG was found to drop quickly to zero under no solar irradiation due to a sharp decrease in the temperature of its hot side. However, the STEGs coupled with the two phase-change composites were found to still maintain certain output voltages for 1300 s under no solar irradiation. This can be attributed to a higher temperature on the hot side due to sensible and latent heat release by the PW loaded in the two composites.

The STEG coupled with the C/RGO-20@CoNC/PW composite was found to exhibit a similar variation trend in its output voltage and current under simulated solar illumination at different light intensities (Fig. 5c, d). Its output voltage and current were found to increase with an increase in the light intensity. The STEG obtained an output voltage of 208.2 mV and an output current 17.6 mA under 1.0 kW m^–2^ solar irradiation (Fig. 5e). Under 4.0 kW m^–2^ solar irradiation, its output voltage and current rise up to 426.7 and 40.6 mA, respectively. There is no doubt that a higher-light intensity can promote a higher output power. As a result, the STEG obtained output powers of 3.7, 7.8, 11.5, and 17.3 mW under 1.0, 2.0, 3.0, and 4.0 kW m^–2^ solar irradiation, respectively (Fig. 5f). These results suggest that a higher-light intensity can result in a higher temperature on the hot side of the STEG and thus generate a higher output power. A cyclic solar irradiation–no irradiation experiment was conducted on the STEG coupled with the C/RGO-20@CoNC/PW composite to examine its power output stability. The STEG exhibits a stable maximum output voltage during the cyclic process (Fig. 5g), indicating good cyclic stability for solar thermoelectric power output. Moreover, to assess the positive influence of the loaded PW on the solar thermoelectric generation of the C/RGO@CoNC/PW composite, we also investigated the power output of the STEG coupled with the C/RGO-20@CoNC aerogel under 1.0 kW m^–2^ solar irradiation. It is worth noting that the STEG coupled with the C/RGO-20@CoNC aerogel exhibits an output voltage of 133.1 mV, which is lower than that of the STEG coupled with the C/RGO-20@CoNC/PW composite by 27.6% under identical irradiation conditions (Fig. 5h). Owing to the introduction of PW as a PCM, the developed composite can utilize both sensible and latent heat harvested from solar photothermal energy to make the hot side of the STEG keep a higher temperature under solar irradiation, resulting in a higher output voltage. The stored latent heat can be released under no irradiation to maintain a higher temperature on the hot side of the STEG. Therefore, the STEG coupled with the C/RGO-20@CoNC/PW composite generates a higher output voltage than that coupled with the C/RGO-20@CoNC aerogel. These results indicated that the PW loaded within the developed composite can effectively improve the output voltage of the STEG through sensible and latent heat storage and release. As seen in Fig. 5i, the C/RGO-20@CoNC/PW composite exhibits much better comprehensive performance in thermal energy-storage capacity, solar-thermal conversion efficiency, thermal conductance, and solar thermoelectric power output than other similar functional composites reported in the literature [49–53]. Having such distinguished comprehensive performance, the developed composite exhibits great potential for the solar-thermal-electric energy conversion application.

### Electrothermal Energy Conversion and Storage

**Fig. 6:**
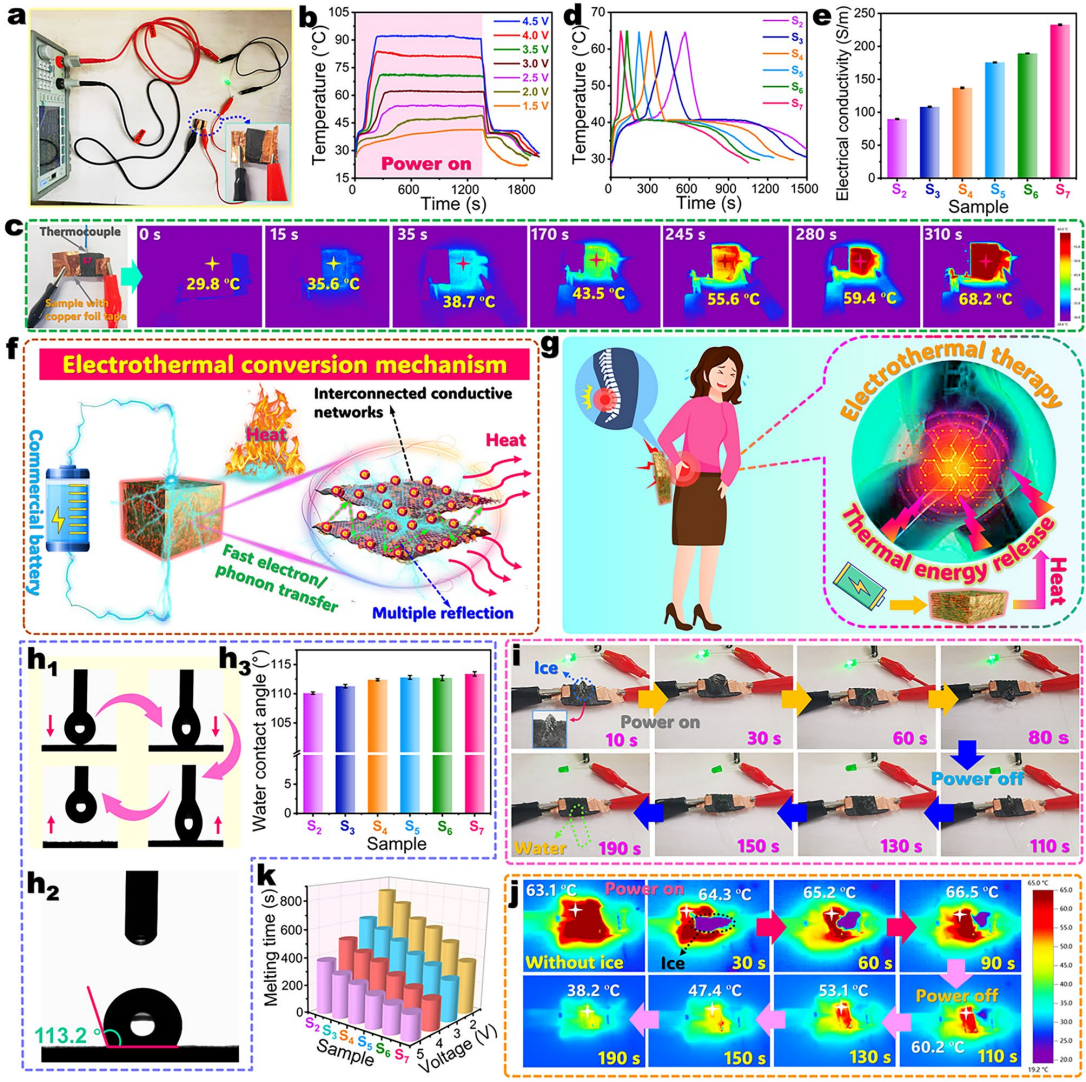
**a** Experimental setup for measuring the conductivity of carbonized aerogel/PW composites. **b** Temperature–time evolutions of C/RGO-20@CoNC/PW composite at different input voltages. **c** Thermal infrared images of C/RGO-20@CoNC/PW composite at an input voltage of 4.0 V. **d** Temperature–time evolutions of (S_2_) carbonized PI/KNF aerogel/PW, (S_3_) C@CoNC/PW, (S_4_) C/RGO-5@CoNC/PW, (S_5_) C/RGO-10@CoNC/PW, (S_6_) C/RGO-15@CoNC/PW, and (S_7_) C/RGO-20@CoNC/PW composites during electrothermal energy conversion at input voltages of 4.0 V. **e** Electrical conductivities of carbonized aerogel/PW composite samples. **f** Schematic electrothermal conversion mechanism of carbonized aerogel/PW composites. **g** Scheme of an electrically triggered carbonized aerogel/PW composite for thermal therapy. Optical images of **h**_**1**_ a water droplet contacting, depressing, and detaching from the surface of C/RGO-20@CoNC/PW composite and **h**_**2**_ water contact angle of C/RGO-20@CoNC/PW composite. **h**_**3**_ Water contact angles of carbonized aerogel/PW composite samples. **i** Digital photographs and **j** Infrared thermographic images of C/RGO-20@CoNC/PW composite during the deicing process at input voltages of 4.0 V for 80 s and 0 V for 110 s. **k** Total melting time of a block of ice (0.5 × 1.0 × 0.4 cm^3^) on different carbonized aerogel/PW composite samples after energization at different voltages for 180 s

Solar-thermal energy conversion is highly dependent on the solar irradiation intensity, and the intermittent sunlight illumination may restrict the application of PCMs and their composites in some special fields such as high-efficient thermal therapy and deicing. Compared to solar-thermal energy conversion, the electrothermal conversion based on the Joule’s law can overcome the limitation of intermittent sunlight illumination. Although PW as an organic PCM has a low conductivity of around 10^−13^ S m^–1^ and cannot be used for electrothermal conversion and storage, the C/RGO@CoNC aerogel has an excellent charge transport ability to provide effective conductive paths to transfer electrical energy for electrothermal energy conversion. As for the C/RGO@CoNC/PW composite, the Joule heat generated by electrothermal conversion not only can be immediately used as sensible heat, but also can be stored by the loaded PW as latent heat for future use as needed. The electrothermal conversion performance of the C/RGO@CoNC/PW composite was investigated by using a custom-designed setup comprising a copper electrode and a CNC-controlled high-precision power supply (Fig. 6a). When the developed composite was connected with the setup, a light-emitting diode (LED) light was lighted up, indicating the presence of a conductive path for electrothermal conversion. The *I*–*V* curve of the C/RGO-20@CoNC/PW composite as a representative sample exhibits a high fitness level of 99.5% at different input voltages (Fig. S31), proving that the current characteristics of the composite conform to the Ohm’s law. According to the temperature–time evolutions of the C/RGO-20@CoNC/PW composite at different input voltages (Fig. 6b), the composite obtained a maximum temperature in a short time, and then, the temperature could be maintained stably at 39.5, 48.8, 54.3, 62.0, 70.3, 80.7, and 90.8 °C under input voltages of 1.5, 2.0, 2.5, 3.0, 3.5, 4.0, and 4.5 V, respectively. This result demonstrates that the C/RGO@CoNC/PW composite can be heated to a desired temperature in a short time, and its temperature can be controlled by adjusting the input voltage, which is suitable for human thermotherapy. For example, the composite can complete a full phase transition within 50 s at 4.5 V to reach a comfortable temperature of 41 °C for thermotherapy of the human body. There is a good linear relationship between the temperature and square output voltage (*U*^2^) (Fig. S32), demonstrating that the actual temperature of the composite is in good agreement with its theoretical one. This result further confirms that the C/RGO@CoNC/PW composite can act as a reliable electric component with the current characteristics conforming not only to the Ohm’s law but also to the Joule’s law (*Q* = *U*^2^ *t R*^–1^) [54].

The electrothermal energy conversion performance of carbonized aerogel/PW composites was characterized by electrical energization at an input voltage of 4.5 V. The C/RGO-20@CoNC/PW composite as a representative sample shows a rapid rise in the surface temperature with time as indicated by its infrared thermal images (Fig. 6c). Its surface temperature was found to reach 68.2 °C after energization for 310 s. This result is consistent with that obtained from the measurement using a thermocouple. All of the carbonized aerogel/PW composites exhibit a fast increase in temperature during the energizing process (Fig. 6d). It takes 75, 125, 213, 306, 422, and 567 s for the carbonized PI/KNF aerogel/PW, C@CoNC/PW, C/RGO-5@CoNC/PW, C/RGO-10@CoNC/PW, C/RGO-15@CoNC/PW, and C/RGO-20@CoNC/PW composites, respectively, to reach a maximum temperature of 65 °C after energization. The C/RGO-20@CoNC/PW composite exhibits the shortest energization time among these carbonized aerogel/PW composites due to its highest electrical conductivity contributed by its highest GO content. As seen in Fig. 6e, the C/RGO-20@CoNC/PW composite has an electrical conductivity of 232.8 S m^−1^, which is 159.5% higher than that of the carbonized PI/KNF aerogel/PW composite. It is evident that a higher electrical conductivity promotes a faster electrical heating process of the composite. The carbonized aerogel skeleton in the C/RGO@CoNC/PW composite can act as a unique three-dimensional interconnected conductive network to offer a continuous path for rapid electron and phonon transportation (Fig. 6f). Moreover, the presence of highly graphitized RGO nanosheets can generate a free *p*-electron conjugation effect on the carbonized aerogel skeleton to facilitate the coupling of electrons and phonons, enhancing the electrical and thermal conduction of the composite. As a result, the composite obtained a reduction in its electrical resistance to generate a greater current. The carbonized aerogel/PW composites also show a temperature plateau during the energizing and de-energizing processes due to the latent heat storage and release, respectively, by their loaded PW (Fig. 6d). In addition, these composites were found to present much longer periods for latent heat release than that for Joule heat storage, realizing long-term heat release within a short Joule heat storage period. These results demonstrate that the C/RGO@CoNC/PW composite can be activated by a commercial battery for heat management and thermal therapy of the human body. As schematically depicted in Fig. 6g, the C/RGO@CoNC/PW composite can be heated to a temperature higher than 40 °C by using a portable battery within a short period, and the energized composites can maintain a comfortable temperature range over an extended heat release period. In this case, the developed C/RGO@CoNC/PW composite can be used to wrap around a hip joint for effective electricity-driven thermal therapy.

In addition to the application for thermal therapy, the C/RGO@CoNC/PW composite can also be used for anti-icing and deicing. The loaded PW component has an inherently hydrophobic property to prevent against the accumulation of water and suppress the ice formation on the surface of the composite. A highly conductive skeleton can drive electrothermal energy conversion at a low input voltage to obtain a high surface temperature for the C/RGO@CoNC/PW composite within a short time for deicing. This is beneficial to the application of the C/RGO@CoNC/PW composite in ice-prone environments. As seen in Fig. 6h_1_, a water droplet can easily be detached from the surface of the C/RGO-20@CoNC/PW composite, indicating its high hydrophobicity. In particular, the C/RGO-20@CoNC/PW composite shows a water contact angle of 113.2° (Fig. 6h_2_). On the other hand, the water contact angle of the composite only increases slightly with an increase in the GO content (Fig. 6h_3_). It is understandable that the introduction of CoNC and RGO as nano-sized particles can improve the surface roughness of the composites. A rougher surface can trap more air and reduce the contact area between the water droplet and solid surface according to the Wenzel model, resulting in a larger contact angle of the composites [55]. Moreover, the presence of CoNC nanoparticles increases the surface heterogeneity to reduce the wettability of the composites. As a result, the composite obtained high hydrophobicity. An electrothermal deicing experiment was conducted to examine the practical deicing performance of the C/RGO@CoNC/PW composite by placing a block of ice on its surface at an input voltage of 4.0 V. The ice was found to melt completely after energizing for 80 s and then de-energizing for 110 s (Fig. 6i). According to the thermal infrared images in Fig. 6j, the surface temperature of the composite increased rapidly during the energizing process, which is advantageous to the melting of the ice. The PW loaded within the composite can absorb thermal energy during the energizing process and release it after stopping energization. For this case, the composite can maintain a high surface temperature during the de-energizing process through latent heat release by PW to promote the continuous melting of the ice, generating high-efficient deicing effectiveness. It should be noted that the ice on the composite shows a reduction in the melting time with an increase in the RGO content (Fig. 6k). This can be explained by the fact that a higher RGO content enables the C/RGO@CoNC/PW composite to obtain higher electric conduction and thus results in a higher surface temperature during the energizing process. This result is consistent with the electrothermal conversion performance of the carbonized complex aerogel/PW composites (Fig. 6d). As a result, the C/RGO-20@CoNC/PW composite presents the shortest time for the melting of the ice among the carbonized aerogel/PW composites (Fig. 6k). Moreover, a higher input voltage results in a shorter melting time of the ice, confirming that the deicing process can be accelerated by applying high input voltage for the carbonized aerogel/PW composites. These results demonstrate that the developed C/RGO@CoNC/PW composite possesses superior hydrophobicity and an excellent electrothermal conversion capability for anti-icing and deicing applications.

### Magnetothermal Energy Conversion and Storage

**Fig. 7:**
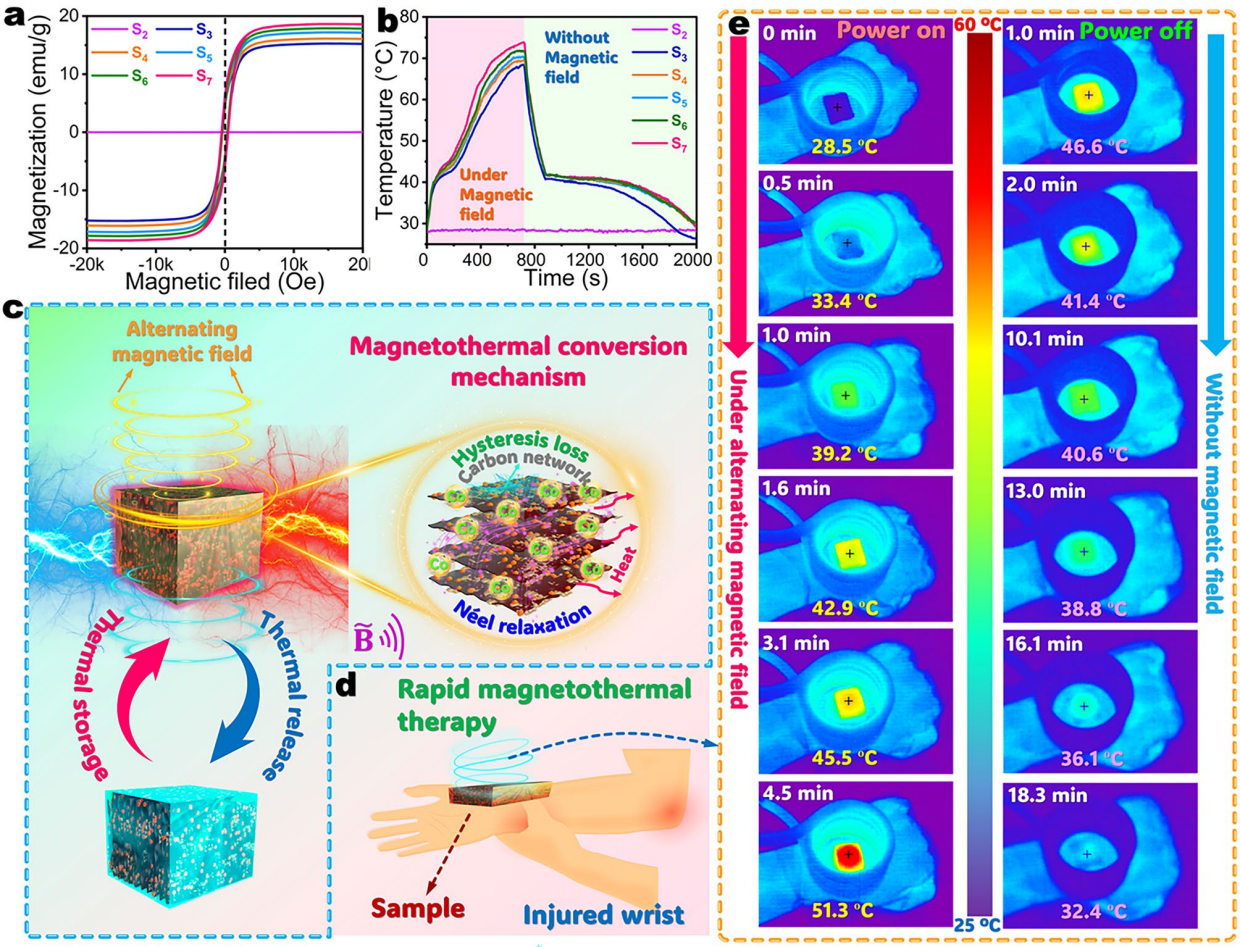
**a** Magnetic hysteresis loops and **b** temperature–time evolutions of (S_2_) carbonized PI/KNF aerogel/PW, (S_3_) C@CoNC/PW, (S_4_) C/RGO-5@CoNC/PW, (S_5_) C/RGO-10@CoNC/PW, (S_6_) C/RGO-15@CoNC/PW, and (S_7_) C/RGO-20@CoNC/PW composites. **c** Schematic mechanism of magnetothermal conversion and latent heat storage of C/RGO@CoNC/PW composite. **d** Scheme of thermal therapy for a wrist by the magnetically triggered C/RGO@CoNC/PW composite. **e** Infrared thermal images of a wrist covered with C/RGO-20@CoNC/PW composite under the applied alternating magnetic field (1.05 MHz) and without a magnetic field

Magnetothermal energy conversion can offer precise temperature control by applying an alternating magnetic field and therefore is considered as a promising alternative method for advanced thermal management systems, emergency heating, and human thermal therapy. It is well known that metallic Co has a strong magnetic response. The presence of Co enables the C/RGO@CoNC/PW composite to achieve paramagnetic characteristics for magnetothermal energy conversion and storage. There is no magnetic behavior observed from the carbonized PI/KNF aerogel/PW composite due to the absence of Co (Fig. 7a). Therefore, the carbonized PI/KNF aerogel/PW only maintains a low temperature of 28.2 °C under an alternating magnetic field (10 A, 1.05 MHz) as seen in Fig. 7b. In contrast, the carbonized Co-containing aerogel/PW composites exhibit a hysteresis loop with very low magnetic retention and coercivity under the alternating magnetic field. The C@CoNC/PW, C/RGO-5@CoNC/PW, C/RGO-10@CoNC/PW, C/RGO-15@CoNC/PW, and C/RGO-20@CoNC/PW composites were found to show saturation magnetization of 15.25, 16.11, 17.18, 17.87, and 18.61 emu g^−1^, respectively. These composites show a rapid rise in their temperatures when being exposed to the alternating magnetic field. A higher temperature was achieved for the composite with a higher RGO content under the applied magnetic field for 720 s. Such magnetothermal conversion effectiveness can be explained by the hysteresis loss and Néel relaxation as depicted in Fig. 7c. The Co component is magnetized under an alternating magnetic field, compelling its local magnetic moments to rotate irreversibly along the direction of the applied magnetic field. This process leads to a partial loss of magnetic energy by heat dissipation due to the hysteresis effect. Meanwhile, the Néel relaxation takes placed in the magnetized Co component, in which the magnetic moments align with the external magnetic field without any physical rotations. With the oscillation of the magnetic field, the magnetic moments are adjusted to reach a new equilibrium and generate heat. Therefore, the magnetized Co component in the C/RGO@CoNC/PW composite can respond to an alternating magnetic field to convert magnetic energy into heat energy and store it as sensible and latent heat by the composites. As a type of conductive additive with a π-conjugated structure, RGO nanosheets can interact with CoNC nanoparticles on their interfaces to promote the magnetic coupling between the CoNC nanoparticles. This increases the local magnetic anisotropy of the CoNC nanoparticles and results in the rearrangement of magnetic moments, thus improving the magnetization strength of the developed C/RGO@CoNC/PW composite [56, 57]. It is worth noting that the C/RGO-20@CoNC/PW composite exhibits the highest saturation magnetization among all of the carbonized aerogel/PW composites due its highest RGO content, achieving the highest temperature under the applied magnetic field.

With a high-efficient magnetothermal conversion capability, the C/RGO@CoNC/PW composite can be applied for thermal therapy of the human body. As illustrated in Fig. 7d, an injured wrist was covered by the C/RGO-20@CoNC/PW composite as a representative sample, and then, the magnetothermal conversion was conducted under an alternating magnetic field to heat the covered area for thermal therapy. Figure 7e shows the thermal infrared images of a human wrist covered with the composite under an alternating magnetic field and without a magnetic field. The composite obtained a high temperature of over 50 °C within 4.5 min under the applied magnetic field at a frequency of 1.05 MHz, enabling the composite to carry out thermal therapy for the wrist. After deactivating the applied magnetic field, the composite still maintains a comfortable temperature of above 38 °C for 13 min for continuous thermal therapy due to the latent heat release by the loaded PW. This result demonstrates that the developed C/RGO@CoNC/PW composite has great application potential in human thermal therapy through its magnetothermal energy conversion and latent heat storage.

### EMI Shielding Performance

**Fig. 8:**
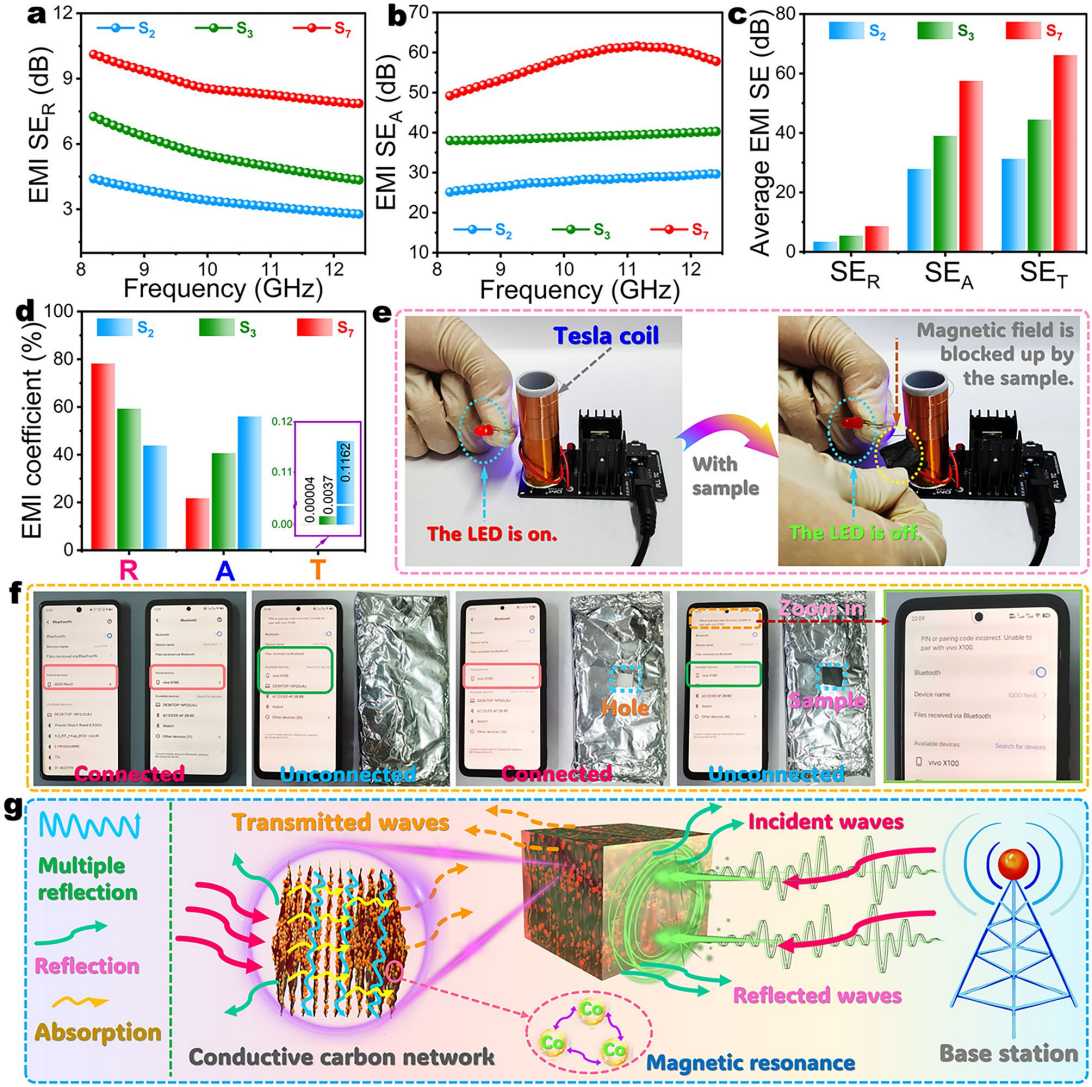
**a** EMI SE_R_, **b** SE_A_, **c** average SE values and **d** EMI coefficients of (S_2_) carbonized PI/KNF aerogel/PW, (S_3_) C@CoNC/PW, and (S_7_) C/RGO-20@CoNC/PW composites. **e** Digital photographs of EMI shielding experiment for C/RGO-20@CoNC/PW composite via a Tesla coil. **f** Digital images of EMI shielding experiment of C/RGO-20@CoNC/PW composite for the Bluetooth connection between two cell phones. **g** Schematic EMI shielding mechanism of C/RGO@CoNC/PW composite

The C/RGO@CoNC/PW composite has a highly conductive aerogel skeleton, which not only can perform effective solar-thermal-electric, electrothermal, and magnetothermal energy conversion, but also can generate an EMI shielding effect to prevent against electromagnetic waves. To explore the EMI shielding effectiveness (SE) of the C/RGO@CoNC/PW composite, the reflection SE (SE_R_) and absorption SE (SE_A_) were analyzed in the *X*-band range (8.2–12.4 GHz) using the C/RGO-20@CoNC/PW composite as a representative sample and the carbonized PI/KNF aerogel/PW and C@CoNC/PW as references, and the resultant results are presented in Fig. 8a–c. Owing to the presence of CoNC as a magnetic substance, the C@CoNC/PW and C/RGO-20@CoNC/PW composites show an increase in the average SE_R_ and SE_A_ values by 61% and 40%, respectively, compared to the carbonized PI/KNF aerogel/PW one (Fig. 8c). There is no doubt that the polarization and hysteresis losses generated by metallic Co can enhance the EMI shielding performance of the composites through boosting their electromagnetic wave absorption to minimize the secondary contamination from multiple reflections [58, 59]. Moreover, the introduction of RGO effectively increases the mobile charge carriers in the carbonized aerogel skeleton to make it achieve a higher electrical conductivity as a result of higher SE_R_ and SE_A_ values [60]. In this case, the carbonized PI/KNF aerogel/PW, C@CoNC/PW, and C/RGO-20@CoNC/PW composites obtained total SE (SE_T_) of 31.3, 44.5, and 66.2 dB, respectively. Figure 8d shows the reflection coefficient (R), absorption coefficient (A), and transmission coefficient (T) of the three composite samples. The sum of the R and A values was found to be nearly close to 100%. This results in extremely low T values of the three composites, indicating their outstanding EMI shielding performance. The R values of the composites show an increase with the introduction of CoNC nanoparticles and RGO nanosheets. The PI/KNF aerogel/PW, C@CoNC/PW, and C/RGO-20@CoNC/PW composites exhibit R values of 43.9%, 59.3%, and 78.2%, respectively. A high R value for the C/RGO-20@CoNC/PW composite suggests that reflection domains EMI shielding. As a result, the C@CoNC/PW and C/RGO-20@CoNC/PW composites show a decrease in the A value with the incorporation of CoNC nanoparticles and RGO nanosheets. The A value of the C/RGO-20@CoNC/PW composite was determined to be 21.8%, indicating that absorption dissipates the part of incident EMI energy. The C/RGO-20@CoNC/PW composite shows a high R value due to the good electrical conductivity contributed by CoNC and RGO components. A high RGO content offers a stronger significant reflection effect by its well-developed conductive network, effectively blocking incident electromagnetic waves at the surface of the composite and significantly reducing their penetration accordingly. Based on the results mentioned above, a synergistic combination of reflection and absorption enables the C/RGO-20@CoNC/PW composite to gain an excellent shielding capability.

To examine the functional compatibility of the C/RGO-20@CoNC/PW composite under high-temperature environments, we investigated its EMI SE at elevated temperatures, and the obtained results are shown in Fig. S33. There is a slight improvement in both the SE_R_ and SE_A_ values of the composite at high temperatures compared to those at room temperature. PW is in a solid state with high crystallinity at room temperature and therefore has low polarization with a low dielectric constant. This results in negligible electromagnetic wave absorption. When the temperature is higher than the *T*_m_ of PW, PW is melted into liquid, and it presents disordered molecular arrangement and increased charge distribution, leading to higher polarization. As a result, its dielectric constant is increased and its absorption loss is enhanced. Additionally, the liquid PW enhances the fluidity of the composite, which may alter the propagation path of electromagnetic waves within the material to increase multiple reflection losses. The results indicated that the C/RGO-20@CoNC/PW composite can maintain its superior EMI performance at elevated temperatures.

Two practical application experiments were conducted to further evaluate the EMI shielding performance of the developed C/RGO@CoNC/PW composite. In the first experiment, a Tesla coil was assembled to establish an electromagnetic field surrounding the coil (Fig. 8e). This experimental procedure is clearly displayed by Video S1. As seen in Video S1, an electromagnetic field is generated to form an electromotive force to light a light-emitting diode (LED) when switching on the power. However, the LED goes off when placing a piece of the developed composite between the coil and LED. This is because the presence of the composite blocks up the electromagnetic field, causing the disappearance of electromotive force, followed by power cut. This phenomenon demonstrates an effective EMI shielding behavior resulting from the developed composite. In the second experiment, the EMI shielding performance of the developed composite was assessed by employing two cell phones. As shown in Fig. 8f, a wireless commination is successfully established between the two cell phones through the Bluetooth connection. When one cell phone is enwrapped by a tinfoil, the established communication is terminated due to the electromagnetic wave blocking by the tinfoil. The two cell phones were found to reconnect with each other when opening a hole on the tinfoil. However, the Bluetooth connection between the two cell phones is suspended again when covering the hole with the developed composite. These phenomena demonstrates that the developed composite can provide effective EMI shielding for electronic devices in addition to multi-energy conversion and storage. Figure 8g depicts the EMI shielding mechanism of the developed composite. When incident electromagnetic waves encounter the composite, a part of them are reflected from its surface. The residual electromagnetic waves can penetrate into the composite to interact with its carbonized aerogel skeleton. Owing to the high electrical conductivity and large surface area of the carbonized aerogel skeleton, the multiple reflection and absorption of the residual electromagnetic waves take place in the composite to diminish their intensity. Moreover, the carbonized aerogel skeleton can amplify the overall reflective shielding of the composite thanks to its unique multi-layer structure. The co-existence of RGO and CoNC can generate a synergistic interaction to optimize the reflection and absorption of electromagnetic waves by the carbonized aerogel skeleton. On the one hand, the introduction of RGO not only further enhances the electrical conductivity of the carbonized aerogel skeleton to establish an effective pathway for the electromagnetic wave reflections, but also is advantageous to the electromagnetic wave absorption of the composite through converting electromagnetic energy into heat by polarization and eddy current. On the other hand, the presence of CoNC promotes the electromagnetic wave absorption by hysteresis losses, mitigating the secondary reflections. These comprehensive effects boost the EMI shielding performance of the developed C/RGO@CoNC/PW composite significantly.

Table S6 summarizes the thermal stability, solar-thermal conversion efficiency, EMI shielding effectiveness of the developed C/RGO@CoNC/PW composite and the other aerogel-based PCM composites reported in the literature. It is worth noting that the performance of the C/RGO@CoNC/PW composite is superior to most of those aerogel-based PCM composites, suggesting that the developed composite can act as a state-of-the-art material for effective solar-thermal conversion and EMI shielding. For example, it can be used in 5G base stations or electronic devices under harsh conditions. The exceptional electromagnetic wave shielding capabilities of the developed composite enable the 5G base stations or electronic devices to prevent electromagnetic interference and ensure a stable operation. Moreover, the developed composite can protect the 5G base stations or electronic devices from exposing to extreme heat or cold environments. Specifically, the Joule heating effect allows the developed composite as a low-voltage electric heater to heat the stations or devices in cold environments. In high-temperature conditions, the PW component in the composite can prevent the thermal transformation from the environment to the stations or devices through thermal regulation by phase changes. With these distinguished characteristics, the developed composite offers superior electromagnetic shielding efficiency, better energy conversion capabilities, and lower energy consumption than traditional metal-based electromagnetic shielding materials and advanced state-of-the-art materials to satisfy its practical applications in different harsh environments. Based on the production costs listed in Table S7, the production cost of the C/RGO-20@CoNC composite (30 × 30 × 30 mm^3^) is 1.88 USD after the complex fabrication process. To meet the requirement of large-scale applications, low-cost industrial raw materials are suggested to be used as alternatives, and the used solvents can be recycled for producing the C/RGO-20@CoNC composite. In addition, the fabrication process of the C/RGO-20@CoNC composite needs to be simplified to improve the production efficiency.

## Conclusions

In summary, a type of multifunctional carbonized PI-based aerogel/PW phase-change composite was successfully developed for solar-thermal, thermoelectric, electrothermal, and magnetothermal multi-energy conversion/storage and electromagnetic shielding applications. Such a novel phase-change composite was fabricated with the C/RGO@CoNC aerogel as a supporting material and PW as a PCM. The C/RGO@CoNC aerogel was derived from high-temperature carbonization of the PI/KNF/GO@ZIF-67 complex aerogel, resulting in a unique bidirectional lamellar porous structure with high porosity and ultra-lightweight characteristic. The presence of RGO and CoNC in the aerogel skeleton enhances the thermal conductivity, sunlight absorption, electrical conductivity, and magnetic properties of the developed composite significantly. These distinguished characteristics contribute to a solar-thermal conversion efficiency of 95.1%, a water contact angle of over 110°, an electrical conductivity of 232.8 S m^−1^, saturation magnetization of 18.61 emu g^−1^, and EMI SE of 66.2 dB in the *X*-band for the developed composite. The introduction of PW enables the developed composite to obtain a high latent heat capacity of 209.2 J g^–1^, enhancing its thermal energy-storage capability for practical applications. In this sense, the developed composites can be applied for multi-energy conversion along with effective EMI shielding for multipurpose applications, such as solar-thermal, electrothermal conversion, and magnetothermal conversion and energy storage for solar energy harvest and utilization, deicing, and human thermal therapy. A thermoelectric system based on the developed composite can generate maximum output voltage and current of 426.7 and 40.6 mA, respectively, for efficient thermoelectric conversion. In addition, the developed composite shows a good EMI shielding capability to prevent against electromagnetic contamination for electronic devices. Through a rational integration of prominent multi-energy conversion/storage function and excellent EMI shielding performance, this study provides some innovative insights for development of advanced functional materials with great potential for multipurpose applications in various fields.

## Supplementary Information

Below is the link to the electronic supplementary material.

Supplementary file1 (DOCX 20974 KB)

Supplementary file2 (MP4 2014 KB)
