# Supplementary material for: Multi-Energy Conversion and Electromagnetic Shielding Enabled by Carbonized Polyimide/Kevlar/Graphene Oxide@ZIF-67 Bidirectional Complex Aerogel-Encapsulated Phase-Change Materials
Source: Nanomicro Lett. 2025 Apr 27;17:236. doi: 10.1007/s40820-025-01761-w (PMC12034614; doi:10.1007/s40820-025-01761-w)
Supplement: Supplementary file 1 — Supplementary file1 (DOCX 20974 KB) [file 40820_2025_1761_MOESM1_ESM.docx]

Supplementary Information

Multi-Energy Conversion and Electromagnetic Shielding Enabled by Carbonized Polyimide/Kevlar/Graphene Oxide@ZIF-67 Bidirectional Complex Aerogel-Encapsulated Phase Change Materials

Tao Shi^1^, Xing Gao^1^, Huan Liu^1^*, and Xiaodong Wang^1^*

^1^State Key Laboratory of Organic–Inorganic Composites, Beijing University of Chemical Technology, Beijing 100029, P. R. China

*Corresponding authors. E-mail: [liu.huan@mail.buct.edu.cn](mailto:liu.huan@mail.buct.edu.cn) (Huan Liu); [wangxd@mail.buct.edu.cn](mailto:wangxd@mail.buct.edu.cn) (Xiaodong Wang)

**Section S1 Characterizations and Measurements**

The morphologies of KNFs, GO nanosheets, aerogels, and phase-change composites were characterized by using a Zeiss SUPRA™ 55 scanning electron microscope at an acceleration voltage of 20 kV. The microstructure of KNFs, GO nanosheets and C/RGO@CoNC aerogel were observed by using a FEI Talos F200S high-resolution transmission electron microscope. Energy-dispersive *X*-ray (EDX) spectra together with elemental mapping images were recorded on an Oxford INCAX-Act EDX spectrometer to analyze surface elemental distributions. The Raman spectra of aerogel samples were obtained on a Horiba LabRAM HR Evolution Raman microscope equipped with an argon ion laser under an excitation wavelength of 532 nm. The *X*-ray powder diffraction patterns of KNFs, GO nanosheets, RGO nanosheets, aerogel samples, and phase-change composite samples were recorded on a Japan Rigaku Rigaku SmartLab SE *X*-ray diffractometer using a Cu–Kα radiation source. The Fourier-transform infrared spectra of KNFs, GO nanosheets, RGO nanosheets, aerogels, and phase-change composites were recorded on a Thermal Scientific Nicolet iS5 infrared spectrometer in the wavelength range of 400–4000 cm^–1^ at a resolution of 2 cm^–1^. X–ray photoelectron spectroscopy was conducted by using a Thermo Scientific K-Alpha XPS spectrometer equipped with a focused monochromatized Al-Kα radiation source. The pore size, density and volume of aerogel samples were characterized by using a Micromeritics AutoPore V 9620 mercury intrusion porosimeter in a pressure range of 0.5–62354.60 Psia. The crystallization behavior of PW and C/RGO@CoNC/PW composites were observed on an Olympus BX51 optical microscope equipped with a Sony CCD-IRIS digital camera using the TSView-c software.

Dynamic differential scanning calorimetry (DSC) scans were performed by using a TA Instruments Q20 differential scanning calorimeter equipped with a thermal analysis data station to analyze the phase-change behaviors of pure PW and phase-change composite samples. The measurement was carried out at a heating/cooling rate of 10 °C/min under a nitrogen atmosphere, and the mass of each specimen was about 3–6 mg. The phase-change characteristic data were directly obtained from the DSC thermograms. The *w* (loading ratio, %), *η* (enthalpy efficiency, %), and *λ* (relative enthalpy efficiency, %) were determined by using the following equations.

$w(\%)=\frac{m_{composite}-m_{aerogel}}{m_{composite}}\times100\%$ (S1)

$\eta(\%)=\frac{\Delta H_{m(composite)}}{\Delta H_{m(PW)}}\times100\%$ (S2)

$\lambda(\%)=\frac{\Delta H_{m(composite)}+\Delta H_{c(composite)}}{w\times(\Delta H_{m(\text{PW)}}+\Delta H_{c(PW)})}\times100\%$ (S3)

where *m*_composite_, *and m*_aerogel_ are the masses of phase-change composite and aerogel, respectively, Δ*H*_m(PW)_ and Δ*H*_m(composite)_ are the melting enthalpies of pure PW and phase-change composite, respectively, and Δ*H*_c(PW)_ and Δ*H*_c(composite)_ are the crystallization enthalpies of pure PW and phase-change composite, respectively. The thermal cycling stability of phase-change composites was also determined by the DSC. The shape stability of pure PW and phase-change composites was determined through a heat impact experiment. The samples were placed on a high-precision electronic heating platform at 100 °C, and their aspects were recorded by a digital camera. The thermal conductivity of pure PW and phase-change composites were measured by using a Netzsch LFA467 laser thermal conductivity instrument at 25 °C. Each sample was tested three times to calculate its average value. Thermal conductivity was calculated by using the equation of *κ* =*α* × *C*_p_ ×*ρ*, where *α* is the thermal diffusivity, *C*_p_ is the specific heat capacity, and *ρ* is the density.

The ultraviolet (UV)-visible-infrared absorption spectra of phase-change composites were recorded on a Shimadzu UV-3600 Plus UV-visible spectrophotometer. The photothermal energy conversion and storage performance of phase-change composites were evaluated by using a custom-designed experimental setup equipped with a Xenon arc lamp as simulated sunlight. The Xenon arc lamp was equipped with a standard AM1.5 G optical filter. The light irradiation intensity was measured by using a SM206 plus optical power meter. The surface temperature evolution of phase-change composites with time was recorded by means of a *k*-type thermocouple under simulated solar illumination and natural cooling. To evaluate the long-term photothermal stability of the C/RGO@CoNC/PW composite, a 40-cycle photothermal experiment was carried out, and the surface temperature evolution was recorded every 5 cycles. In order to evaluate the solar-thermal energy conversion and energy storage performance in practical applications, the PI/KNF/GO aerogel, C/RGO@CoNC aerogel, PI/KNF/GO/PW and C/RGO@CoNC/PW composites were placed on the top of a simulated wooden house, and then the house was exposed to natural sunlight for 0.5 h and transferred to a shady place for natural cooling. The temperature-time evolutions of the aerogels and phase-change composites were monitored by using a Testo™ 875-1i infrared thermographic camera. A thermoelectric generation module was assembled by using a Guangzhou Sirui electronic SR 1206 difference thermoelectric module and a heat sink. The underside of the phase change composite was bonded to the hot side of the thermoelectric generation module by a thermally conductive silver silicone grease. The cold side of the thermoelectric generation module was bonded to the heat sink by using a silver silicone grease. The temperature-time evolution of phase-change composites during solar-thermal-electrical conversion was recorded by using a data logger equipped with a *k*-type thermocouple. The output voltage and current were recorded by a Youlide UT61D+ digital multimeter.

Water contact angles were measured by using a JC2000DM POWEREACH water contact angle measuring instrument. The output power source used in this work was a DC power supply with a switching mode (MS-305DS, Shenzhen Pioneer Instrument Technology Co. Ltd., China). In the Joule heating test, the samples were fixed at both ends with a conductive copper tape without touching and then connected to a DC power supply. The temperature-time evolution inside the phase-change composites was recorded by a data logger equipped with the *k*-type thermocouple as the DC power supply turned on and off. The surface temperature-time evolution was recorded by the infrared thermographic camera. An electrothermal deicing experiment was carried out at room temperature to confirm the anti-deicing ability of phase change composite. The composite loaded with a block of ice on its surface was irradiated by the Xenon arc lamp (1.0 kW m^–2^) as a simulated solar source. The passive deicing process was observed and monitored by the infrared thermographic camera.

The magnetic properties of phase-change composites were characterized by a Lakeshore 7404 vibrating sample magnetometer. The magnetothermal energy conversion and storage performance of phase-change composites were determined by an IGBT induction heating equipment. Specifically, A wrist covered with the C/RGO-20@CoNC/PW composite was placed under a customized induction coil and exposed to an alternating magnetic field. The surface temperature evolution of C/RGO-20@CoNC/PW composite within the alternating magnetic field was recorded by the thermal infrared camera. The electrical conductivity was measured using a four-probe tester (RTS-8, Guangzhou Four-Point Probe Technology Co. Ltd., China). Each specimen was tested for five times at different surface locations to obtain an average result. The electromagnetic interference shielding effectiveness (SE) was measured by a waveguide method using a vector network analyzer (VNA, Agilent PNA N5244A) in the frequency range of X-band (8.2–12.4 GHz). The as-prepared samples were cut into the desired sizes of 22.86 mm × 10.16 mm. More than three samples were performed for each component under the same testing condition. There are three shielding mechanisms that contribute to the total SE (SE_T_), including the reflection SE (SE_R_), absorption SE (SE_A_), and multireflection SE (SE_MR_). According to the Schoellkopf theory, when the SE_A_ is higher than 10 dB, SE_T_ is determined by the sum of SE_A_ and SE_R_. The total SE_T_, SE_R_, and SE_A_ can be calculated by using the S-parameter as follows:

$R=\left|S_{11}\right|^{2}$ (S4)

$T=\left|S_{12}\right|^{2}$ (S5)

$T+R+A=1$ (S5)

$SE_{T}=-10 \text{log(}\left|S_{12}\right|^{2}\text{) = }-\text{10 log(}\left|S_{12}\right|^{2}\text{)}$ (S6)

$SE_{R}=-10 \text{log(1}-\left|S_{11}\right|^{2}\text{) }$ (S7)

$SE_{A}=-10 \text{log}\left(\frac{\left|S_{12}\right|^{2}}{1-\left|S_{11}\right|^{2}}\right)=SE_{T}-SE_{R}$ (S8)

where R, A, and T are the reflection, absorption, and transmitting losses, respectively.

**Section S2 Finite Element Simulation for Thermal Conduction**

The thermal conduction of the C/RGO@CoNC aerogel and C/RGO@CoNC aerogel/PW composite perpendicular and parallel to channels was simulated by the finite element software COMSOL Multiphysics. The solid heat transfer physics module was utilized to simulate the process of surface temperature variation over time. A geometric model was constructed with the solid material contained of a multilayer structure and an irregular concave-convex shape. The rest part of the solid material was the PW component. A transient solution method was performed for the simulation model with the same loading sub-steps of 11 steps and total time of 10 min. In the simulation analysis, the ambient temperature was set to 25 °C. The top and side surfaces of the solid material were used as the heating surfaces. The heating temperature was set to 120 °C. The convective heat flux on the solid material surfaces, heat sources, and heat flux boundary conditions was considered. The solid heat transfer in the aerogel and phase change composite was governed by the thermal conduction equations as follows:

$\rho C_{p}\mathbf{u}\cdot\nabla T+\nabla\cdot\mathbf{q}=Q+Q_{ted}$ (S9)

$\mathbf{q}=-k\nabla T$ (S10)

where *ρ* represents density (kg m^–3^), *C_p_* represents specific heat capacity at constant pressure (J Kg^–1^ K^–1^), **u** represents velocity vector (m s^–1^), **q** represents heat flux per unit area per unit time (W m^–2^), *Q* represents the heat source term (W m^–3^), *Q_ted_* represents the heat source correction term (W m^–3^), and *k* represents the thermal conductivity (W m^–1^ K^–1^). Through simulating the thermal conduction on different planes, the thermal behavior of the layered solid structure was demonstrated, and the impact of heating conduction at different surfaces was analyzed.

**Supplementary Figures**

**
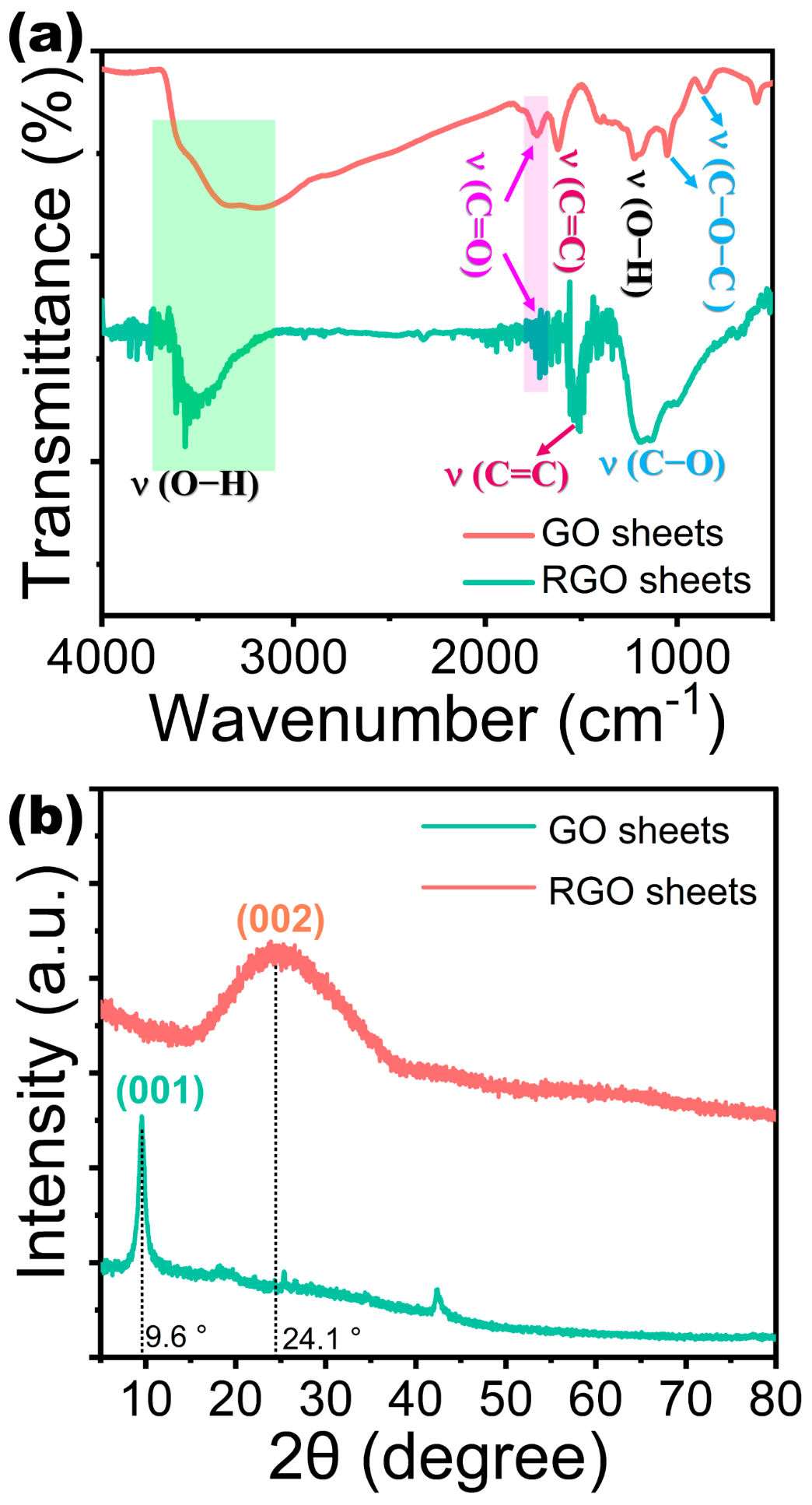

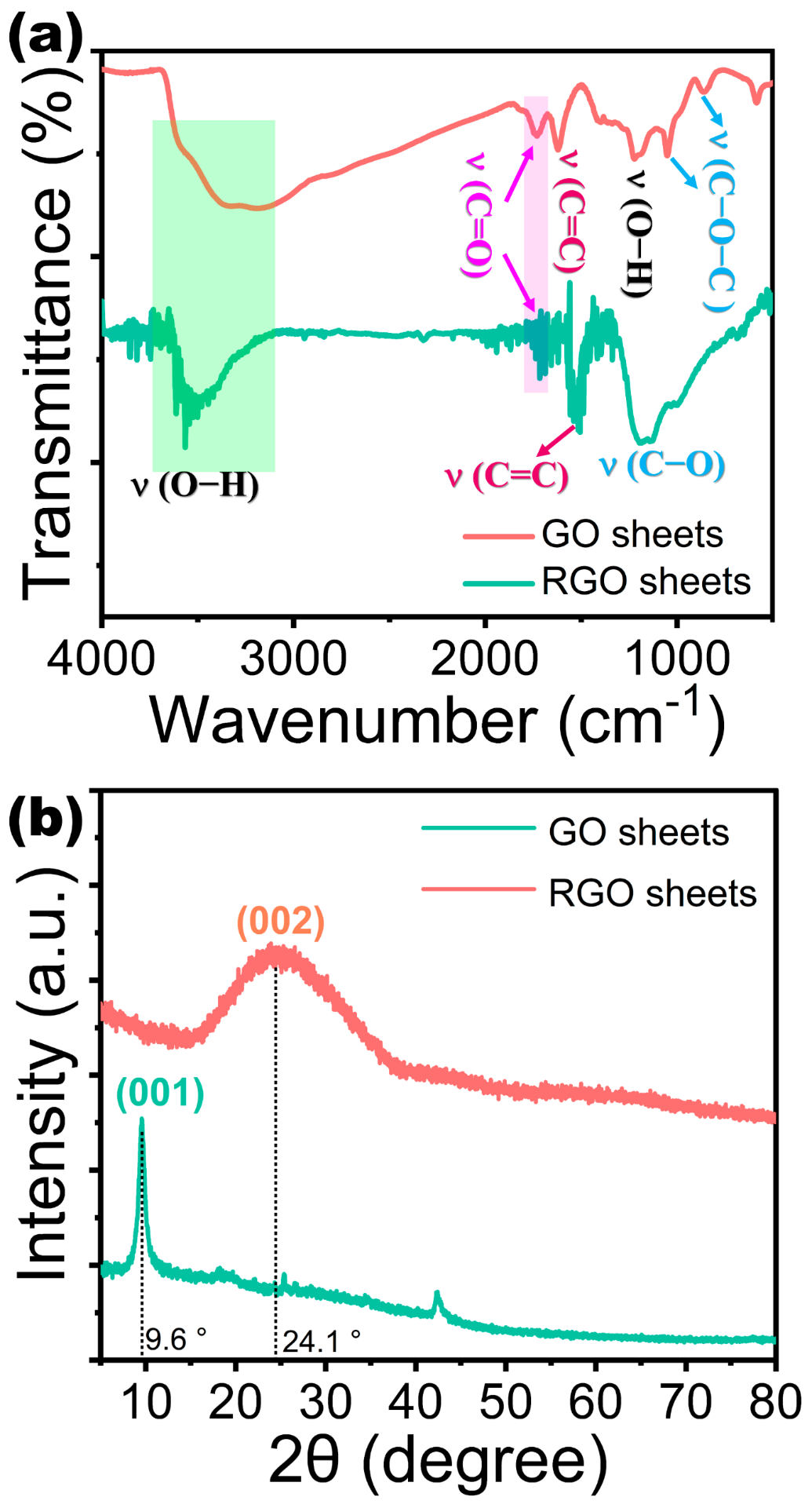
**

**Fig. S1** **a** FTIR spectra and **b** XRD patterns of GO and RGO nanosheets


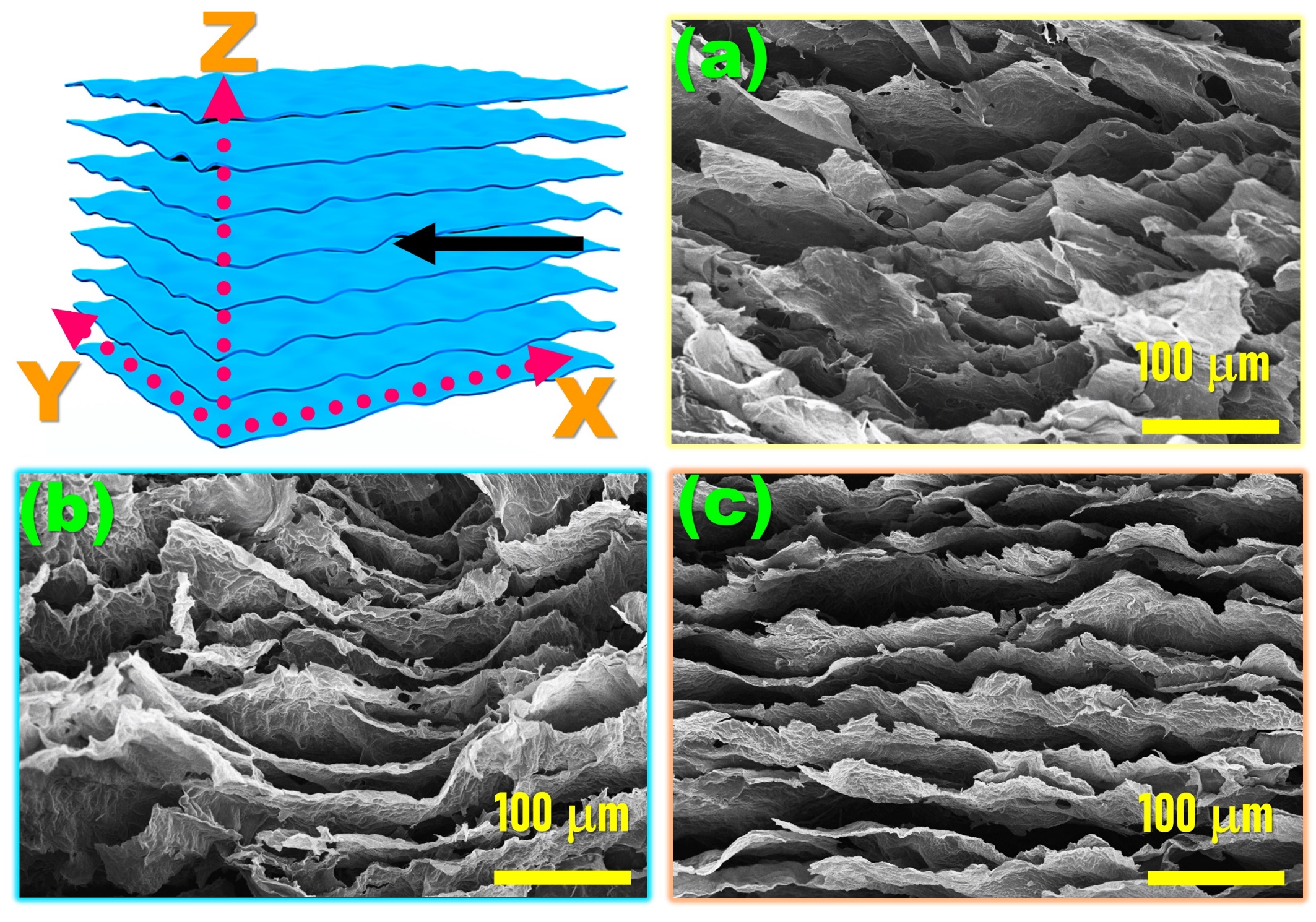
 **Fig. S2** SEM micrographs of **a** PI/KNF/GO-20, **b** PI/KNF/GO-20@ZIF-67, and **c** C/RGO-20@CoNC aerogels in the *X–Z* plane


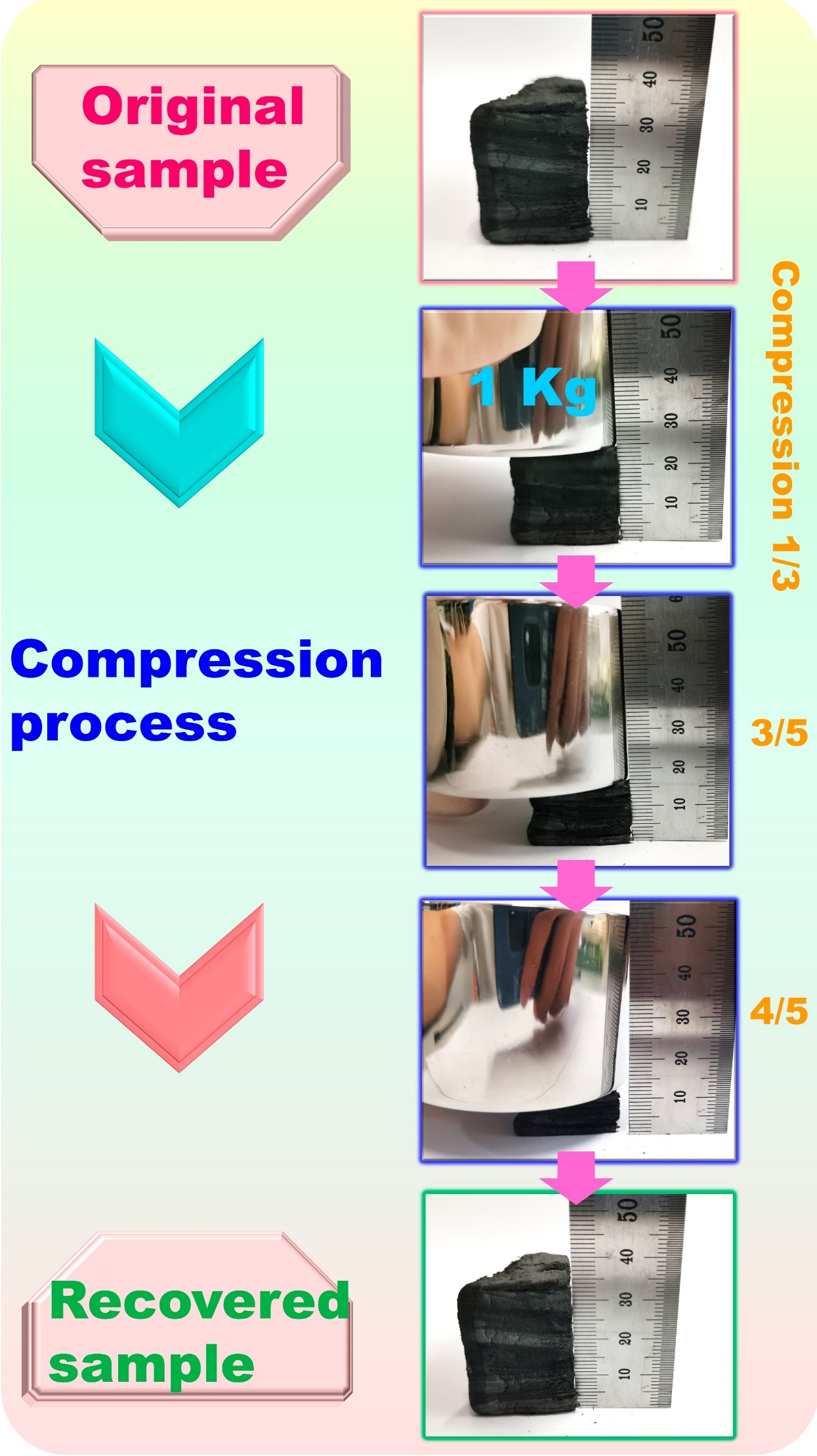


**Fig. S3** Digital photographs of PI/KNF/GO-20 aerogel under the compressive test with 1Kg weight


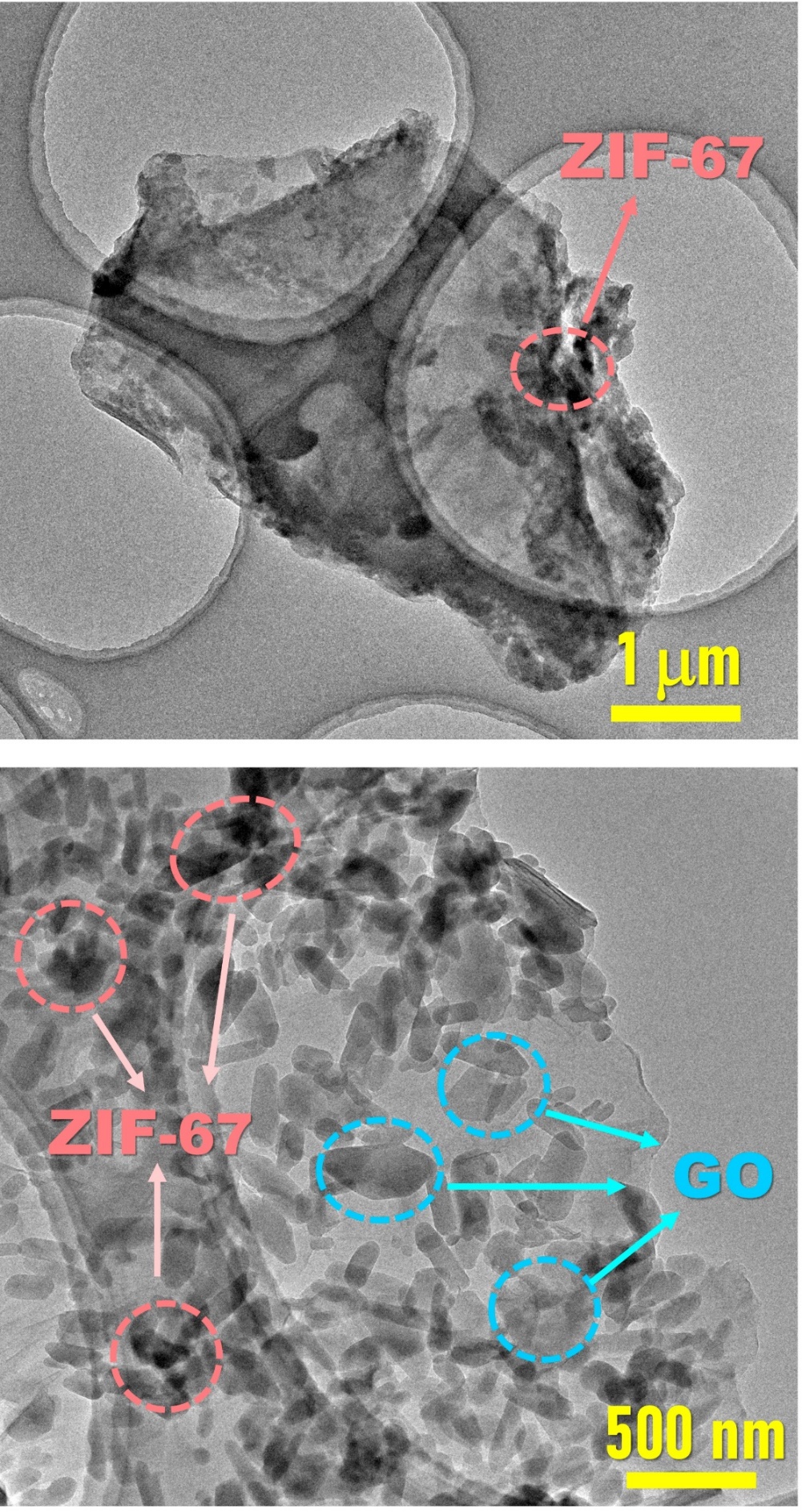

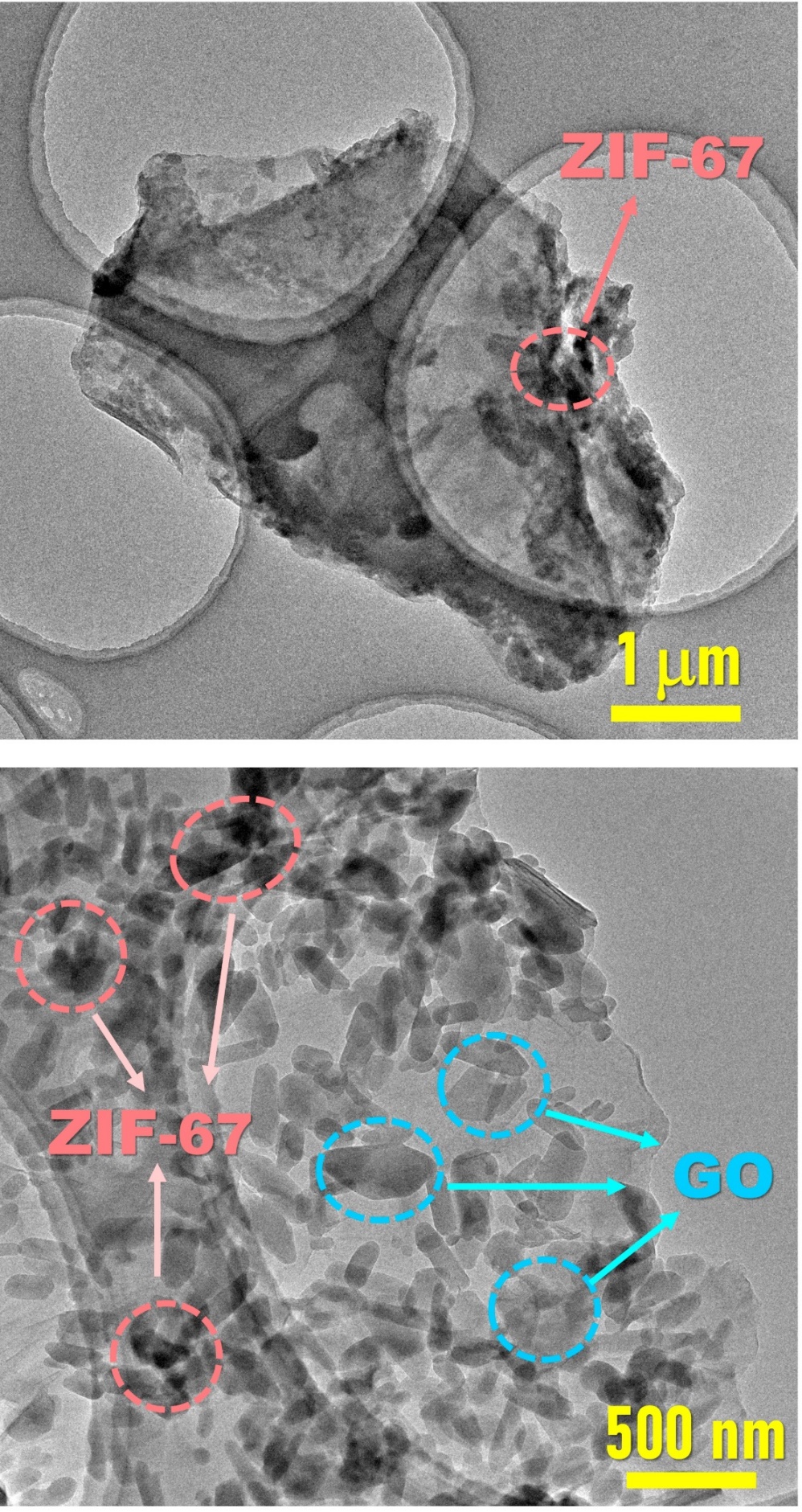


**Fig. S4** TEM images at different magnifications of PI/KNF/GO-20 aerogel

**
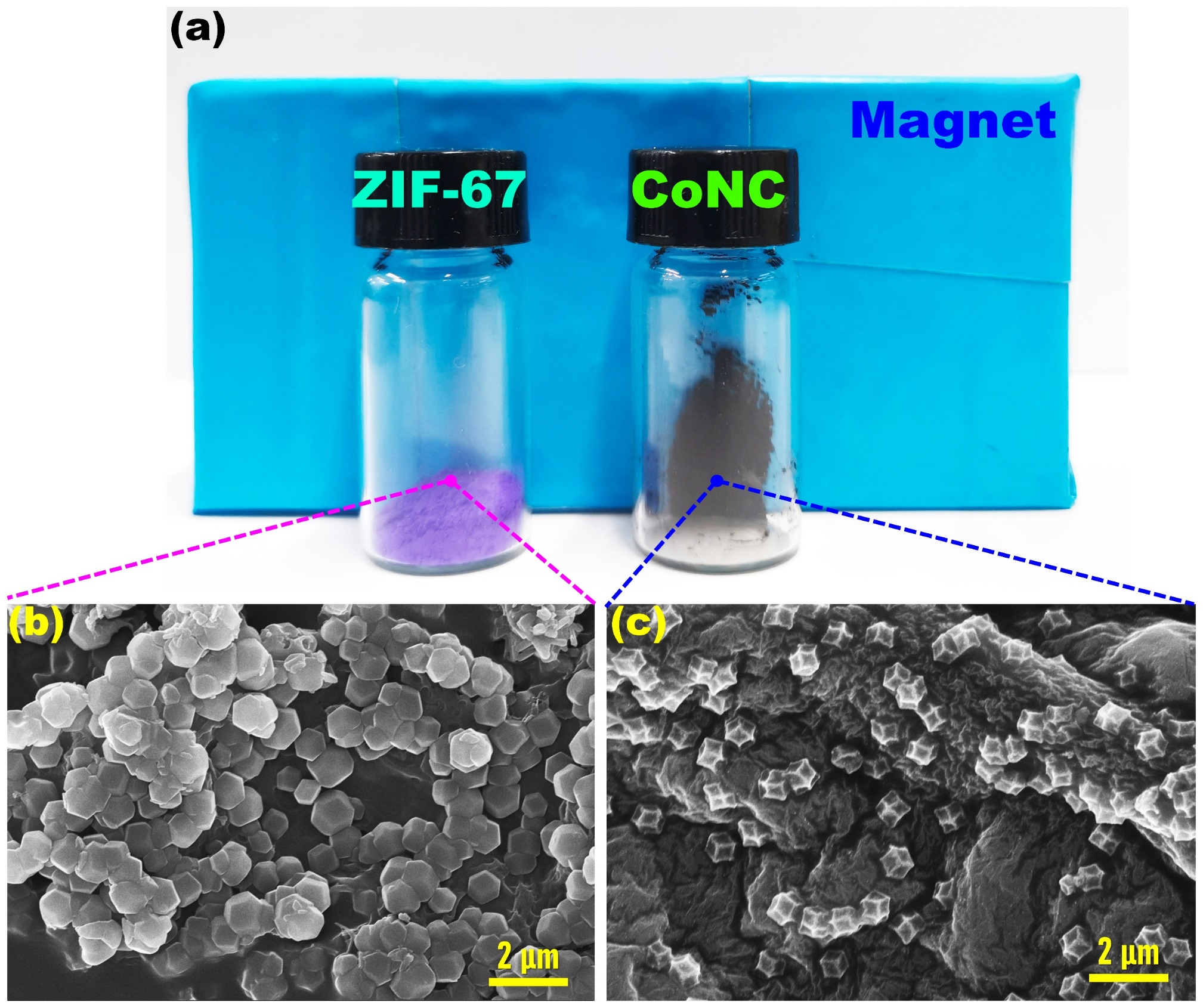
**

**Fig. S5 a** Digital photographs of ZIF-67 powder (Left) and CoNC powder (Right). SEM images of **b** ZIF-67 and **c** CoNC nanoparticles

**
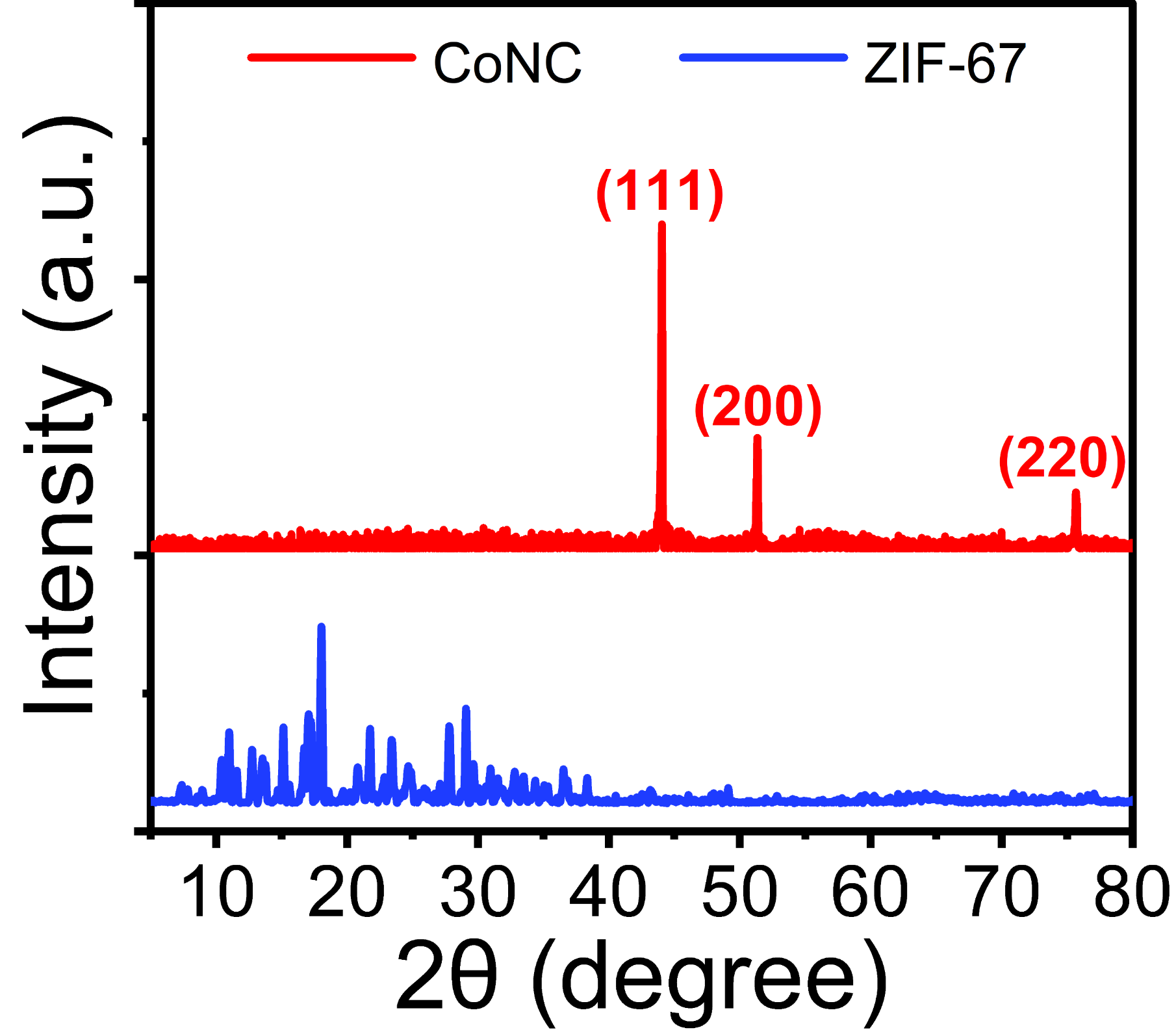
**

**Fig. S6** XRD patterns of ZIF-67 and CoNC nanoparticles


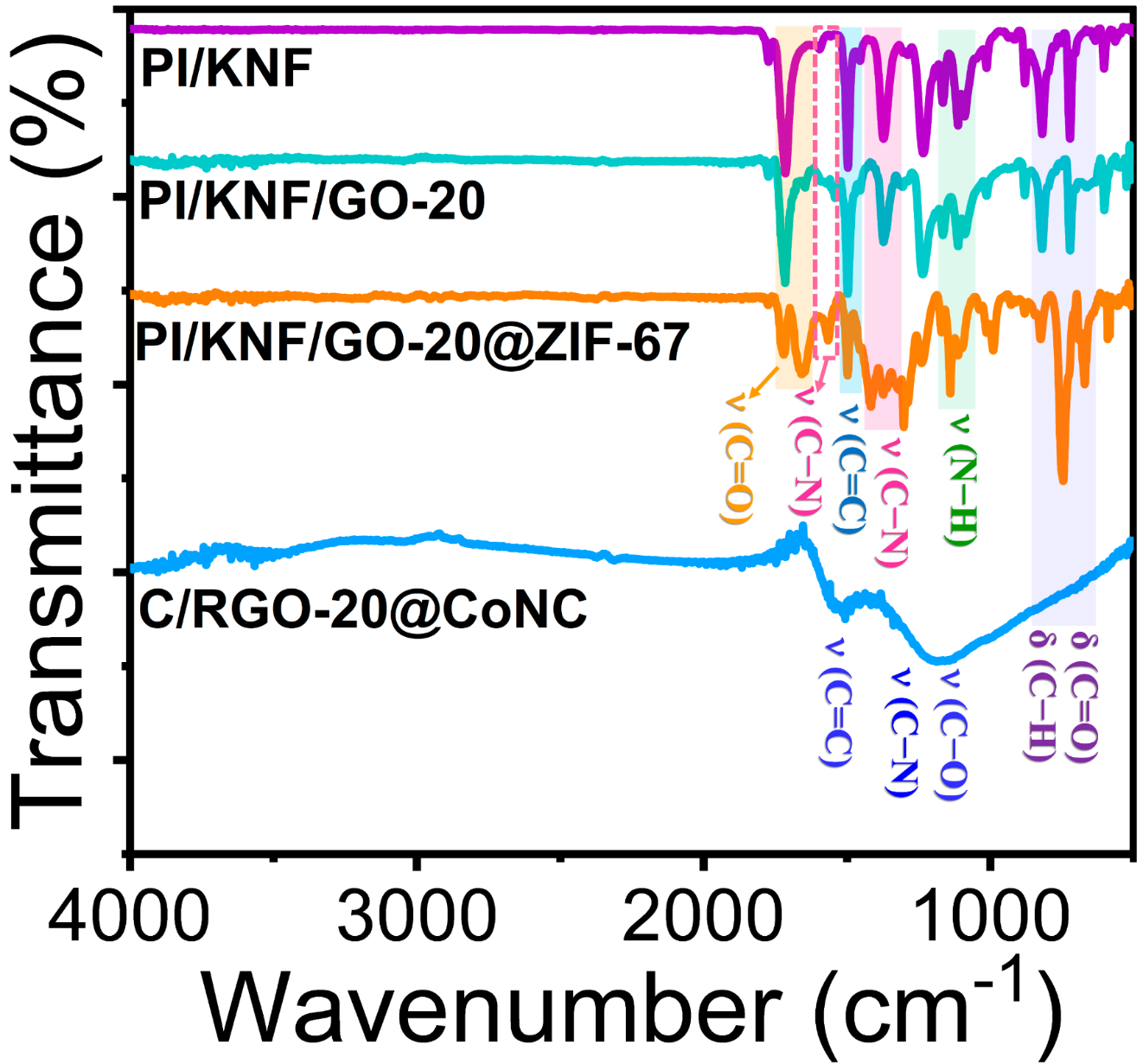


**Fig. S7** FTIR spectra of PI/KNF, PI/KNF/GO-20, PI/KNF/GO-20@ZIF-67 and C/RGO-20@CoNC aerogels

**
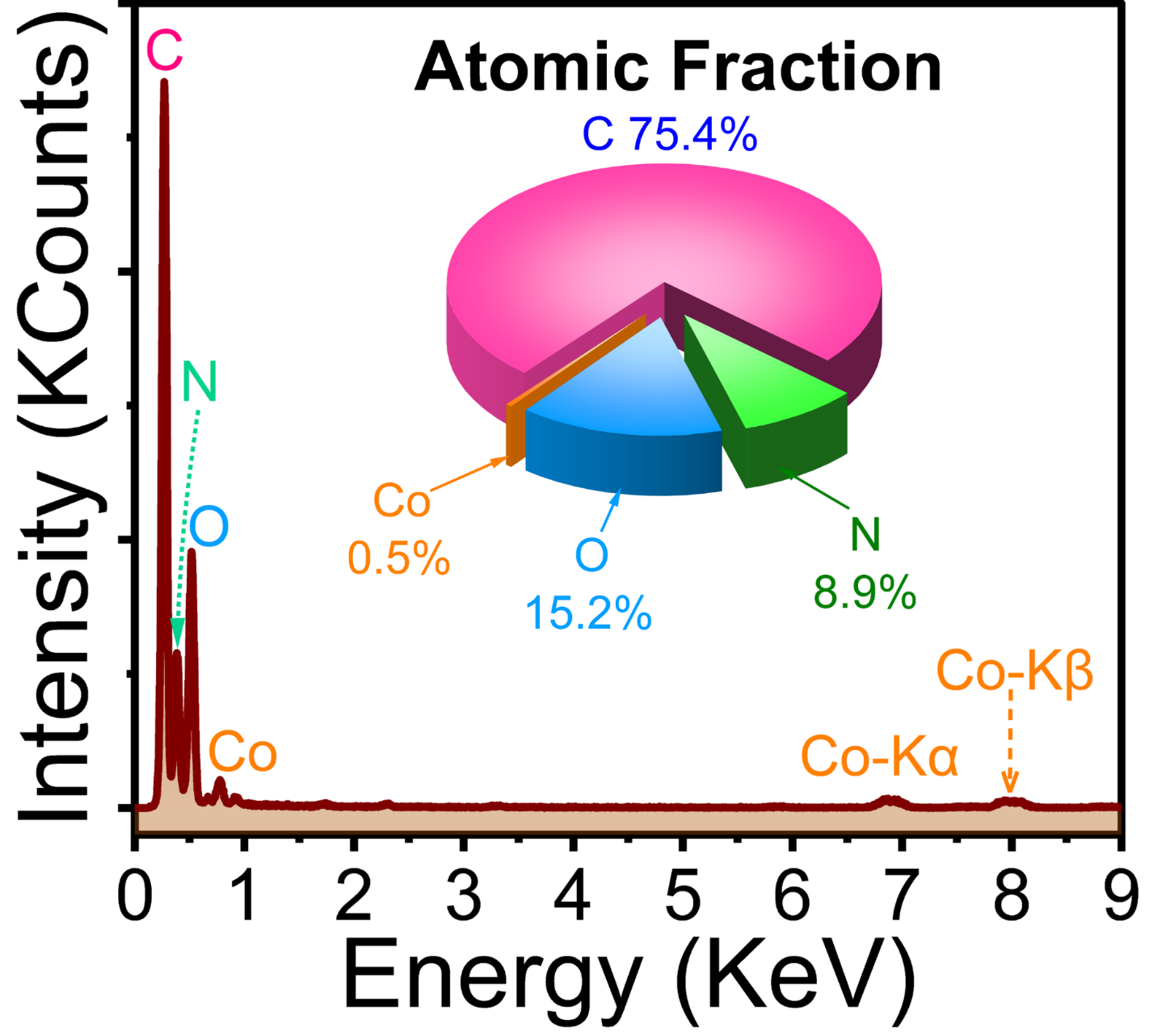
**

**Fig. S8** EDS spectra and atomic fraction of PI/KNF/GO-20@ZIF-67 aerogel obtained in High-resolution TEM mode

**
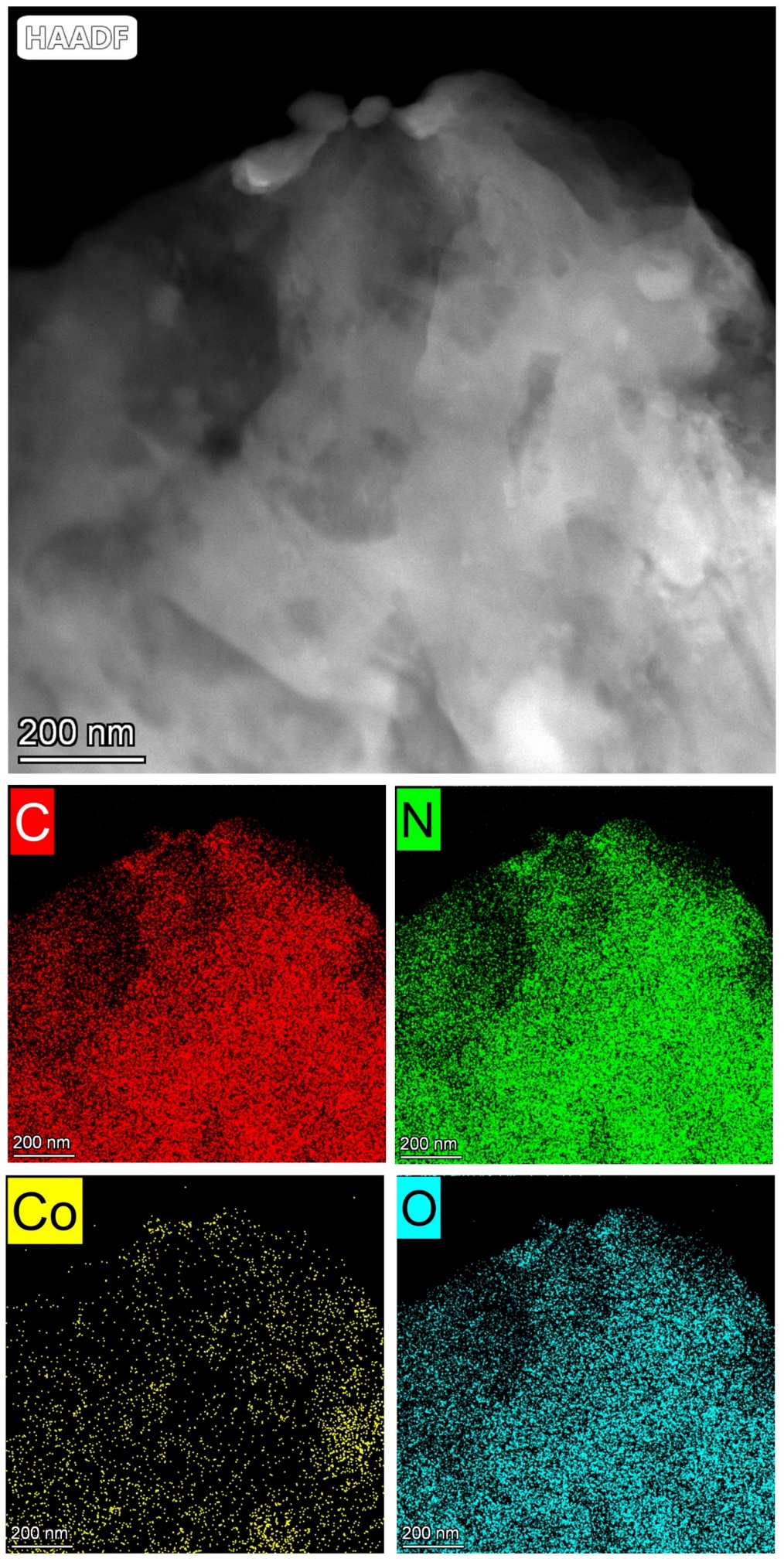

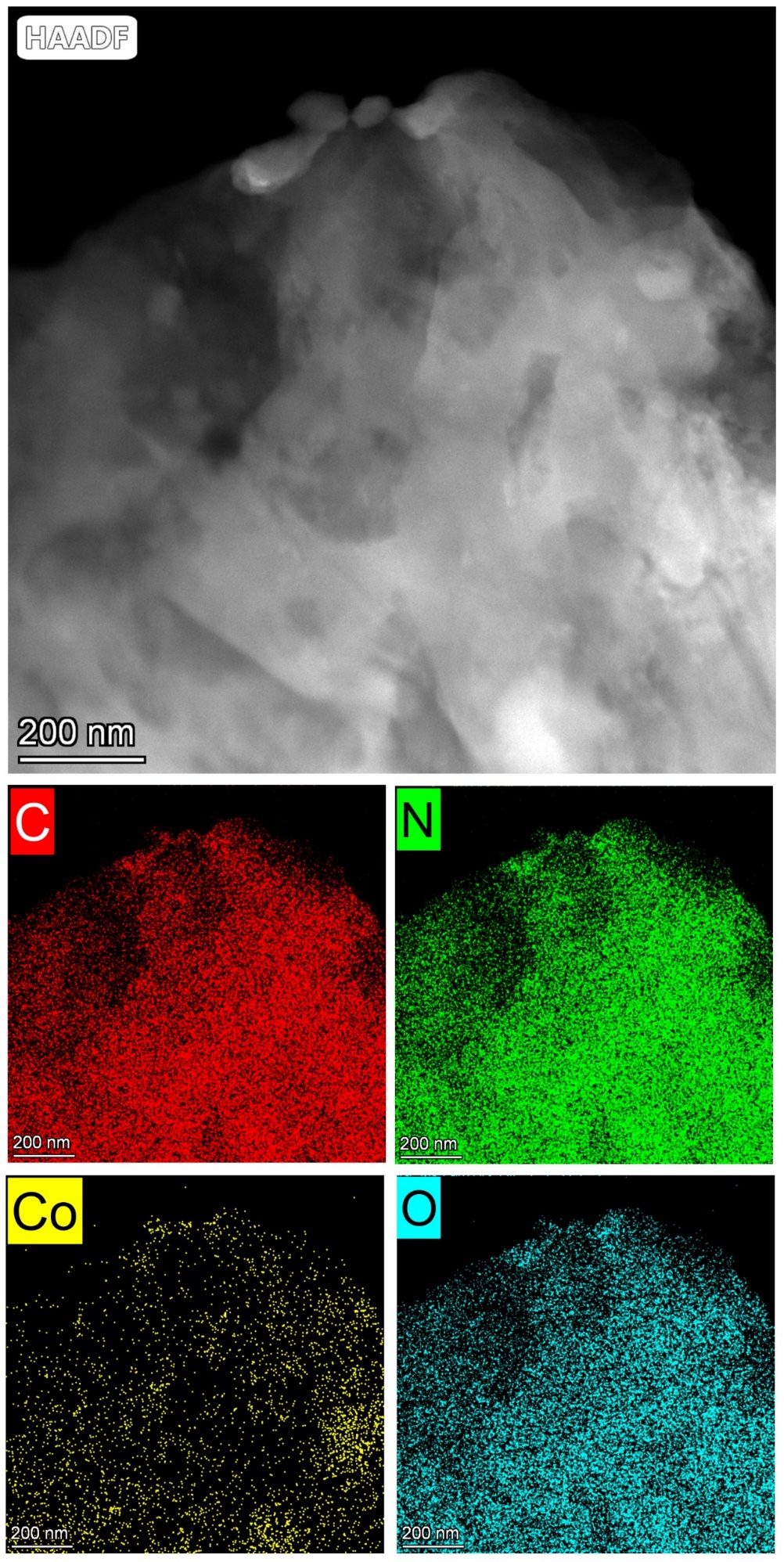
**

**Fig. S9** High-resolution TEM image and elemental mapping images of PI/KNF/GO-20@ZIF-67 aerogel


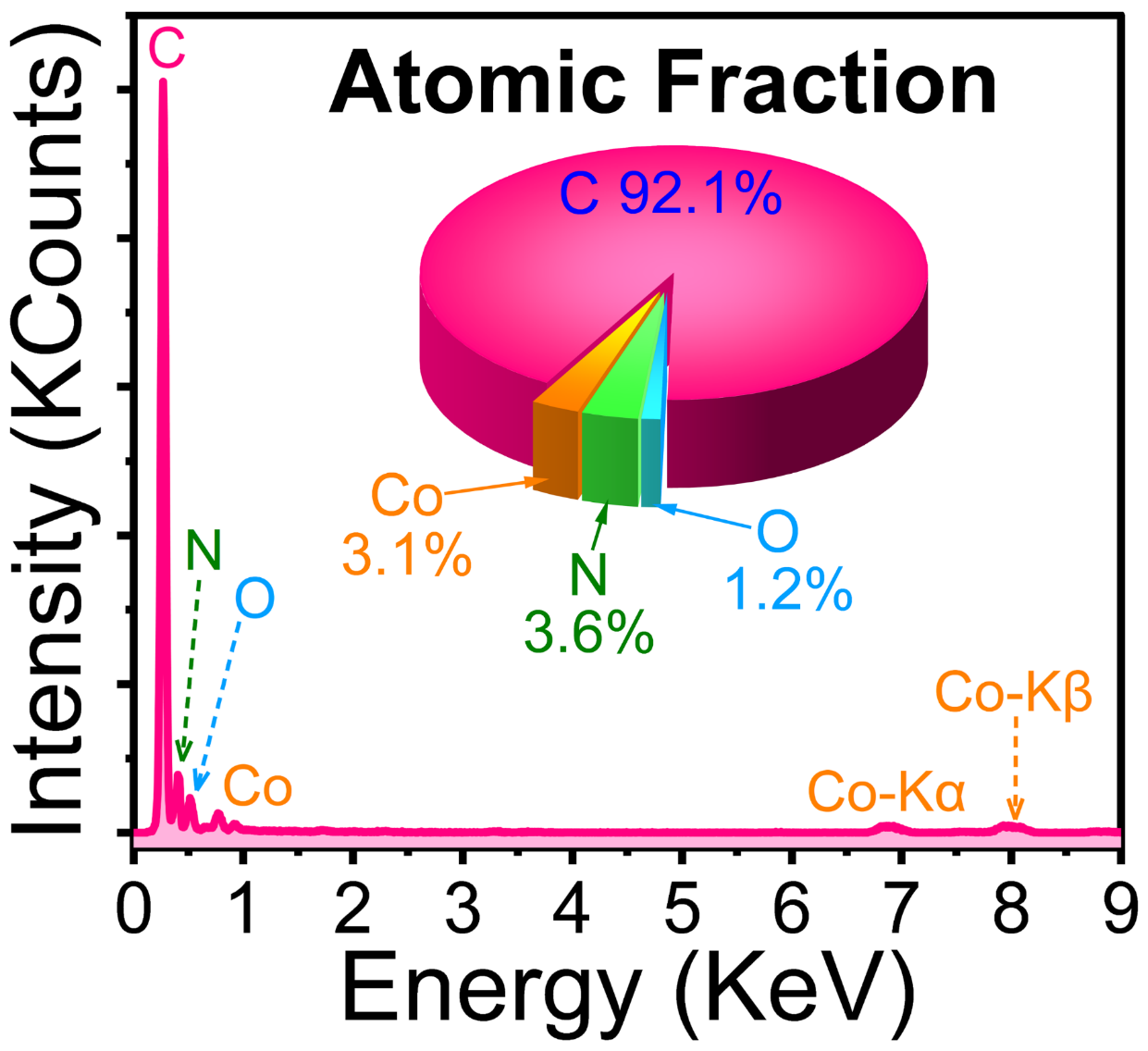


**Fig. S10** EDS spectra and atomic fraction of C/RGO-20@CoNC aerogel obtained in High-resolution TEM mode

**
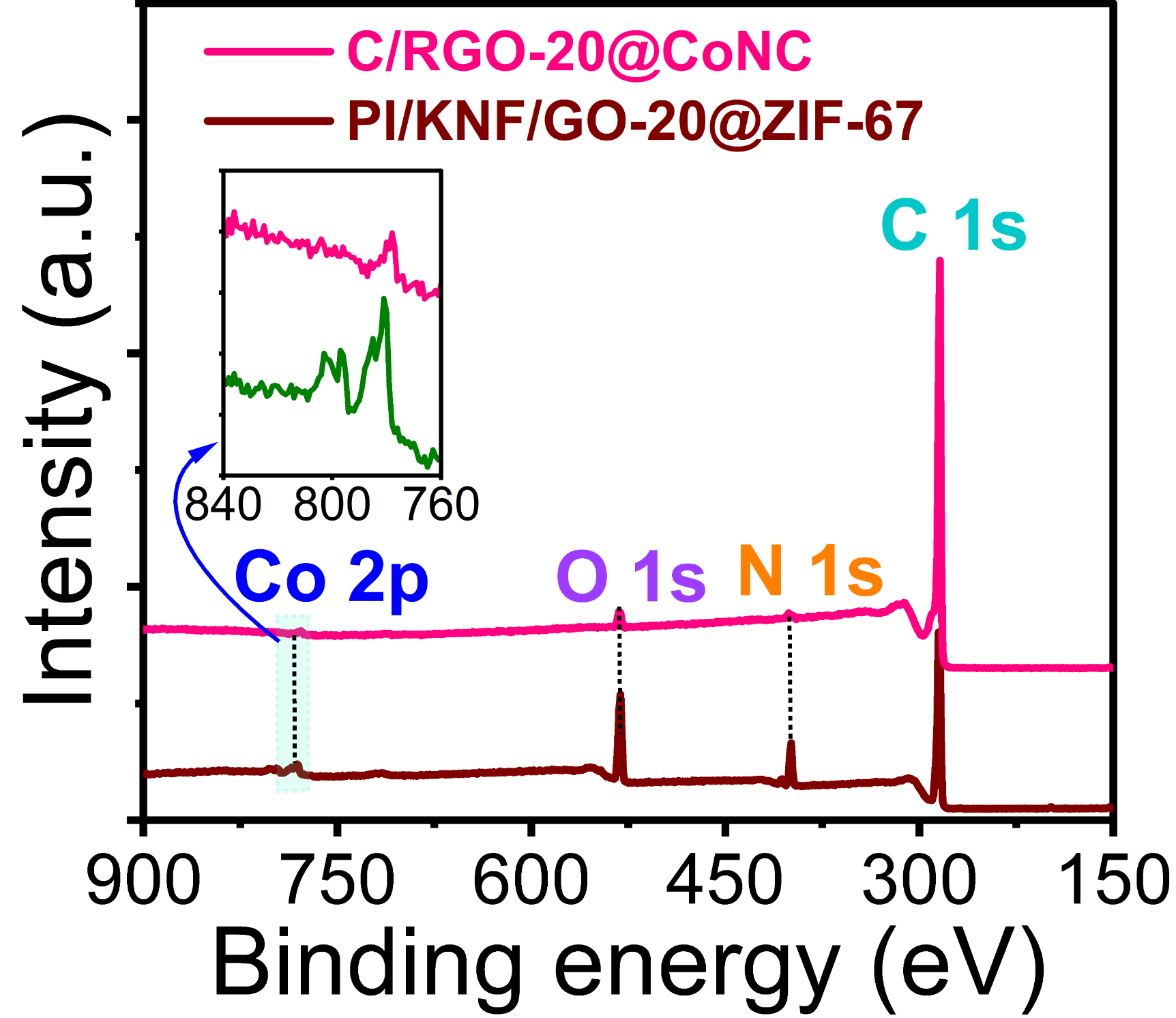
**

**Fig. S11** Survey XPS spectrum of PI/KNF/GO-20@ZIF-67 aerogel and C/RGO-20@CoNC aerogel


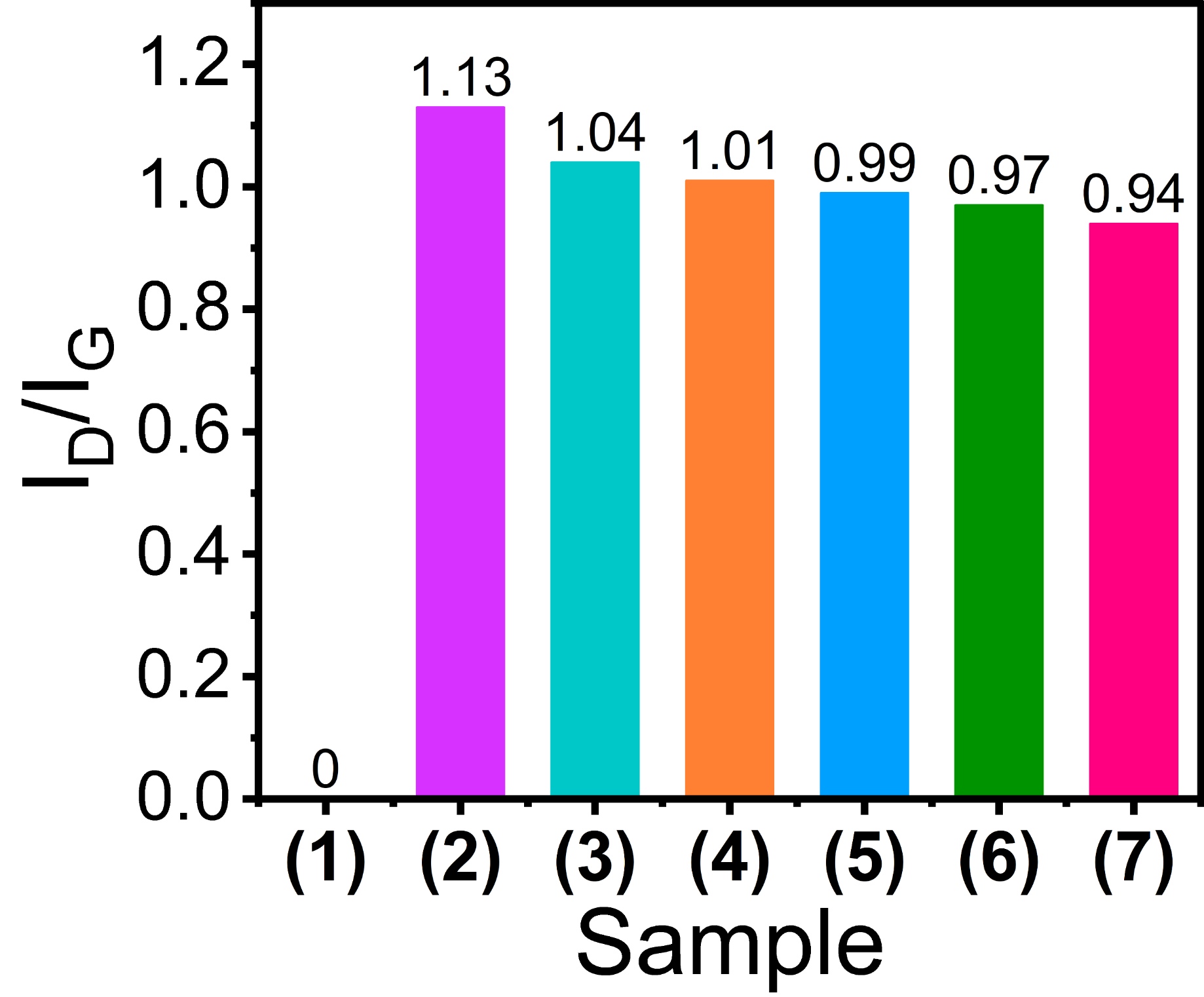


**Fig. S12** I_D_/I_G_ ratio of (1) PI/KNF/GO-20@ZIF-67, (2) C/RGO-20, (3) C@CoNC, (4) C/RGO-5@CoNC, (5) C/RGO-10@CoNC, (6) C/RGO-15@CoNC and (7) C/RGO-20@CoNC aerogels obtained Raman patterns


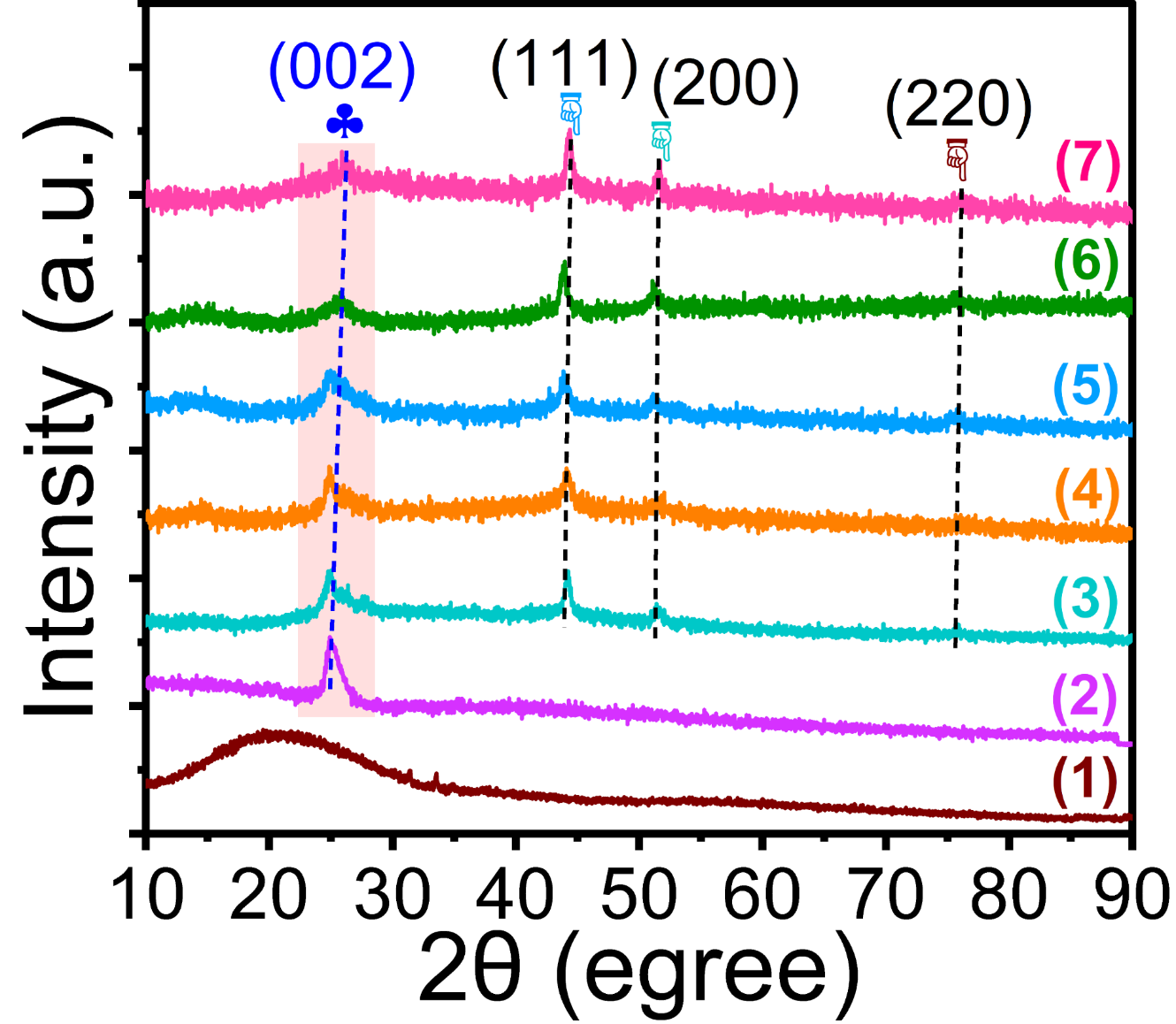


**Fig. S1****3** XRD patterns of (1) PI/KNF/GO-20@ZIF-67, (2) C/RGO-20, (3) C@CoNC, (4) C/RGO-5@CoNC, (5) C/RGO-10@CoNC, (6) C/RGO-15@CoNC and (7) C/RGO-20@CoNC aerogels

**
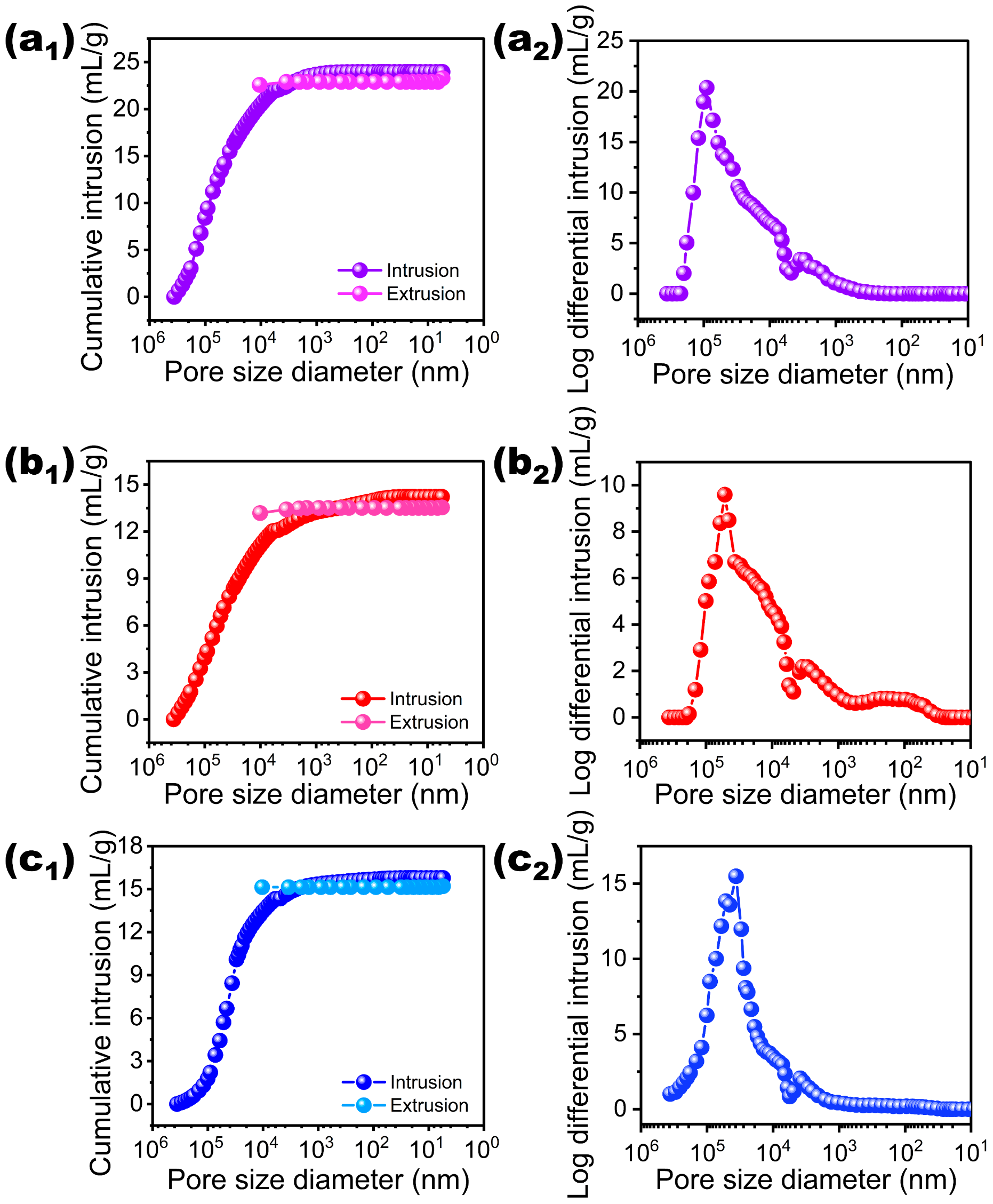
**

**Fig. S14** Mercury intrusion curves of **a_1_** PI/KNF, **b_1_** PI/KNF/GO-20@ZIF-67, **c_1_** C/RGO-20@CoNC aerogel and pore size distribution curves of **a_2_** PI/KNF/GO-20, **b_2_** PI/KNF/GO-20@ZIF-67, **c_2_** C/RGO-20@CoNC aerogel


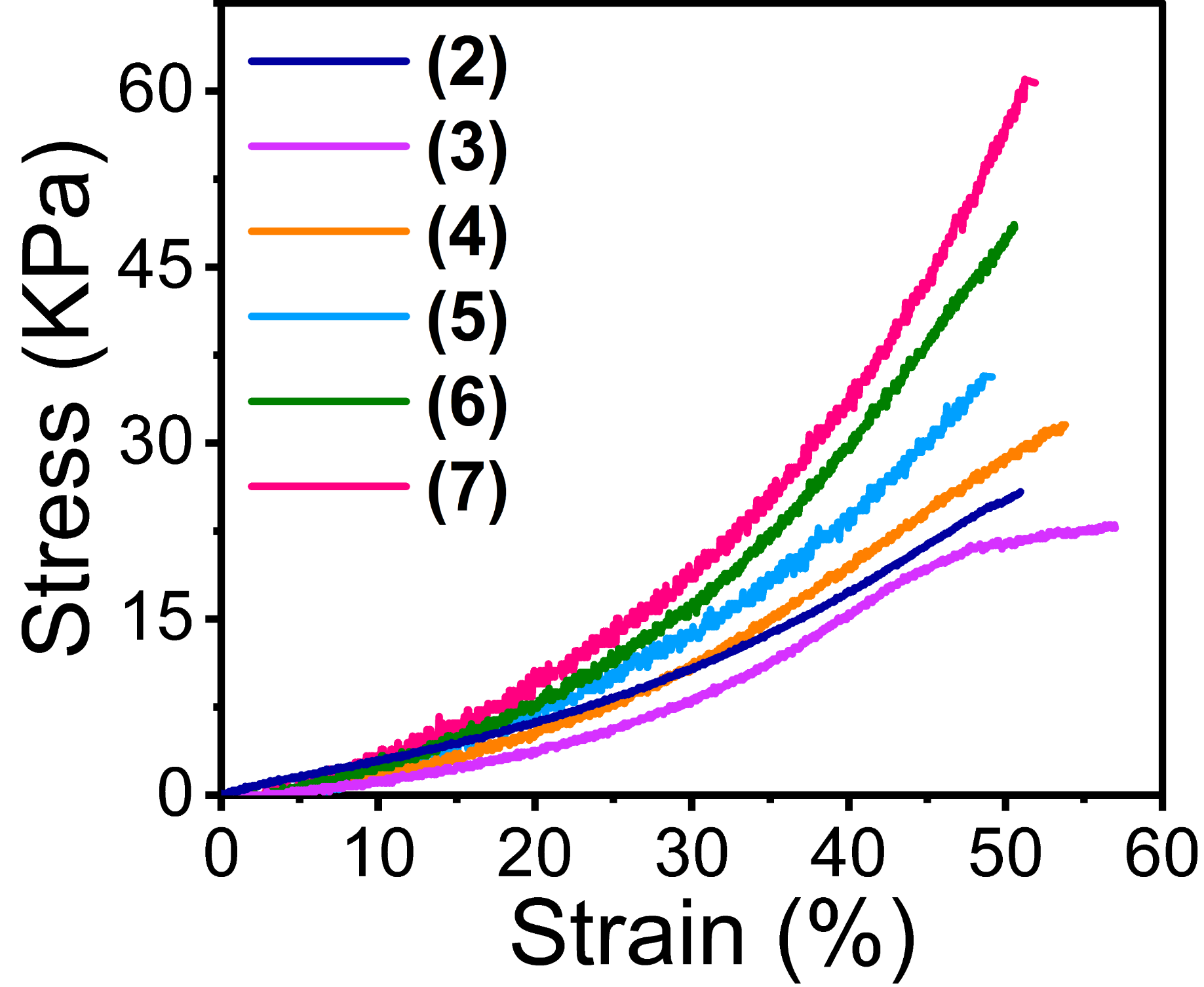


**Fig. S15** Stress-strain curves of (2) C/RGO-20, (3) C@CoNC, (4) C/RGO-5@CoNC, (5) C/RGO-10@CoNC, (6) C/RGO-15@CoNC, and (7) C/RGO-20@CoNC aerogels

**
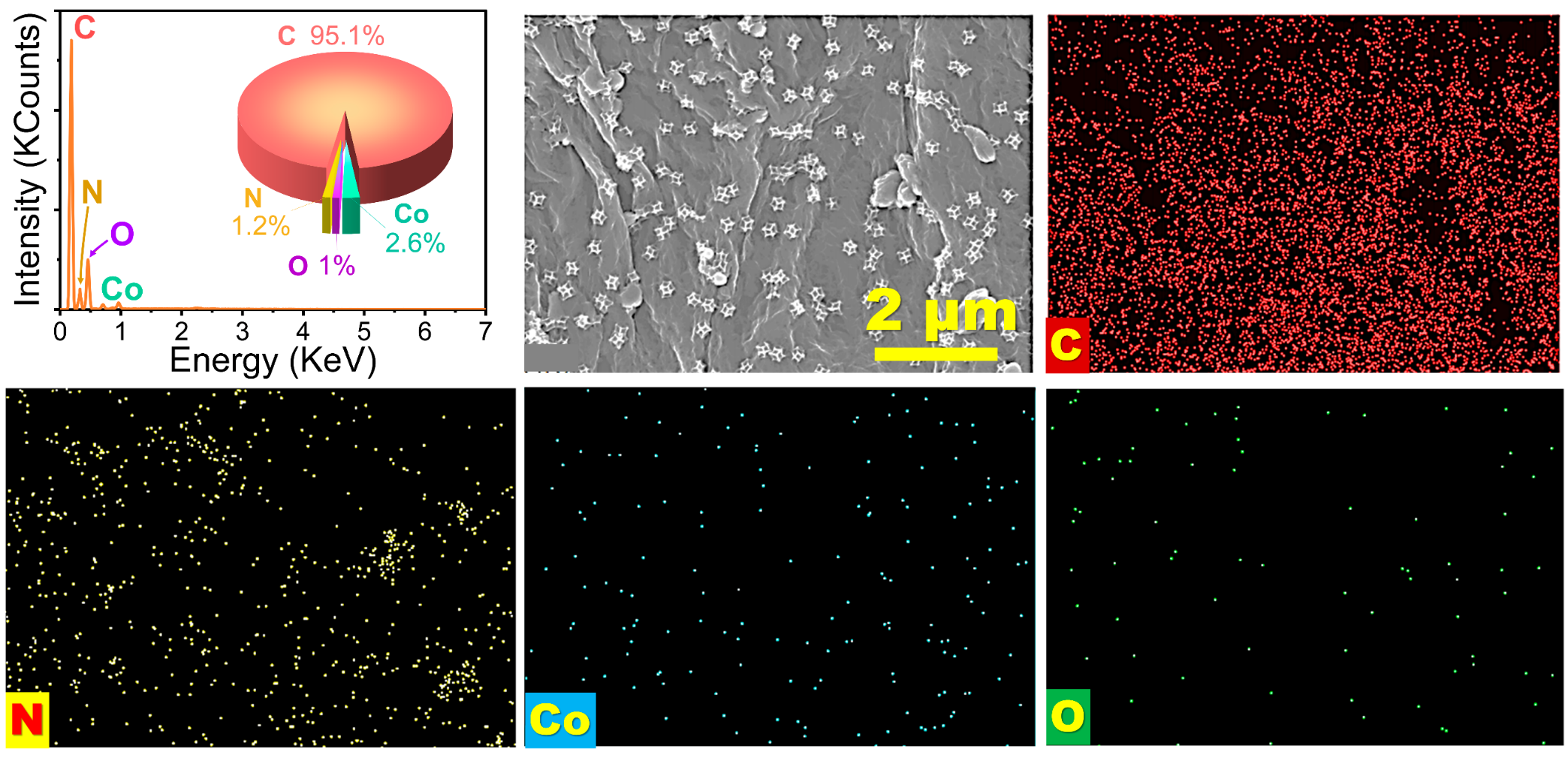
**

**Fig. S16** EDX spectra and element mapping images (C, N, Co and O) of C/RGO-20@CoNC aerogel/PW composite


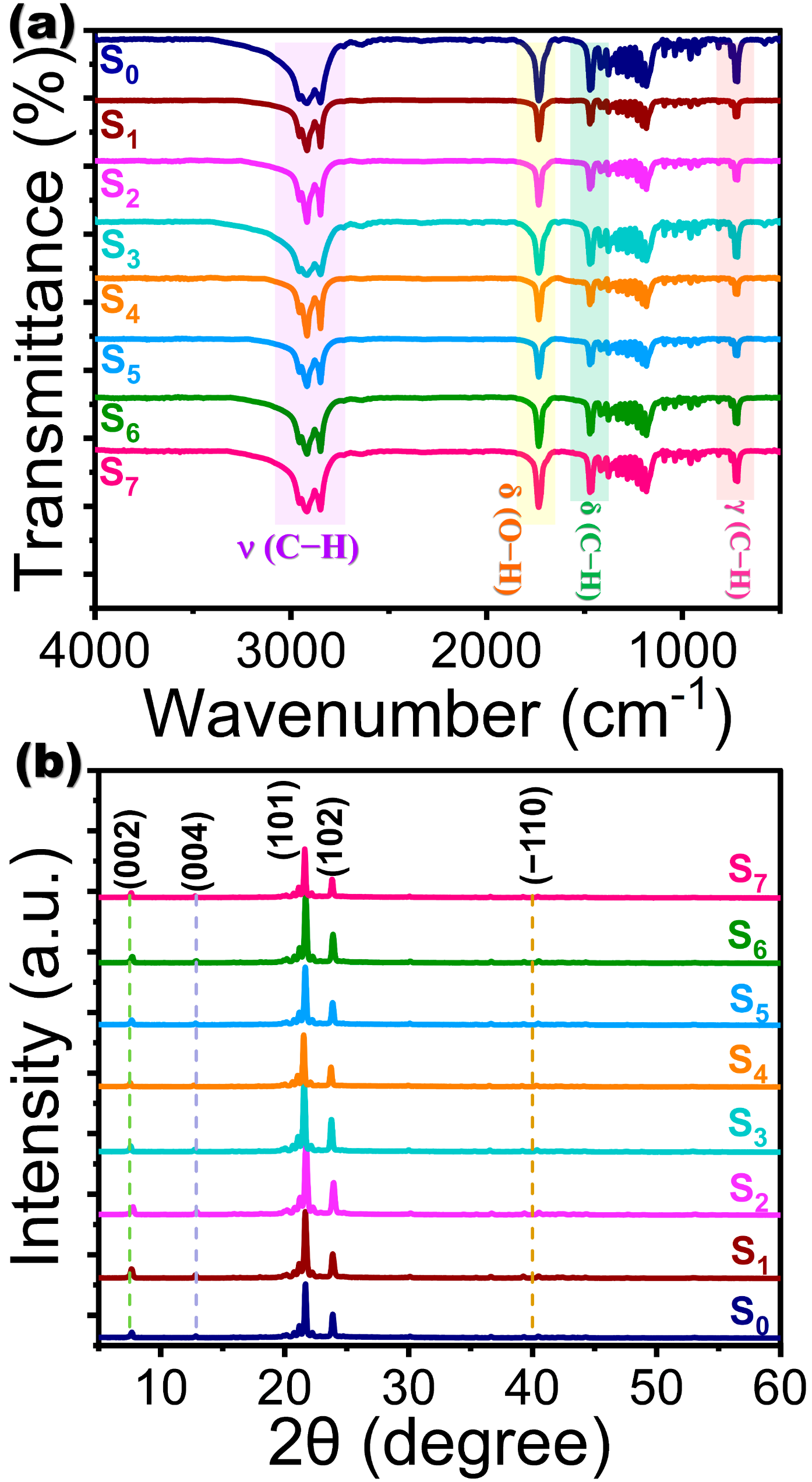


**Fig. S17 a** FTIR spectra and **b** XRD patterns of (S_0_) pure PW and (S_1_) PI/KNF/GO-20@ZIF-67/PW, (S_2_) carbonized PI/KNF aerogel/PW, (S_3_) C@CoNC/PW, (S_4_) C/RGO-5@CoNC/PW, (S_5_) C/RGO-10@CoNC/PW, (S_6_) C/RGO-15@CoNC/PW, and (S_7_) C/RGO-20@CoNC/PW composites


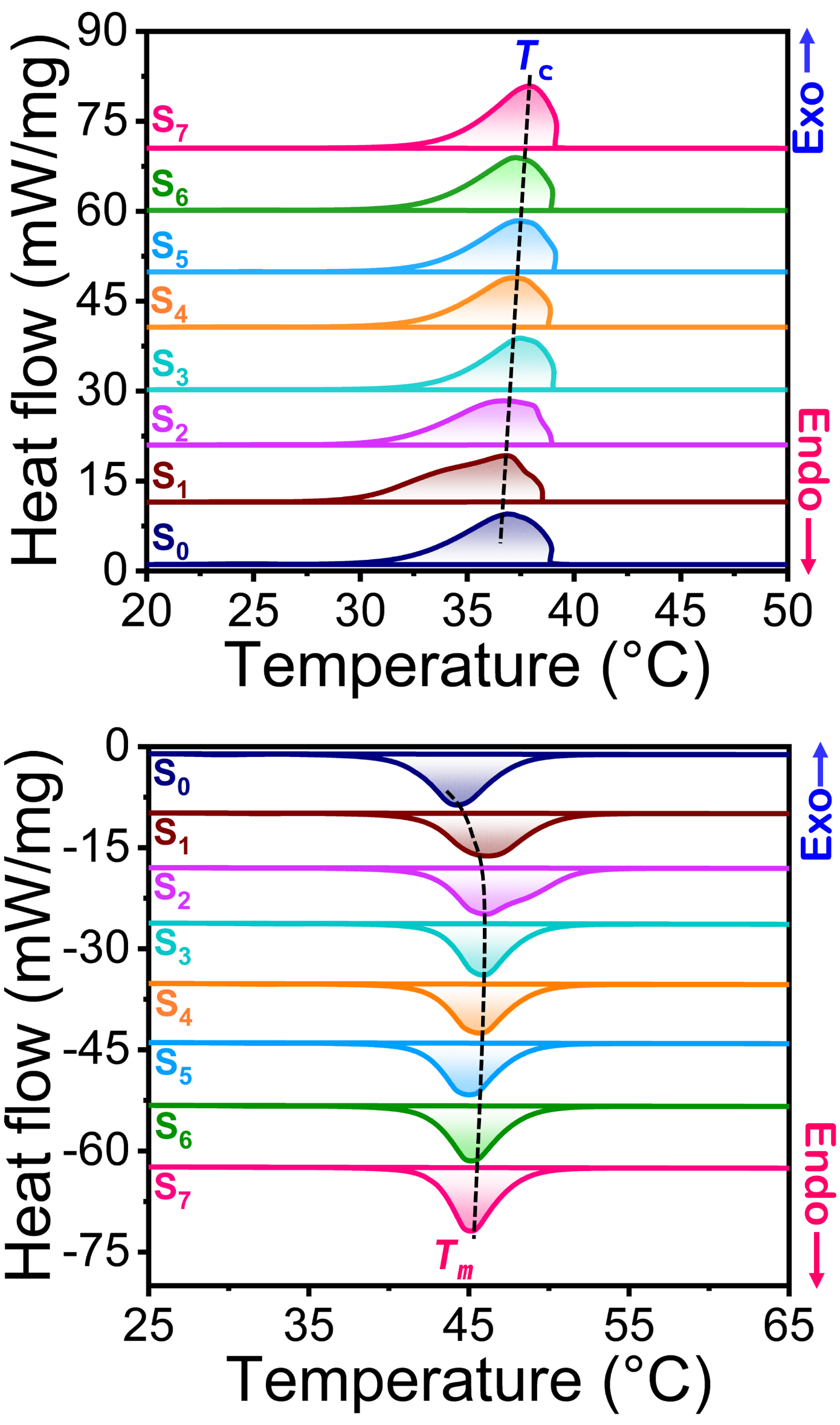


**Fig. S18** DSC heating and cooling thermograms of (S_0_) pure PW and (S_1_) PI/KNF/GO-20@ZIF-67/PW, (S_2_) carbonized PI/KNF aerogel/PW, (S_3_) C@CoNC/PW, (S_4_) C/RGO-5@CoNC/PW, (S_5_) C/RGO-10@CoNC/PW, (S_6_) C/RGO-15@CoNC/PW and (S_7_) C/RGO-20@CoNC/PW composites


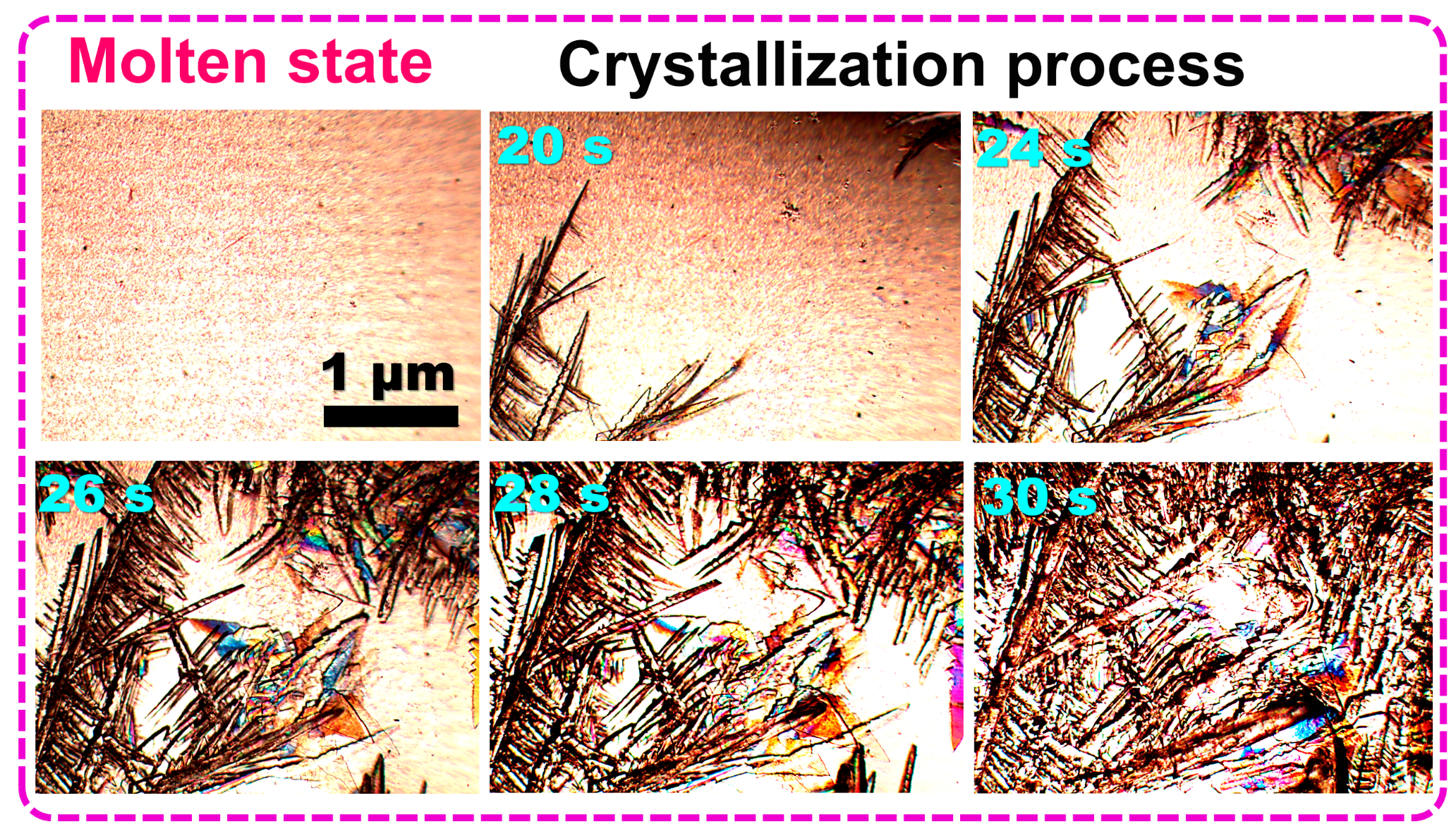


**Fig. S19** Polarized microscope images of pure PW during the heating and cooling processes

**
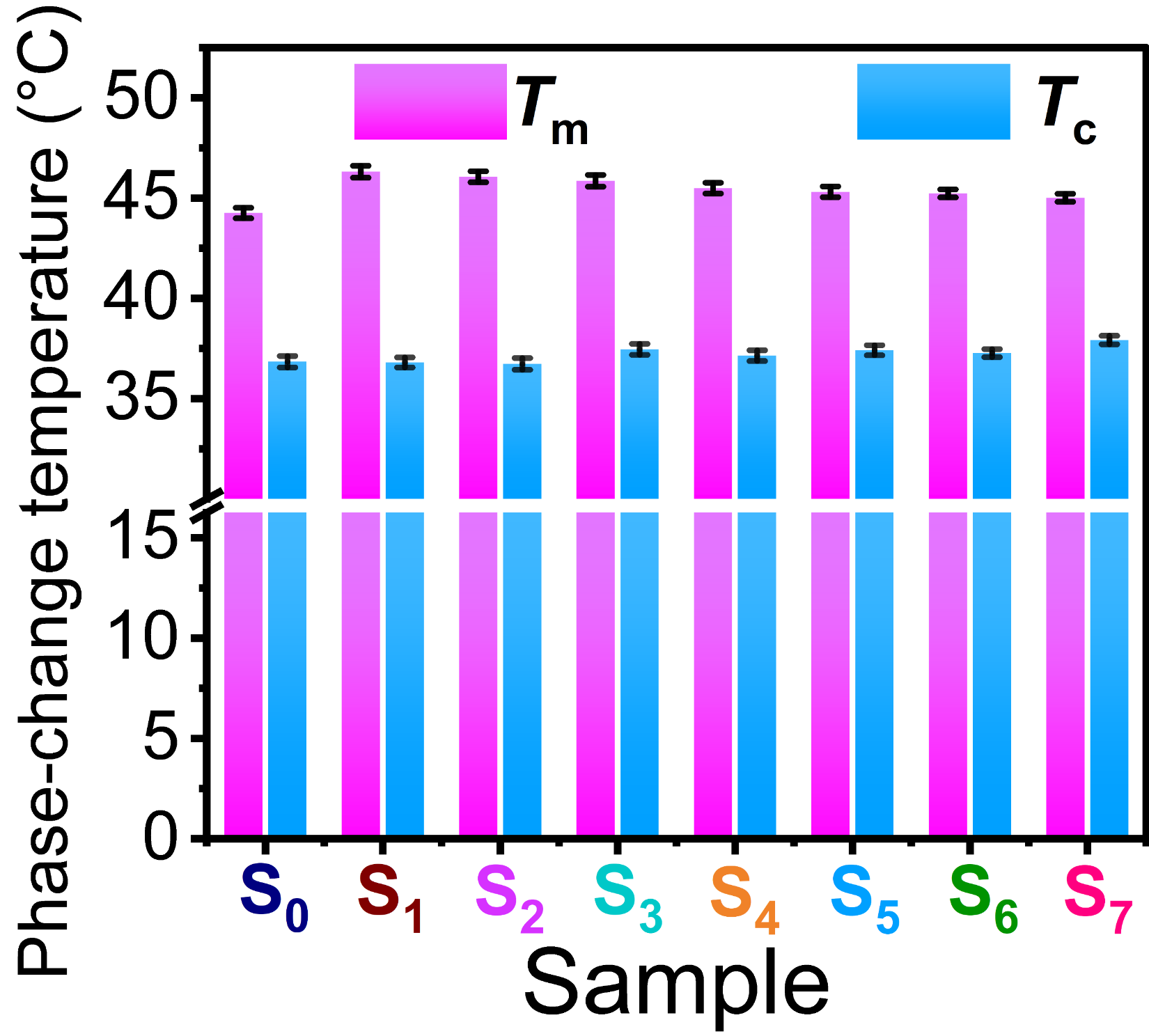
**

**Fig. S****20** Melting and crystallization temperature of (S_0_) pure PW, (S_1_) PI/KNF/GO@ZIF-67/PW, (S_2_) carbonized PI/KNF aerogel/PW, (S_3_) C@CoNC/PW, (S_4_) C/RGO-5@CoNC/PW, (S_5_) C/RGO-10@CoNC/PW, (S_6_) C/RGO-15@CoNC/PW and (S_7_) C/RGO-20@CoNC/PW composites.

**
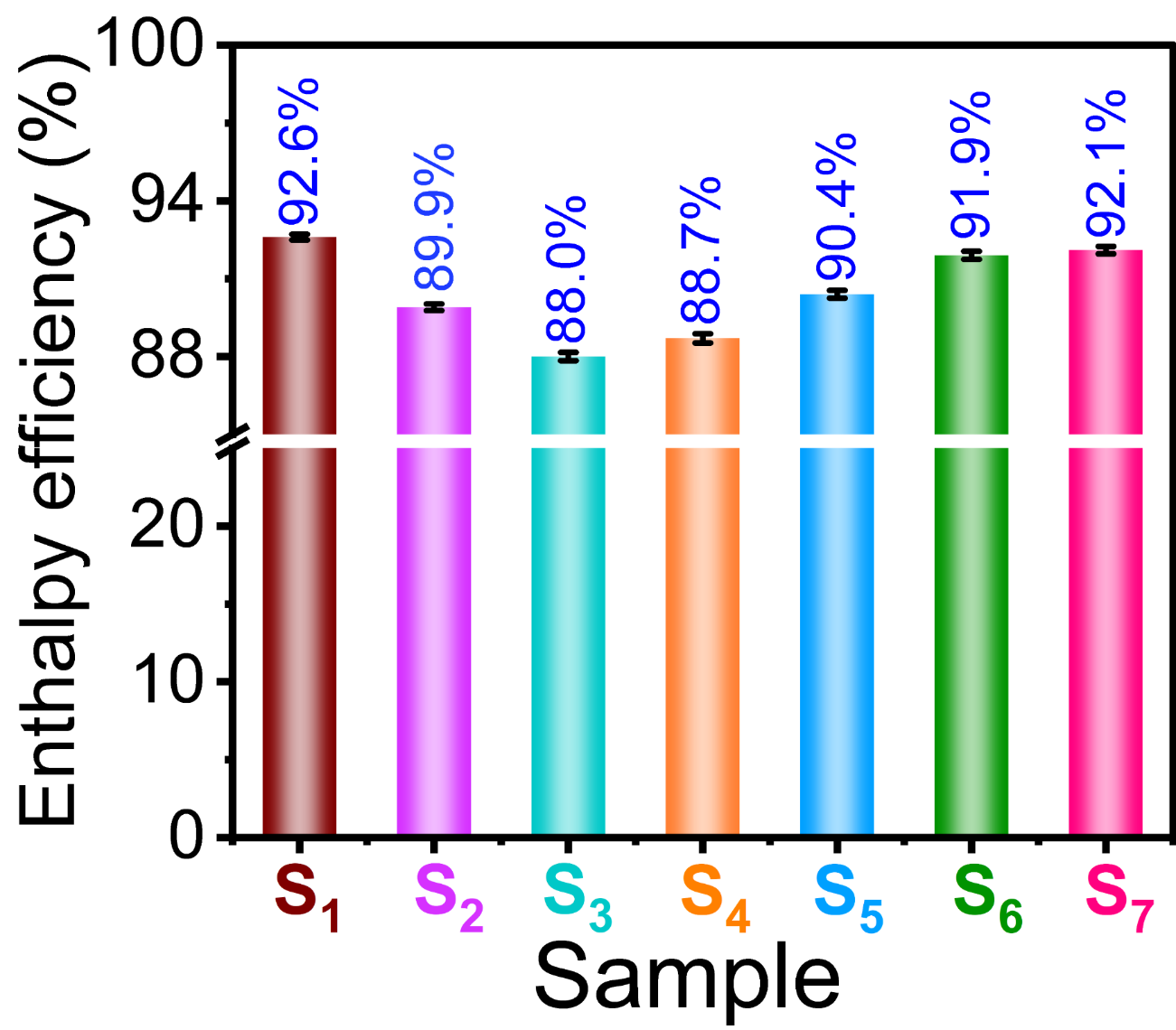
**

**Fig. S21** Enthalpy efficiencies of (S_1_) PI/KNF/GO-20@ZIF-67/PW, (S_2_) carbonized PI/KNF aerogel/PW, (S_3_) C@CoNC/PW, (S_4_) C/RGO-5@CoNC/PW, (S_5_) C/RGO-10@CoNC/PW, (S_6_) C/RGO-15@CoNC/PW and (S_7_) C/RGO-20@CoNC/PW composites


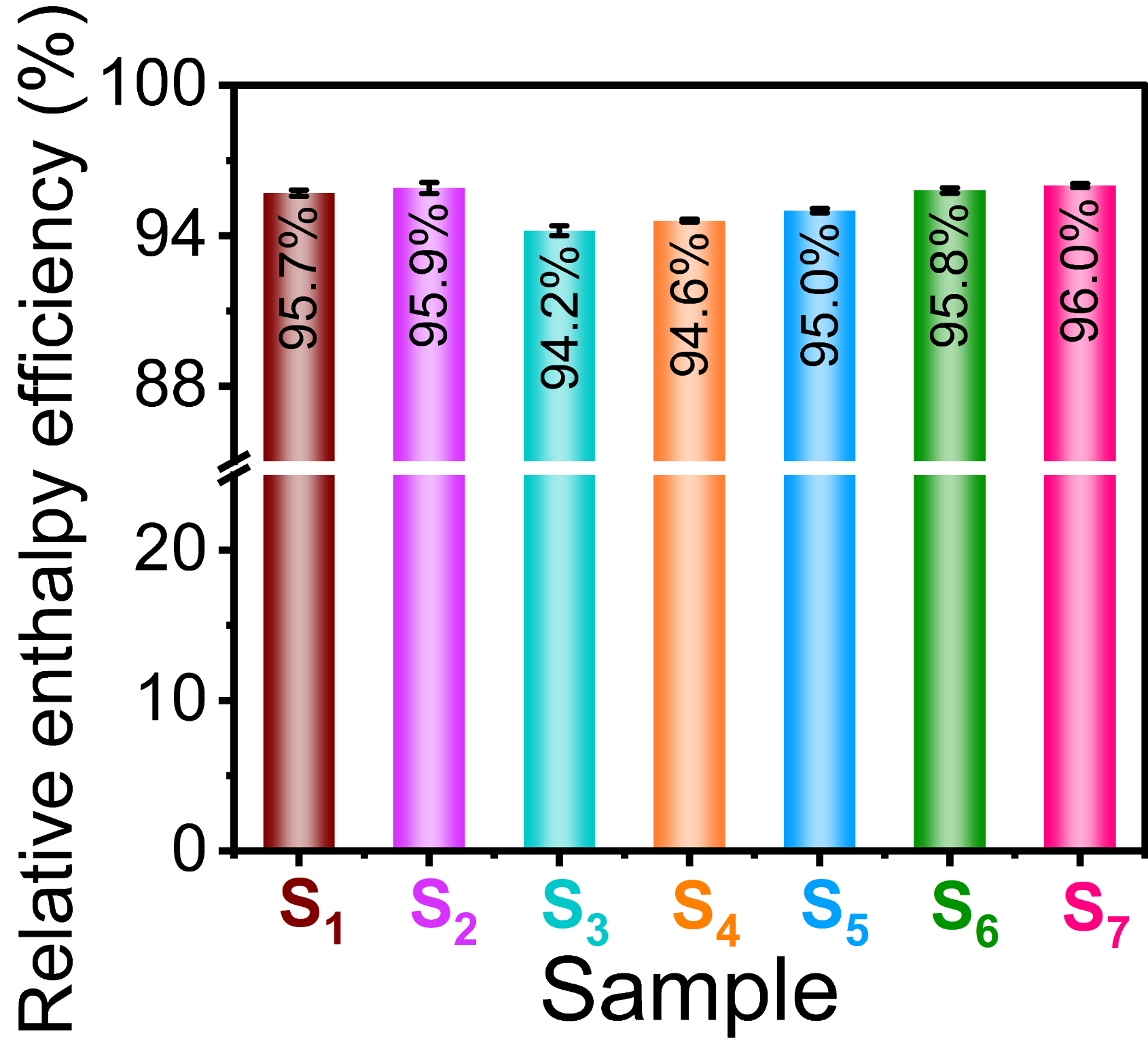


**Fig. S22** Relative enthalpy efficiencies of (S_1_) PI/KNF/GO-20@ZIF-67/PW, (S_2_) carbonized PI/KNF aerogel/PW, (S_3_) C@CoNC/PW, (S_4_) C/RGO-5@CoNC/PW, (S_5_) C/RGO-10@CoNC/PW, (S_6_) C/RGO-15@CoNC/PW and (S_7_) C/RGO-20@CoNC/PW composites


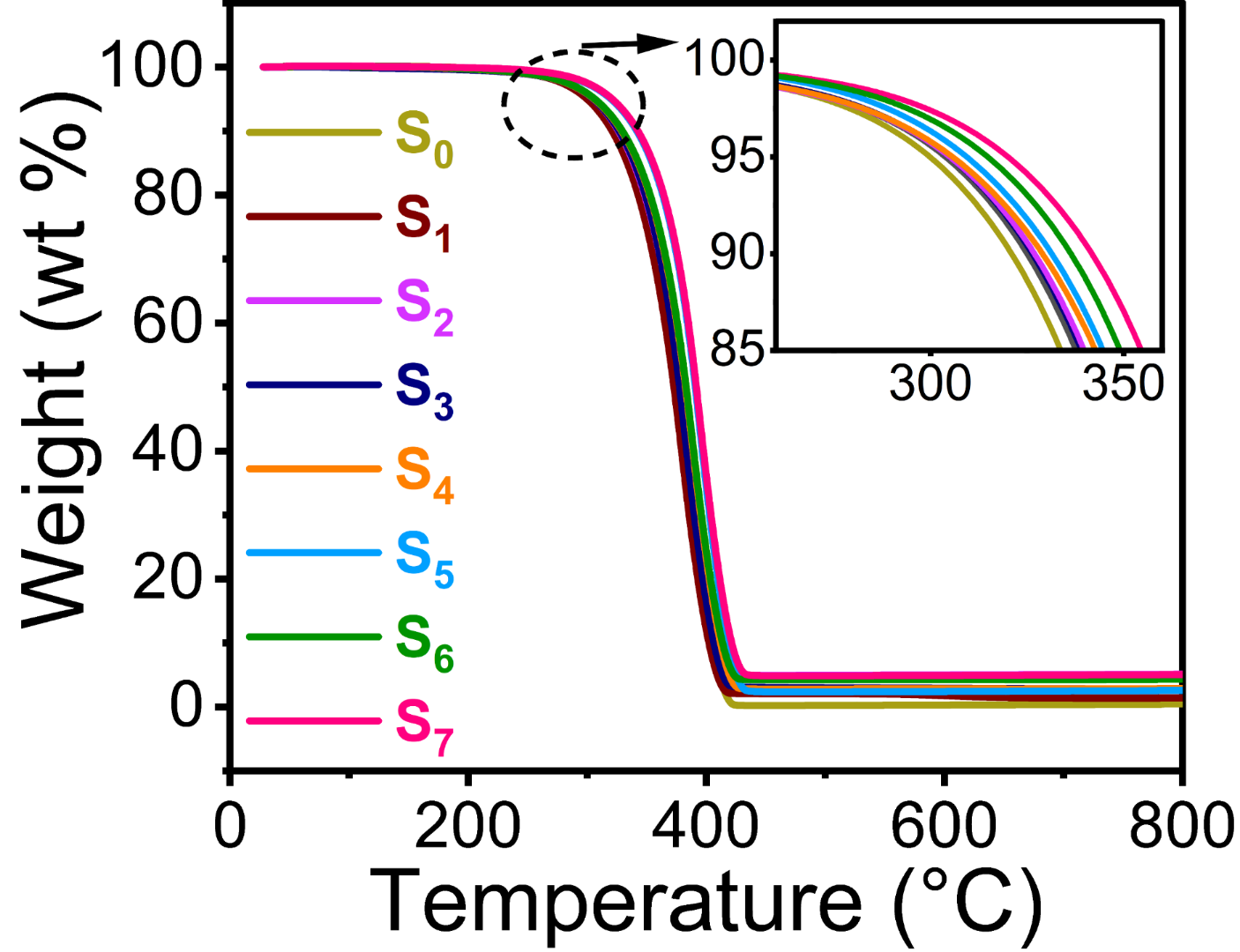


**Fig. S23** TGA thermograms of (S_0_) pure PW and (S_1_) PI/KNF/GO-20@ZIF-67/PW, (S_2_) carbonized PI/KNF aerogel/PW, (S_3_) C@CoNC/PW, (S_4_) C/RGO-5@CoNC/PW, (S_5_) C/RGO-10@CoNC/PW, (S_6_) C/RGO-15@CoNC/PW, and (S_7_) C/RGO-20@CoNC/PW composites


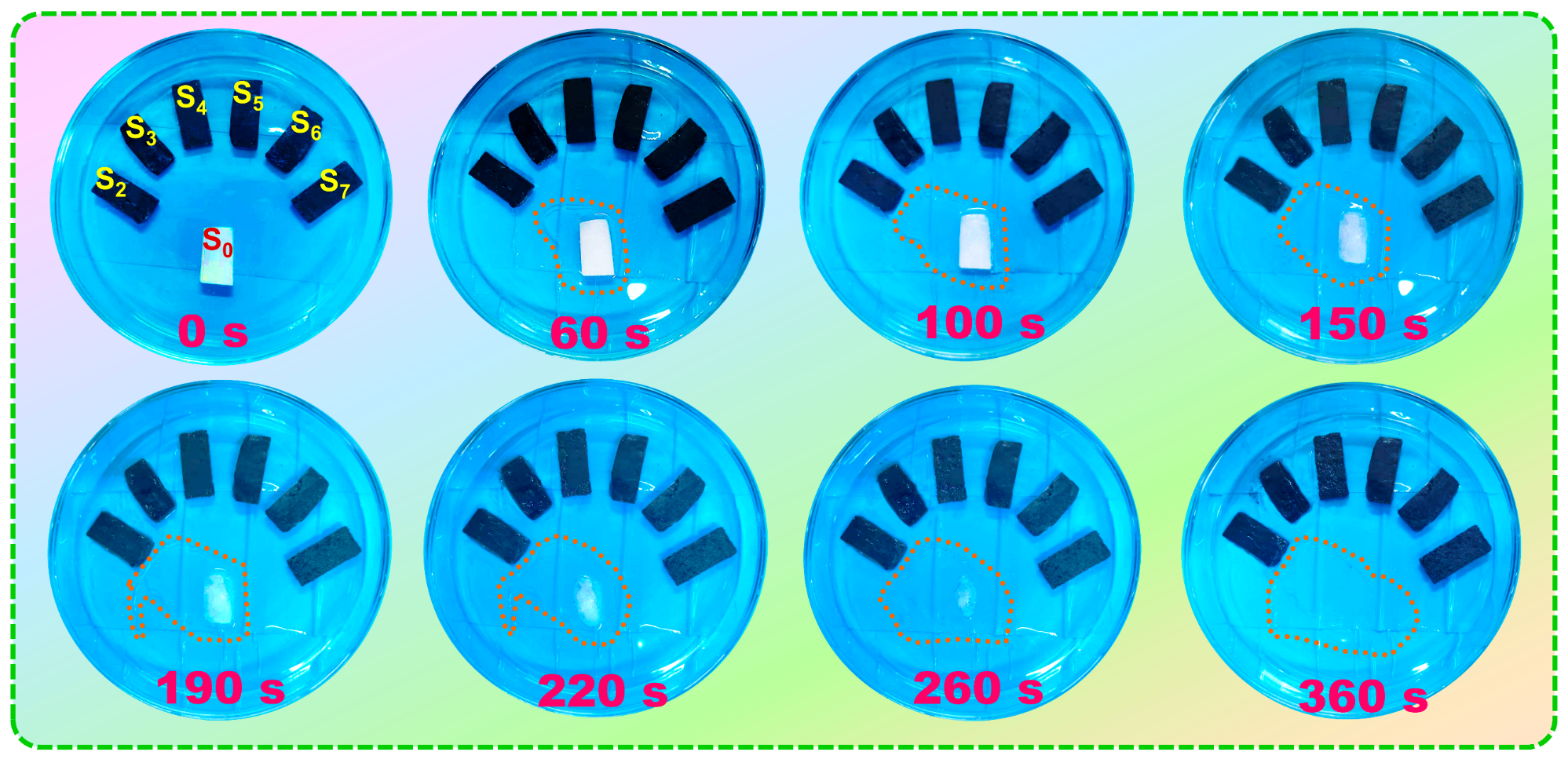


**Fig. S24** Digital photographs of (S_0_) pure PW and (S_2_) carbonized PI/KNF aerogel/PW, (S_3_) C@CoNC/PW, (S_4_) C/RGO-5@CoNC/PW, (S_5_) C/RGO-10@CoNC/PW, (S_6_) C/RGO-15@CoNC/PW, and (S_7_) C/RGO-20@CoNC/PW composites recorded during the isothermal heating process at 120 ℃


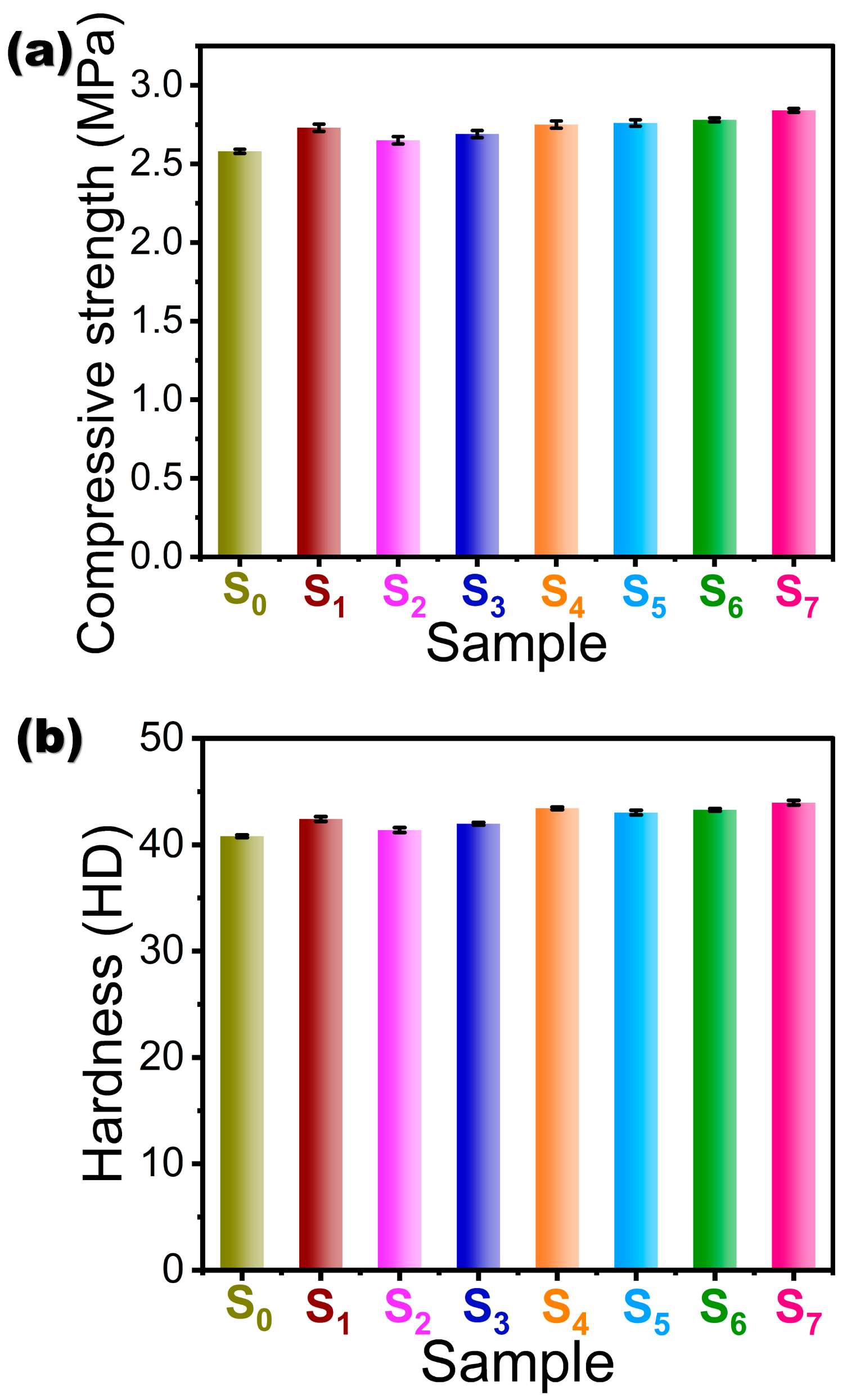


**Fig. S25 a** Compressive strength and **b** hardness of (S_0_) pure PW and (S_1_) PI/KNF/GO-20@ZIF-67/PW, (S_2_) carbonized PI/KNF aerogel/PW, (S_3_) C@CoNC/PW, (S_4_) C/RGO-5@CoNC/PW, (S_5_) C/RGO-10@CoNC/PW, (S_6_) C/RGO-15@CoNC/PW, and (S_7_) C/RGO-20@CoNC/PW composites


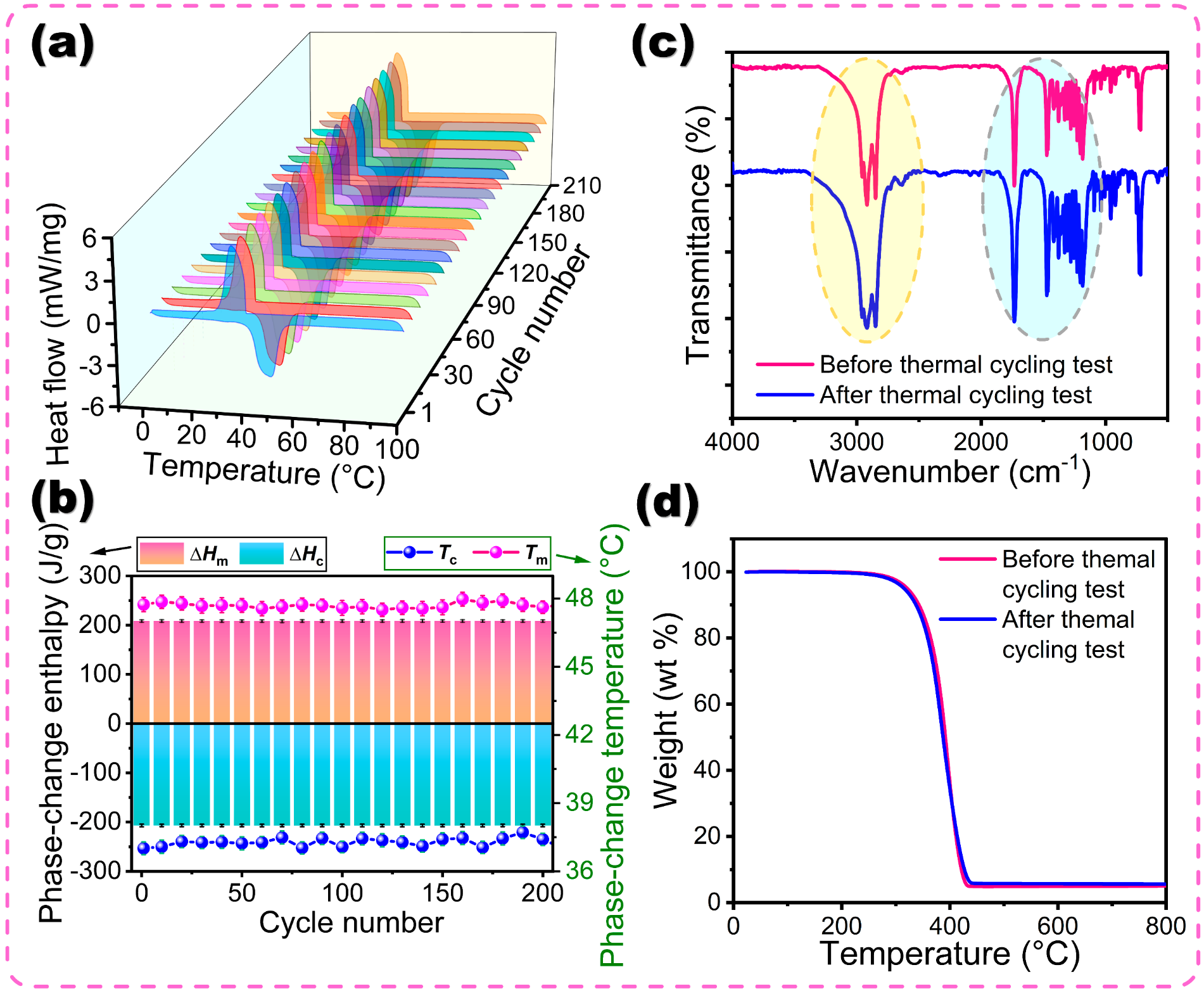


**Fig. S26 a** Multicycle DSC thermograms and **b** phase-change temperatures and enthalpies obtained from the thermal heating/cooling cycling experiment for C/RGO-20@CoNC/PW composite. **c** FTIR spectra and **d** TGA thermograms of C/RGO-20@CoNC/PW composite before and after thermal cycles

**
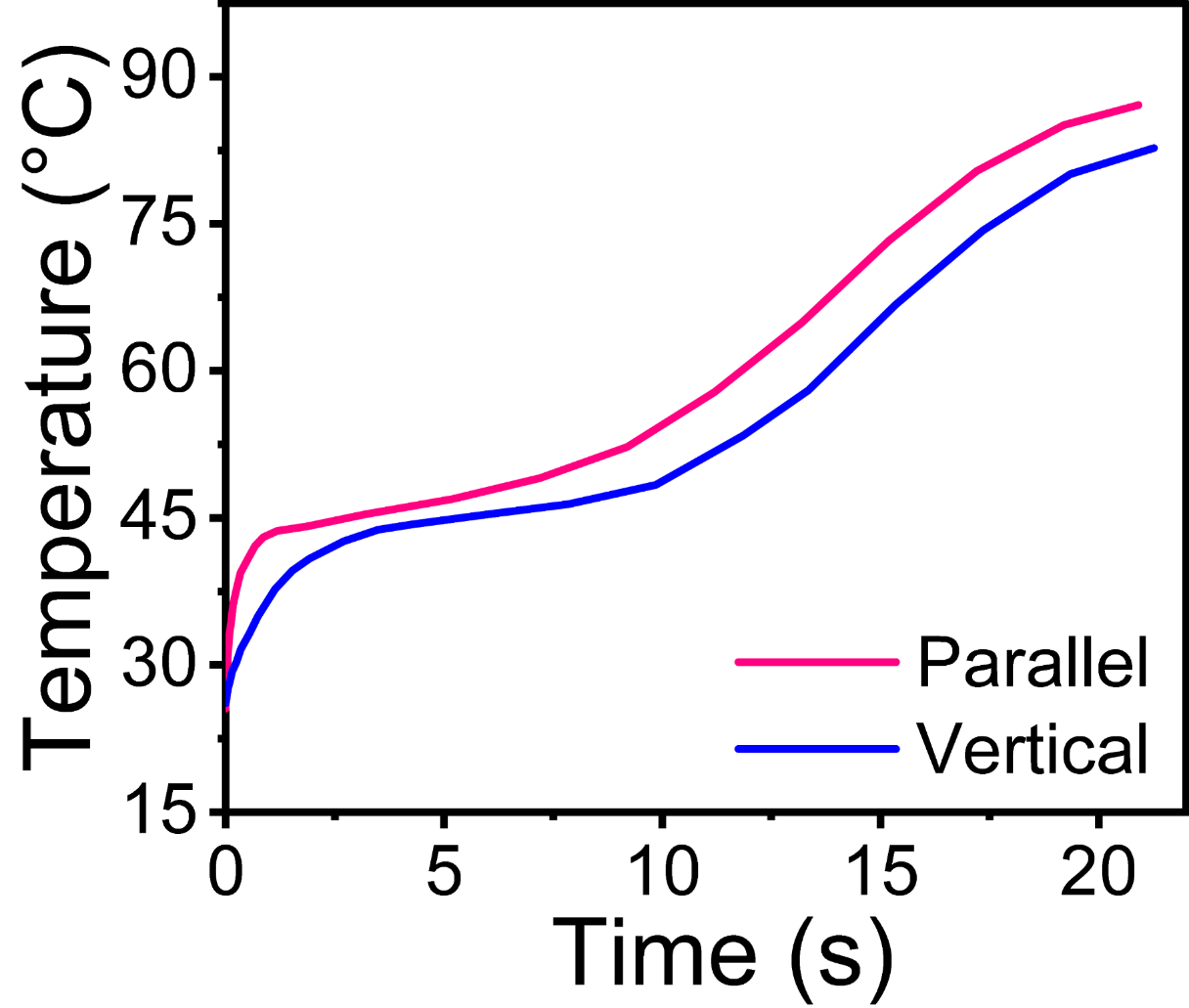
**

**Fig. S27** Temperature-time curves of the center part of C/RGO-20@CoNC/PW composite parallel and vertical to the lamellar pores based on finite element analysis

**
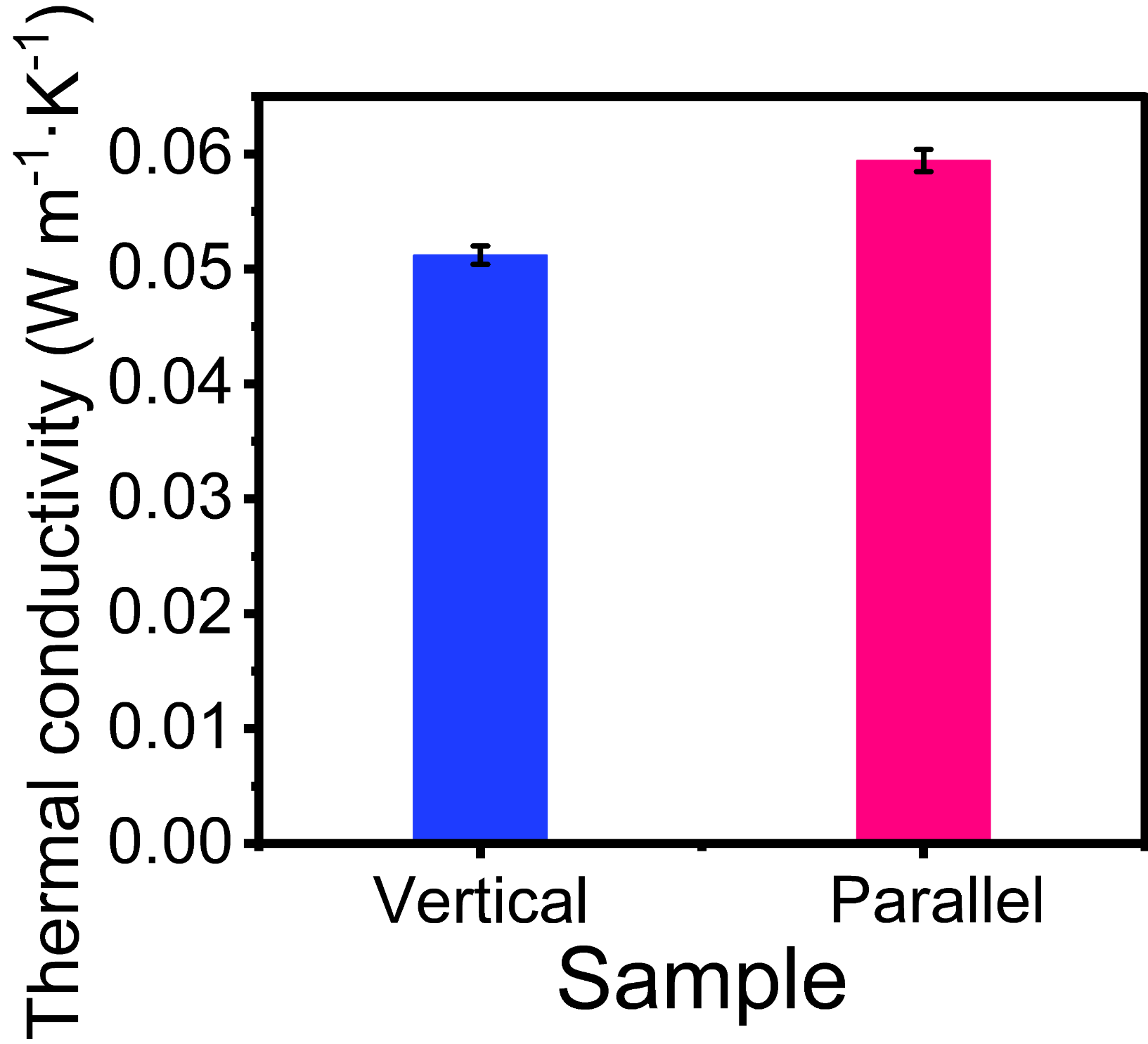
**

**Fig. S28** Thermal conductivities of C/RGO-20@CoNC aerogel vertical and parallel to the lamellar pores


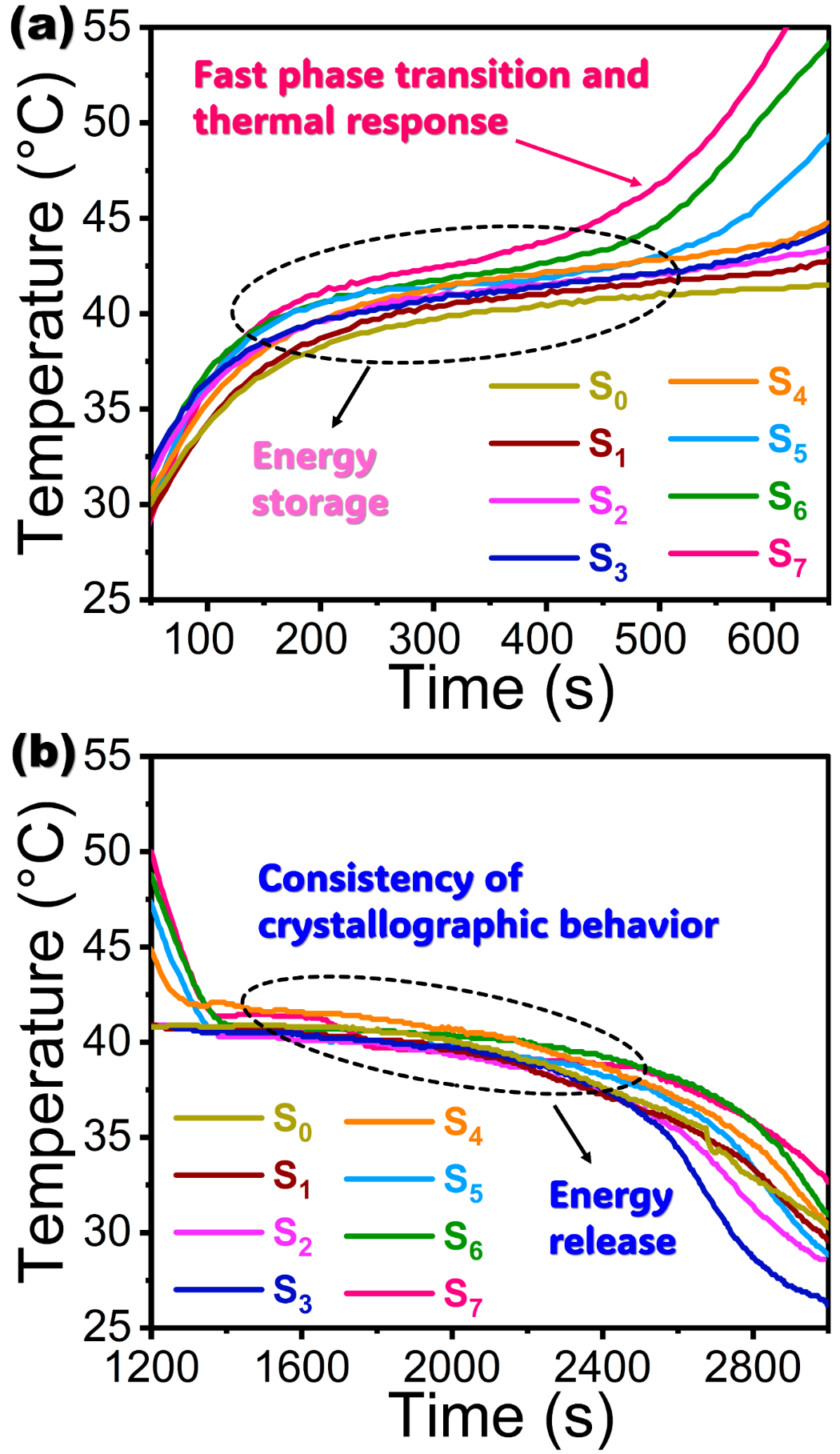

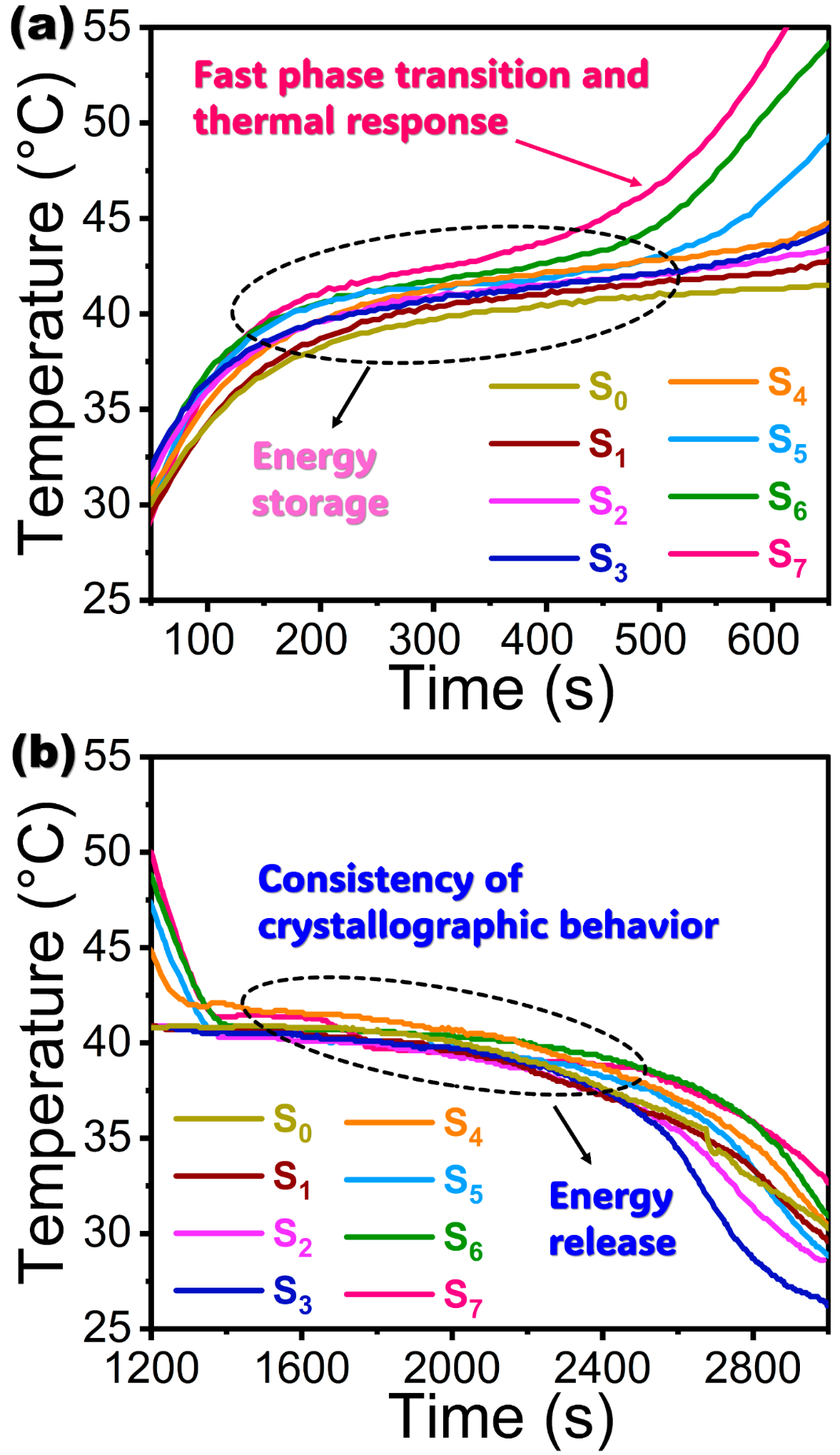


**Fig. S29** Enlarged view of the temperature-time evolutions of (S_0_) pure PW and (S_1_) PI/KNF/GO-20@ZIF-67/PW, (S_2_) carbonized PI/KNF aerogel/PW, (S_3_) C@CoNC/PW, (S_4_) C/RGO-5@CoNC/PW, (S_5_) C/RGO-10@CoNC/PW, (S_6_) C/RGO-15 @CoNC/PW, and (S_7_) C/RGO-20@CoNC/PW composites a under simulated solar illumination and b no irradiation


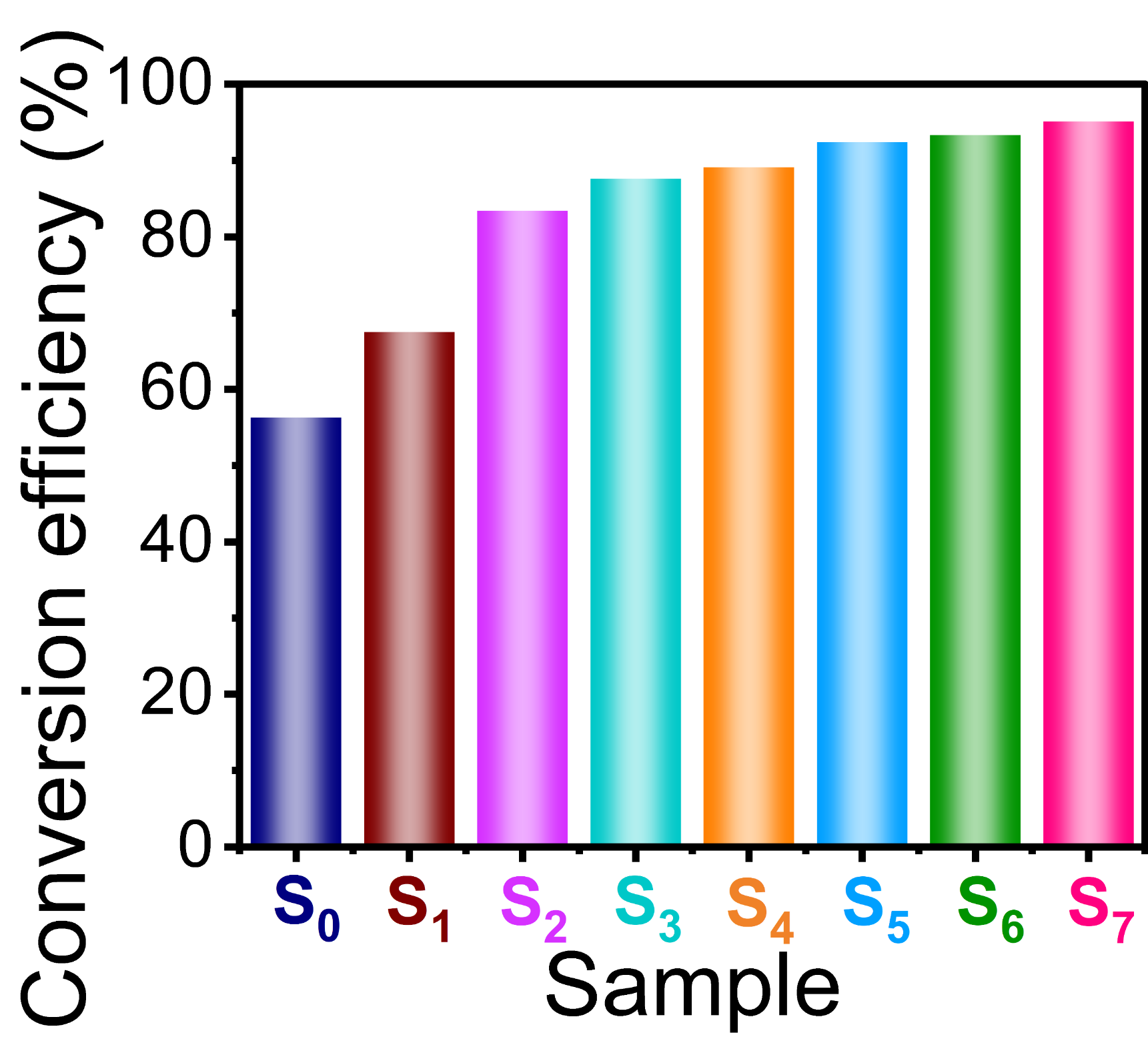


**Fig. S30** Solar-thermal conversion efficiency of (S_0_) pure PW, (S_1_) PI/KNF/GO-20@ZIF-67/PW, (S_2_) carbonized PI/KNF aerogel/PW, (S_3_) C@CoNC/PW, (S_4_) C/RGO-5@CoNC/PW, (S_5_) C/RGO-10@CoNC/PW, (S_6_) C/RGO-15@CoNC/PW and (S_7_) C/RGO-20@CoNC/PW composites


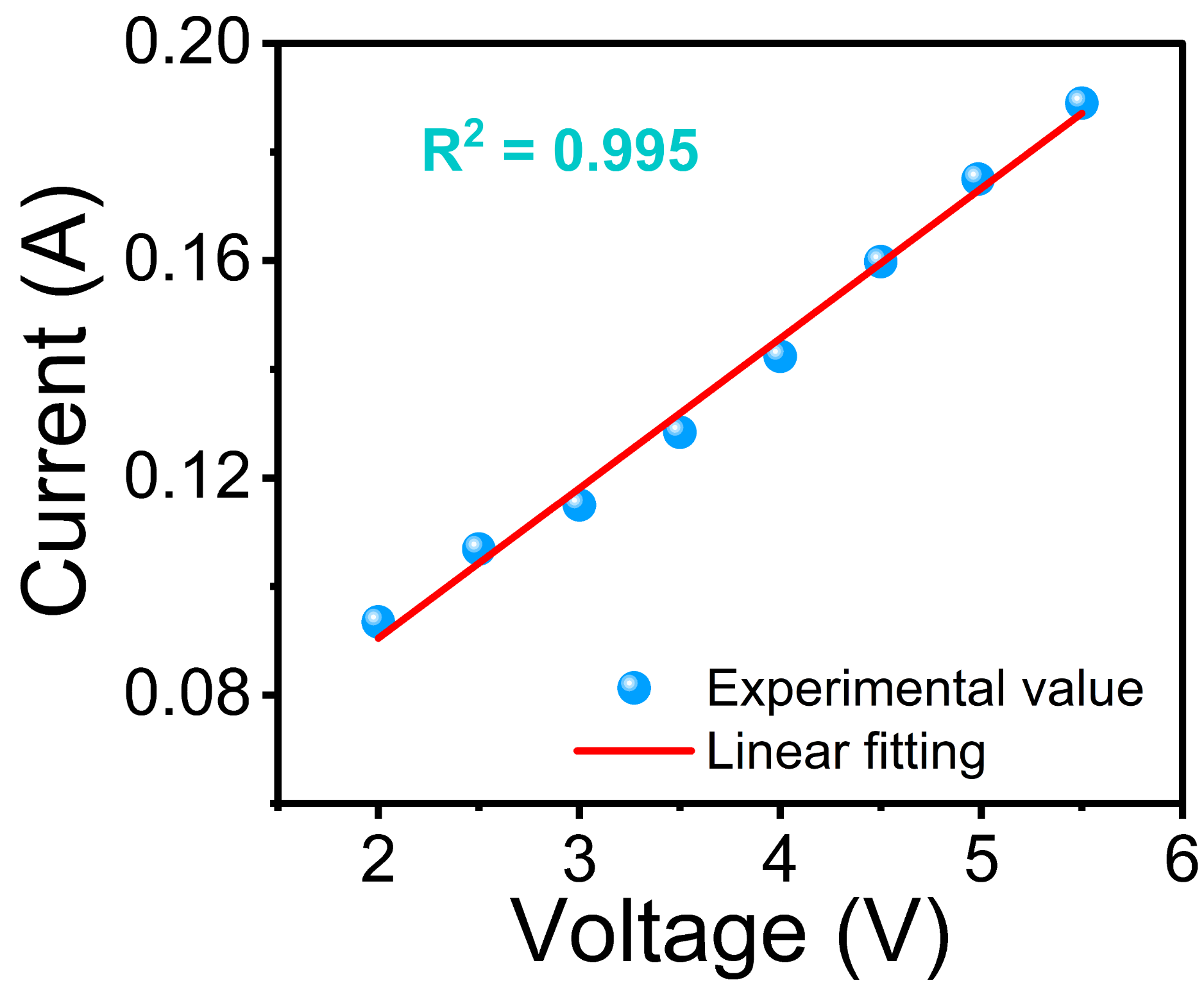


**Fig. S31** V–I linear curve of C/RGO-20@CoNC/PW composite


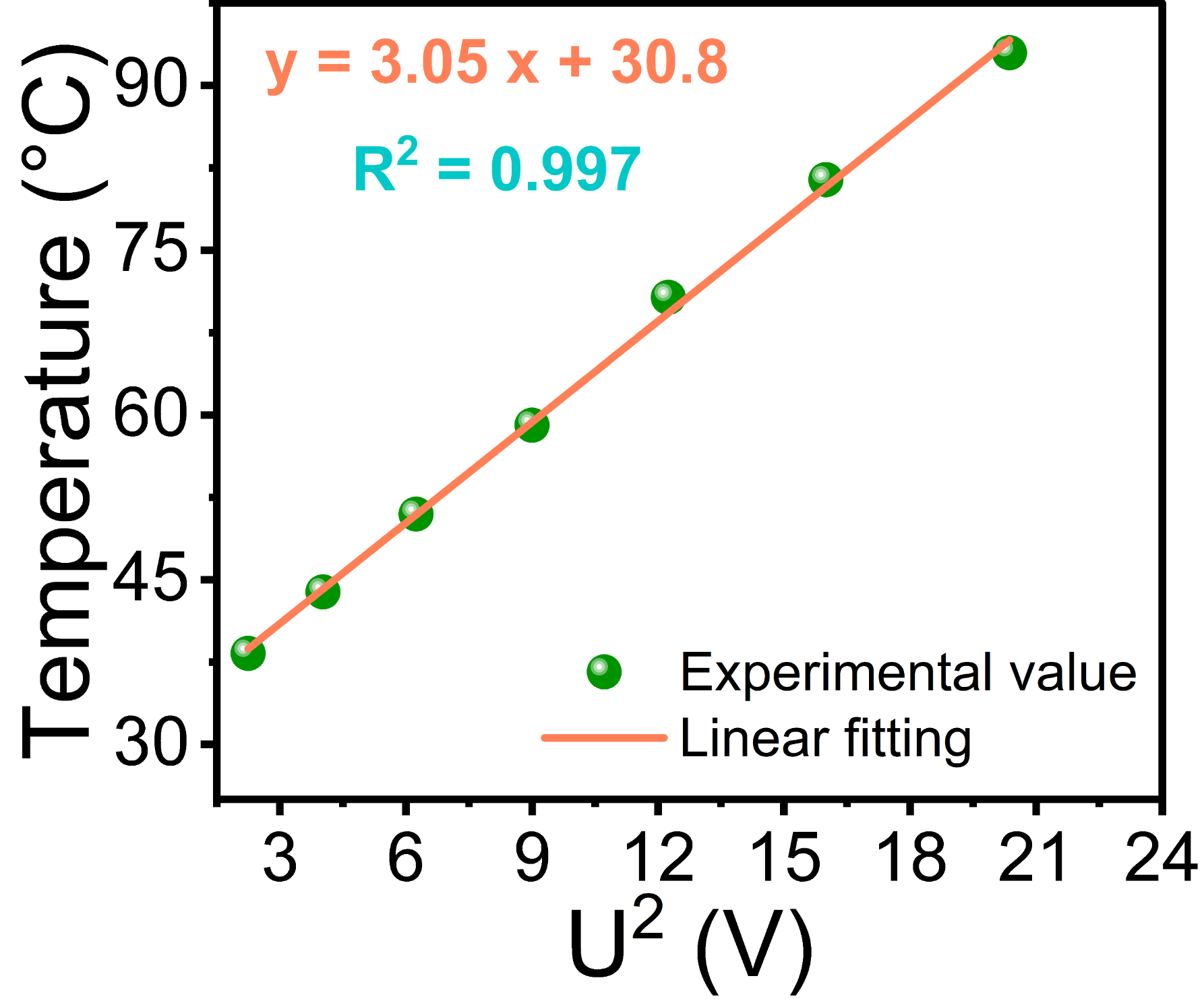


**Fig. S32** Linear fitting and experimental data of stabilized temperature versus U2


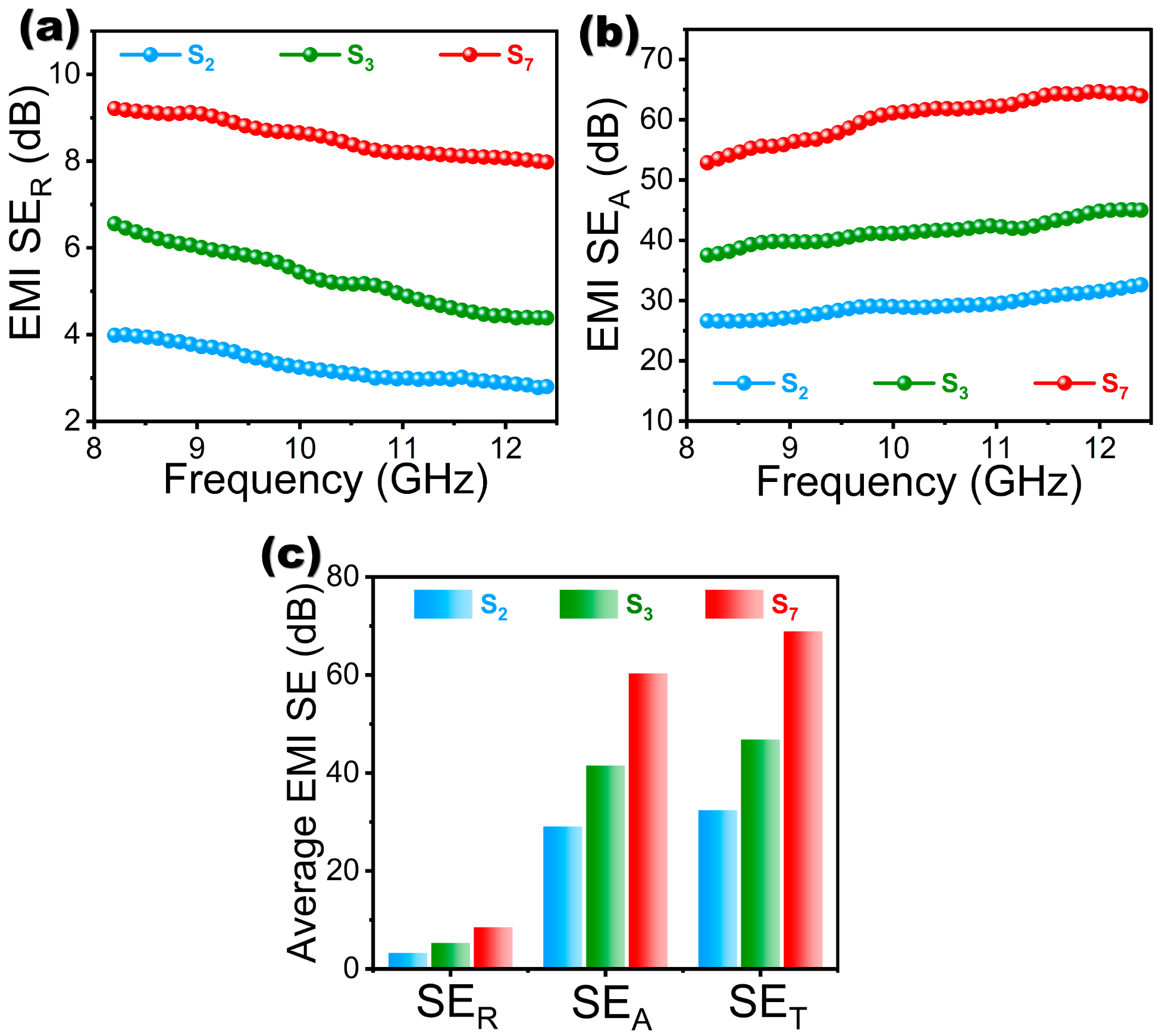


**Fig. S33 a** EMI SER, **b** SEA, and **c** average SE values and d EMI coefficients of (S2) carbonized PI/KNF aerogel/PW, (S3) C@CoNC/PW, and (S7) C/RGO-20@CoNC/PW composites at 100 °C

**Table S1** Comparison of the multifunctionality of the C/RGO-20@CoNC/PW composite with existing materials reported in the literature

| Smple | Solar-thermal conversion | Thermoelectric conversion | Electrothermal conversion | Magnetothermal conversion | EMI shielding | Refs. |
| --- | --- | --- | --- | --- | --- | --- |
| Wood aerogel@MXene @PA/PEG | √ | − | − | − | √ | [S1] |
| Carbonized loofah sponge/ phenolic Resin/PW | √ | − | √ | − | √ | [S2] |
| MXene/CoNi–C/PW | √ | − | ^−^ | − | √ | [S3] |
| Carbonized corn straw-ZIF/PW | √ | − | √ | √ | √ | [S4] |
| Nickel foam/carbon nanotubes/expanded graphite/PW | √ | − | − | √ | √ | [S5] |
| Nickel foam/lignin/rGO /PEG | √ | − | √ | − | √ | [S6] |
| Carbonized loofah-resin sponge/PW | √ | √ | − | − | √ | [S7] |
| PW/carbonized melamine foam/CoNC | √ | − | − | − | √ | [S8] |
| Graphitized graphene array/MXene-CNF /PEG | √ | √ | − | − | √ | [S9] |
| MXene@polydopamine/PEG | √ | − | − | √ | √ | [S10] |
| Graphitized chitosan/ graphene aerogel /PEG | √ | √ | − | − | − | [S11] - |
| Graphene aerogel film /PW | √ | √ | − | − | − | [S12] |
| Polyurethane-based s solid-solid PCMs/ graphene nanoplatelet | √ | − | − | − | − | [S13] |
| Modified graphene nanoplates /carboxylated carbon nanotubes/ poly(vinylidene fluoride)/ polydimethylsiloxane | √ | − | − | − | √ | [S14] |
| C/RGO-20@CoNC/PW | √ | √ | √ | √ | √ | This work |

Note: “√” means that the function is studied in the text; Conversely, “−” means that the function was not studied in the text.

**Table S2** List of abbreviations

| Codename | Sample composition name |
| --- | --- |
| PI/KNF/GO-20@ZIF-67 | PI + KNF + 20 wt% GO +ZIF-67 aerogel |
| C/RGO@CoNC | Carbonized PI/KNF/GO@ZIF-67 aerogel |
| PI/KNF/GO-20@ZIF-67/PW | PI/KNF/GO-20@ZIF-67 aerogel loaded with PW |
| Carbonized PI/KNF aerogel/PW | Carbonized PI/KNF complex aerogel loaded with PW |
| C@CoNC/PW | Carbonized PI/KNF@ZIF-67 complex aerogel loaded with PW |
| C/RGO-5@CoNC/PW | Carbonized PI/KNF/5 wt% GO@ZIF-67 complex aerogel loaded with PW |
| C/RGO-10@CoNC/PW | Carbonized PI/KNF/10 wt% GO@ZIF-67 complex aerogel loaded with PW |
| C/RGO-15@CoNC/PW | Carbonized PI/ KNF/15 wt% GO@ZIF-67 complex aerogel loaded with PW |
| C/RGO-20@CoNC/PW | Carbonized PI/KNF/20 wt% GO@ZIF-67 complex aerogel loaded with PW |
| PW | Paraffin wax |
| PCMs | Phase change materials |
| GO | Graphene oxide |
| PI | Polyimide |
| KNF | Kevlar nanofiber |
| ZIF-67 | Zeolitic imidazolate framework-67 |
| Co | Cobalt |
| CoNC | Carbonied ZIF-67 |
| RGO | Reduced GO |
| PEG | Polyethylene glycol |
| LSPR | Localized surface plasmon resonance |
| SEM | Scanning electron microscopy |
| XRD | X-ray powder diffraction |
| FTIR | *Fourier*-transform infrared ( |
| EDX | Energy-dispersive *X*–ray |
| XRD | *X*-ray diffraction |
| XPS | *X*–ray photoelectron spectroscopy |
| DSC | Differential scanning calorimetry |
| TEM | Transmission electron microscopy |
| *T*_m_ | Melting temperature |
| *T*_c_ | Crystallization temperature |
| Δ*H*_m_ | Melting enthalpy |
| Δ*H*_c_ | Crystallization enthalpy |
| TGA | Thermogravimetric analysis |
| PAAs | Polyamic acid ammonium salt |
| TEA | Triethylamine |
| ODA | *4,4*'-Diaminodiphenyl ether |
| PMDA | Pyromellitic dianhydride |
| PPTA | Poly(p-phenylene terephthamide) |
| *2*-MI | *2*-dimethylimidazole |
| DMSO | Dimethyl sulfoxide |
| STEG | Solar thermoelectric generator |
| LED | Light emitting diode |
| *U²* | Square output voltage |
| SE | Shielding effectiveness |
| SE_R_ | Reflection SE |
| SE_A_ | Absorption SE |
| R | Reflection coefficient |
| A | Absorption coefficient |
| T | Transmission coefficient |

**Table S3** Porous parameters and densities of PI/KNF, PI/KNF/GO-20@ZIF-67 and carbonized aerogels

| Samples | Average pore diameter  (μm) | Specific surface area  (m^2^ g^−1^) | Total intrusion volume  (mL/g) | Porosity  (%) | Density  (mg cm^−3^) |
| --- | --- | --- | --- | --- | --- |
| PI/KNF | 65.30 | 7.26 | 23.96 | 89.75 | 47.5 |
| PI/KNF/GO-20@ZIF-67 | 50.94 | 31.75 | 14.22 | 88.56 | 55.6 |
| C@CoNC | 38.74 | 25.35 | 10.38 | 85.52 | 40.5 |
| C/RGO-5@CoNC | 40.05 | 14.21 | 11.89 | 86.03 | 45.9 |
| C/RGO-10@CoNC | 42.15 | 15.46 | 12.35 | 86.69 | 51.2 |
| C/RGO-15@CoNC | 43.36 | 18.96 | 14.28 | 87.25 | 55.8 |
| C/RGO-20@CoNC | 45.43 | 20.78 | 15.75 | 87.82 | 59.7 |

**Table S4** Thermal energy storage properties of different pure PW and phase-change composites

| Code | Samples | Δ*H*_m_  (J/g) | Δ*H*_c_  (J/g) | *T*_m_  (°C) | *T*_c_  (°C) | *w*  (%) | *η*  (%) | *λ*  (%) |
| --- | --- | --- | --- | --- | --- | --- | --- | --- |
| S_0_ | Pure PW | 227.2 | 224.2 | 44.3 | 36.8 | – | – | – |
| S_1_ | PI/KNF/GO-20@ZIF-67/PW | 210.4 | 208.2 | 46.3 | 36.8 | 95.9 | 92.6 | 95.7 |
| S_2_ | Carbonized PI/KNF aerogel/PW | 204.2 | 203.4 | 46.1 | 36.7 | 93.2 | 89.9 | 95.9 |
| S_3_ | C@CoNC/PW | 199.9 | 199.6 | 45.9 | 37.5 | 94.0 | 88.0 | 94.2 |
| S_4_ | C/RGO-5@CoNC/PW | 201.5 | 201.1 | 45.5 | 37.1 | 94.3 | 88.7 | 94.6 |
| S_5_ | C/RGO-10@CoNC/PW | 205.4 | 204.4 | 45.3 | 37.4 | 95.6 | 90.4 | 95.0 |
| S_6_ | C/RGO-15@CoNC/PW | 208.7 | 206.9 | 45.2 | 37.3 | 96.1 | 91.9 | 95.8 |
| S_7_ | C/RGO-20@CoNC/PW | 209.2 | 208.4 | 45.0 | 37.9 | 96.4 | 92.1 | 96.0 |

**Table S5** Physical parameter values of the photothermal conversion efficiency of phase-change composites

| Samples  code | Δ*H*_m_  (J g^−1^) | *M*  (g) | *P*  (W m^−2^) | *S*  (m^2^) | *t*_1_  (s) | *t*_2_  (s) | *Q*  (J) | *η*  (%) |
| --- | --- | --- | --- | --- | --- | --- | --- | --- |
| S_0_ | 227.2 | 0.685 | 1000 | 3.8×10^−4^ | 138 | 865 | 727 | 56.3 |
| S_1_ | 210.4 | 0.757 | 1000 | 3.8×10^−4^ | 132 | 753 | 621 | 67.5 |
| S_2_ | 204.2 | 0.816 | 1000 | 3.5×10^−4^ | 126 | 697 | 571 | 83.4 |
| S_3_ | 199.9 | 0.9354 | 1000 | 4.2×10^−4^ | 172 | 680 | 508 | 87.6 |
| S_4_ | 201.5 | 0.7601 | 1000 | 3.6×10^−4^ | 190 | 668 | 478 | 89 |
| S_5_ | 205.4 | 0.6615 | 1000 | 3.5×10^−4^ | 118 | 540 | 422 | 92 |
| S_6_ | 208.7 | 0.6725 | 1000 | 4×10^−4^ | 129 | 505 | 376 | 93.3 |
| S_7_ | 209.2 | 0.6398 | 1000 | 4×10^−4^ | 132 | 484 | 352 | 95.1 |

Note: The solar-thermal conversion efficiency (η) of phase-change composite samples can be calculated by using the following equation S11: $\eta=\frac{m\times\Delta H_{m}}{P\times S\times(t_{2}-t_{1})}\times100\%$ (S11)

where *m* is the mass, g; Δ*H*_m_ is the melting enthalpy, J g^−1^; *S* is the surface area of PEG composites, m^2^; *P* is the sunlight intensity, kW m^−2^ and *t*_1_ and *t*_2_ are the start and end time of the sample phase transformation, respectively.

**Table S6** Comparison of thermal stability, solar-thermal conversion efficiency and electromagnetic interference (EMI) shielding effectiveness of the C/RGO-20@CoNC/PW composite with those of other relevant materials reported in the literature

| Sample code | Thermal degradation temperature (°C) | Solar-thermal conversion efficiency (%) | EMI shielding effectiveness (dB) | Band frequency range (GHz) | Refs. |
| --- | --- | --- | --- | --- | --- |
| Wood aerogel@MXene @PA/PEG | 210.5–425.7 | 98.6 | 43.94 | 8.2–12.4 | [S1] |
| Loofah sponge porous carbon/PW | – | 76.0 | 32 | 8.2–12.4 | [S2] |
| Styrene-ethylene-propylene-styrene/ NdFeB@Ag/PW | 165.0–350 | – | 35.42 | 8.2–12.4 | [S15] |
| MXene-K^+^ aerogel/PW | 205.8–45.4 | 98.4 | 57.7 | 8.2–12.4 | [S16] |
| MXene/starch hybrid aerogels/*n*-eicosane | 170.0–270.0 | 88.4 | 34.77 | 8.2–12.4 | [S17] |
| Monolithic porous carbon @Ni Al-LDH skeleton/PEG | 100.0–310.0 | 90.6 | 38.8 | 8.2–12.4 | [S18] |
| MXene/delignified wood/*n*-eicosane | 207.7–214.7 | 88.4 | 34.12 | 8.2–12.4 | [S19] |
| PF/porous carbon/PEG | 125.0–350.0 | 89.4 | 59.83 | 8.2–12.4 | [S20] |
| Carbon mesh/CuS/ZnO/stearic acid | 257.3–306.0 | 88.9 | 40.8 | 8.2–12.4 | [S21] |
| C/RGO-20@CoNC/PW | 280–430 | 95.4 | 66.2 | 8.2–12.4 | This work |

**Table S7** The manufacturing cost of a C/RGO-20@CoNC/PW composite with dimensions of 30 × 30 × 30 mm^3^ (Length × Width × Height)

| Experimental Materials and Reagents | Unit price | Amount | Total price |
| --- | --- | --- | --- |
| 4,4′-Diaminodiphenyl ether | 66.7 $ kg^−1^ | 1.02 g | 0.068 $ |
| Pyromellitic dianhydride | 84.7 $ kg^−1^ | 1.12 g | 0.095 $ |
| N-N-Dimethylacetamide | 14.4 $ L^−1^ | 20 mL | 0.288 $ |
| Cobalt nitrate hexahydrate | 49.2 $ kg^−1^ | 0.58 g | 0.029 $ |
| Triethylamine | 23.1 $ L^−1^ | 4.5 mL | 0.104 $ |
| Poly(p-phenylene terephthalamide) fiber | 79.2 $ kg^−1^ | 0.2 g | 0.016 $ |
| Potassium hydroxide | 83.2 $ kg^−1^ | 0.2 g | 0.017 $ |
| Dimethyl sulfoxide | 35.1 $ L^−1^ | 20 mL | 0.07 $ |
| Graphite powder | 40.0 $ kg^−1^ | 2 g | 0.08 $ |
| Sodium nitrate | 38.8 $ kg^−1^ | 1 g | 0.039 $ |
| Potassium persulfate | 26.3 $ kg^−1^ | 3 g | 0.079 $ |
| Phosphorus pentoxide | 9.8 $ kg^−1^ | 3 g | 0.029 $ |
| Sulfuric acid | 12.2 $ L^−1^ | 25 mL | 0.305 $ |
| Potassium permanganate | 27.4 $ kg^−1^ | 5 g | 0.137 $ |
| Polyvinylpyrrolidone | 32.4 $ kg^−1^ | 0.3 g | 0.009 $ |
| *2*-methylimidazole | 24.5 $ kg^−1^ | 1.23 g | 0.031 $ |
| H_2_O_2_ | 13.8 $ L^−1^ | 20 mL | 0.276 $ |
| PW | 41.7 $ kg^−1^ | 5 g | 0.208 $ |
| Total | – | – | 1.88 $ |

**Supplementary References**

1. Y. Chen, Y. Meng, J. Zhang, Y. Xie, H. Guo et al., Leakage proof, flame-retardant, and electromagnetic shield wood morphology genetic composite phase change materials for solar thermal energy harvesting. Nano-Micro Lett. **16**(1), 196 (2024). <https://doi.org/10.1007/s40820-024-01414-4>
2. H. He, Y. Wang, Z. Zhao, Q. Wang, Q. Wei et al., Dual-encapsulated multifunctional phase change composites based on biological porous carbon for efficient energy storage and conversion, thermal management, and electromagnetic interference shielding. J. Energy Storage **55**, 105358 (2022). <https://doi.org/10.1016/j.est.2022.105358>
3. Y. Gao, X. Chen, X. Jin, C. Zhang, X. Zhang et al., Multifunction integration within magnetic CNT-bridged MXene/CoNi based phase change materials. eScience **4**(6), 100292 (2024). <https://doi.org/10.1016/j.esci.2024.100292>
4. S. Liu, B. Quan, M. Sheng, Y. Yang, X. Hu et al., A novel *in situ* growth ZIF-67 on biological porous carbon encapsulated phase change composites with electromagnetic interference shielding and multifunctional energy conversion. Nano Energy **114**, 108669 (2023). <https://doi.org/10.1016/j.nanoen.2023.108669>
5. X. Hu, B. Ai, B. Quan, Z. Shi, Z. Liu et al., Synergistically networked 1D/2D/3D phase change composites with Ultra-Fast contactless thermal energy conversion and enhanced electromagnetic interference shielding performance. Chem. Eng. J. **504**, 158915 (2025). <https://doi.org/10.1016/j.cej.2024.158915>
6. R. Yan, Z. Huang, Y. Chen, L. Zhang, X. Sheng, Phase change composite based on lignin carbon aerogel/nickel foam dual-network for multisource energy harvesting and superb EMI shielding. Int. J. Biol. Macromol. **277**, 134233 (2024). <https://doi.org/10.1016/j.ijbiomac.2024.134233>
7. H. He, M. Dong, Q. Wang, J. Zhang, Q. Feng et al., A multifunctional carbon-base phase change composite inspired by “fruit growth”. Carbon **205**, 499–509 (2023). <https://doi.org/10.1016/j.carbon.2023.01.038>
8. Y. Li, X. Diao, P. Li, P. Liu, Y. Gao et al., Advanced multifunctional Co/N Co-doped carbon foam-based phase change materials for wearable thermal management. Chem. Eng. J. **485**, 149858 (2024). <https://doi.org/10.1016/j.cej.2024.149858>
9. B. Hu, H. Guo, T. Li, X. Cao, M. Cao et al., Engineering tiramisu-like phase change nanocomposite for superior thermal energy management and electromagnetic interference shielding. J. Mater. Sci. Technol. **206**, 113–124 (2025). <https://doi.org/10.1016/j.jmst.2024.04.021>
10. X. Hu, B. Quan, B. Ai, M. Sheng, S. Liu et al., Engineering asymmetric multifunctional phase change composites for improved electromagnetic interference shielding and wireless personal thermal therapy. J. Mater. Chem. A **11**(30), 16138–16152 (2023). <https://doi.org/10.1039/D3TA03184E>
11. H.-Y. Zhao, C. Shu, X. Wang, P. Min, C. Li et al., Bioinspired intelligent solar-responsive thermally conductive pyramidal phase change composites with radially oriented layered structures toward efficient solar–thermal–electric energy conversion. Adv. Funct. Mater. **33**(33), 2302527 (2023). <https://doi.org/10.1002/adfm.202302527>
12. H.-Y. Zhao, X. Wang, J. Wu, C. Shu, F.-L. Gao et al., An intelligent, solar-responsive, and thermally conductive phase-change system toward solar-thermal-electrical conversion featuring daytime blooming for solar energy harvesting and nighttime closing for thermal preservation. Adv. Funct. Mater. **34**(45), 2406236 (2024). <https://doi.org/10.1002/adfm.202406236>
13. X. Geng, M. Qin, Z. Shen, F. Xiong, J. Di et al., Muscle-inspired super-flexible phase change materials with programmable deformation for photothermal actuation. Adv. Funct. Mater. 2418848 (2024). <https://doi.org/10.1002/adfm.202418848>
14. C. Jiang, C. Hao, C. Zi, J. Li, W. Liu et al., Electrical and thermal conductive composites with thermal management and electromagnetic shielding enhanced by 3D network. Compos. Sci. Technol. **265**, 111135 (2025). <https://doi.org/10.1016/j.compscitech.2025.111135>
15. H. Guo, B. Hu, H. Shan, Z. Li, W. Qi et al., Magnetically assembled flexible phase change composites with vertically aligned structures for thermal management and electromagnetic interference shielding. Chem. Eng. J. **495**, 153361 (2024). <https://doi.org/10.1016/j.cej.2024.153361>
16. C. Zhu, Y. Hao, H. Wu, M. Chen, B. Quan et al., Self-assembly of binderless MXene aerogel for multiple-scenario and responsive phase change composites with ultrahigh thermal energy storage density and exceptional electromagnetic interference shielding. Nanomicro Lett. **16**(1), 57 (2023). <https://doi.org/10.1007/s40820-023-01288-y>
17. Y. Liu, J. Tang, J. Wang, H. Yue, Z. Du et al., Nb2CTx MXene/starch hybrid aerogels supported flame-retardant phase change composites with superior solar-thermal conversion efficiency and outstanding electromagnetic interference shielding. Compos. Part A Appl. Sci. Manuf. **193**, 108854 (2025). <https://doi.org/10.1016/j.compositesa.2025.108854>
18. S. Wang, Z. Sun, Y. Wang, T. Liang, B. Wang et al., Design of CuS composite carbon-based Ni Al-LDH multifunctional phase change composite with electromagnetic shielding performance and heat storage capacity. Chem. Eng. J. **491**, 151960 (2024). <https://doi.org/10.1016/j.cej.2024.151960>
19. H. Yue, Y. Ou, J. Wang, H. Wang, Z. Du et al., Ti_3_C_2_T*_x_* MXene/delignified wood supported flame-retardant phase-change composites with superior solar-thermal conversion efficiency and highly electromagnetic interference shielding for efficient thermal management. Energy **286**, 129441 (2024). <https://doi.org/10.1016/j.energy.2023.129441>
20. H. Fang, J. Zeng, X. Shao, D. Hu, Advanced electromagnetic shielding and excellent thermal management of flexible phase change composite films. Carbon **215**, 118442 (2023). <https://doi.org/10.1016/j.carbon.2023.118442>
21. Z. Zhou, Y. Huang, Q. Shen, Y. Li, X. Cheng, Composite phase change materials with carbon-mesh/CuS/ZnO interface biocarbon skeleton for solar energy storage, solar photocatalysis and electromagnetic shielding. J. Energy Storage **90**, 111937 (2024). <https://doi.org/10.1016/j.est.2024.111937>
